# Chiroptically Active Host–Guest Composites Using a Terpene-Based Micellar Capsule

**DOI:** 10.1021/jacs.4c07193

**Published:** 2024-08-19

**Authors:** Yoshihisa Hashimoto, Yuya Tanaka, Daiya Suzuki, Yoshitane Imai, Michito Yoshizawa

**Affiliations:** † Laboratory for Chemistry and Life Science, Institute of Innovative Research, Tokyo Institute of Technology, 4259 Nagatsuta, Midori-ku, Yokohama 226-8503, Japan; ‡ Graduate School of Science and Engineering, Kindai University, 3-4-1 Kowakae, Higashi-Osaka, Osaka 577-8502, Japan

## Abstract

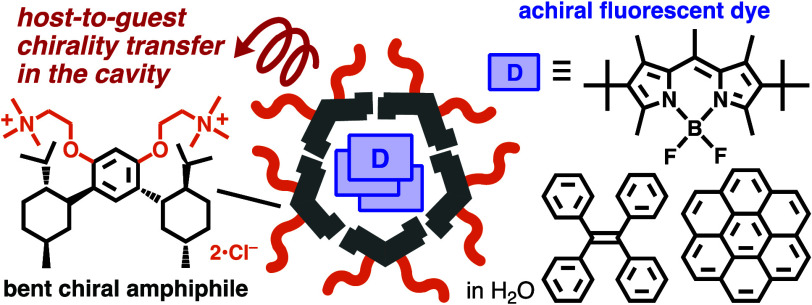

For the design of a new chiroptically active host–guest system, a bent amphiphilic compound was synthesized using cyclic monoterpenes as key biorelated chiral frameworks. In water, the bent amphiphiles form a terpene-based micellar capsule with a core diameter of ∼2 nm in a spontaneous and quantitative fashion. The resultant chiral capsule shows wide-ranging uptake abilities toward achiral fluorescent dyes in water. Notably, relatively strong CD bands are generated from the resultant host–guest composites, e.g., possessing AIE-active tetraphenylethene and sterically demanding BODIPY dyes, through efficient host-to-guest chirality transfer. The composites also display CPL, with moderate to high emission asymmetry factors (|*g*_lum_| = up to 3.3 × 10^–3^).

A rational combination of synthetic organic dyes and chiral groups generates chiroptically active components, exhibiting characteristic circular dichroism (CD) and circularly polarized luminescence (CPL).^1^ These physicochemical properties, related to advanced optical technologies (e.g., storage, display, and analysis), can be largely enhanced in supramolecular systems.^2^ Infinite helical stacks of aromatic dyes with chiral groups/centers are typical examples (Figure 1a), in which monomer chiralities are effectively amplified via noncovalent interactions. Supramolecular cages and capsules, bearing *finite chiral cavities* (Figure 1b), are another strategy to develop novel chiroptically active systems.^3^ The use of noncovalent host–guest interactions in the cavities is highly expected to generate unusual chiroptical features in a facile and tunable fashion. There have been several reports on supramolecular hosts with chiral cavities, formed via coordinative,^4,5^ hydrogen bonding,^6^ and π-stacking interactions.^7^ However, their guest scopes are relatively narrow owing to the *rigid* host frameworks and weak host–guest interactions. The design principle for efficient host-to-guest chirality transfer also remains unestablished. To develop a supramolecular capsule with novel chiroptically active host functions, here we report micellar capsule (**MA**)_*n*_ providing a flexible chiral cavity, surrounded by terpene-based bent amphiphiles (Figure 1c). The capsule offers (i) high uptake ability toward achiral fluorescent dyes with various sizes and shapes in water, (ii) efficient optical chirality transfer from the host framework to the guest dyes, which is enhanced (∼3-fold) upon a thermal stimulus, and (iii) moderate to high circularly polarized luminescence from the dyes in the chiral cavity.

**Figure 1:**
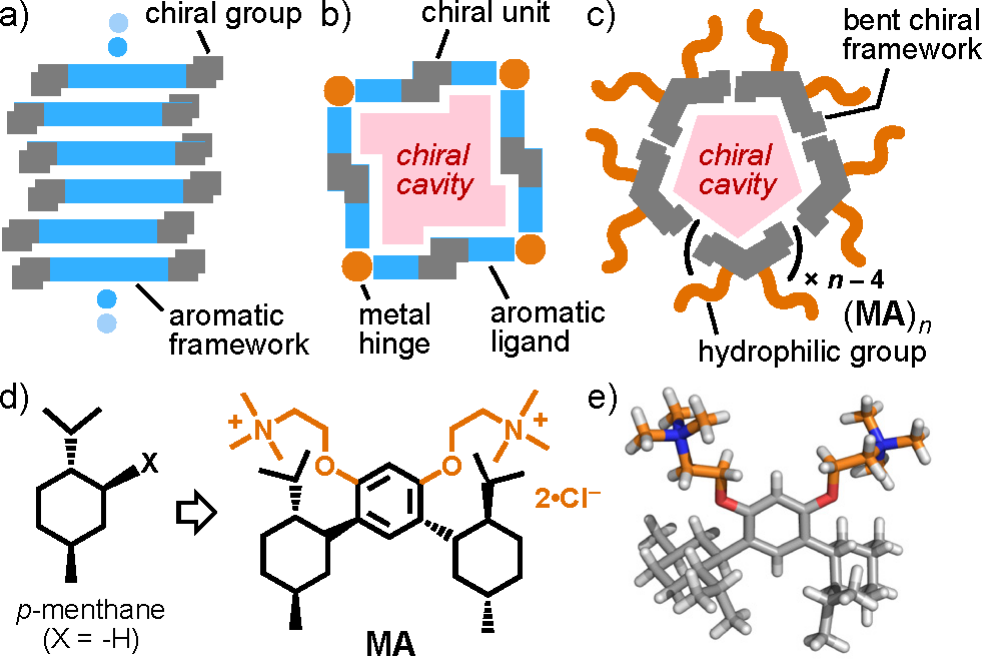
Chiroptically active supramolecular systems: (a) infinite helical stack, and (b) coordination cage and (c) micellar capsule (**MA**)_*n*_ with a chiral cavity. (d) Bent amphiphile **MA** and (e) its crystal structure.^8^

To design a new chiral capsule with a *well-defined yet flexible* host framework, we herein focused a cyclic monoterpene, i.e., *p*-menthane (X = H; Figure 1d, left), as a key prochiral compound found in natural products.^10^ Chiral *p*-menthyl chloride (X = Cl) is readily accessible from menthol (X = OH)^11^ and used for the preparation of chiral host subunit **MA** (Figure 1d, right). As compared with other cyclic alkanes such as cyclohexyl and adamantyl groups,^12^ the terpene framework provides a wider and relatively rigid hydrophobic surface with equatorial methyl and isopropyl groups, for efficient intermolecular interactions (Figure 1e and Figures S20 and S21).^9^ The absence of UV-to-visible-light absorption (>∼300 nm), unlike typical aromatic-based chiral groups (e.g., binaphthyl and helicenyl groups), is furthermore beneficial for the study of guest-based emission. It is noteworthy that there has been surprisingly no report on the use of chiral cyclic monoterpenes in supramolecular host structures so far.^13^ Bent amphiphilic compounds with two hydrophobic (poly)aromatic/cycloalkyl frameworks are useful subunits for the quantitative formation of micellar capsules in water.^12,14^ The simple replacement of the previous achiral groups with the chiral menthyl group on the amphiphile, for the first time, succeeds in the formation of chiral capsule (**MA**)_*n*_.^15^

Synthesis of chiral subunit **MA** and its enantiomer (**MA**^**E**^) was completed in six steps starting from (−)- or (+)-menthol, including Negishi coupling as a key step.^8,9,16^ Micellar capsule (**MA**)_*n*_ was spontaneously and quantitatively formed upon the simple addition of **MA** (1.3 mg, 13 μmol) to water (0.5 mL) at room temperature. Unlike the ^1^H NMR spectrum of **MA** in DMSO-*d*_6_ (Figure 2a), that of (**MA**)_*n*_ in D_2_O exhibited two sets of shifted aromatic signals and broadened aliphatic signals in the range 0.40–1.80 ppm, attributed to the menthyl groups (Figure 2c), suggesting the quantitative capsule formation.^17^ The DLS analysis of the resultant aqueous solution indicated the average core diameter of (**MA**)_*n*_ being 1.7 nm (Figure 2d). The combination of the experimental and molecular modeling studies proposed the formation of hexamer (**MA**)_6_ with a spherical menthyl core as an average product (Figure 2e and Figure S28).^18^ The chiroptical properties of the product in water were confirmed by the concentration-dependent CD studies, owing to the relatively high CMC value against the used CD detector.^9,17^ In the CD spectrum (12 mM based on **MA**), intense positive Cotton effects were observed at 250–300 nm (Figure 2g), in the range of the phenylene-based absorption band derived from **MA** (Figure 2f). Enantiomeric capsule (**MA**^**E**^)_*n*_ was also formed from **MA**^**E**^ in water (Figure 2f,g).

**Figure 2:**
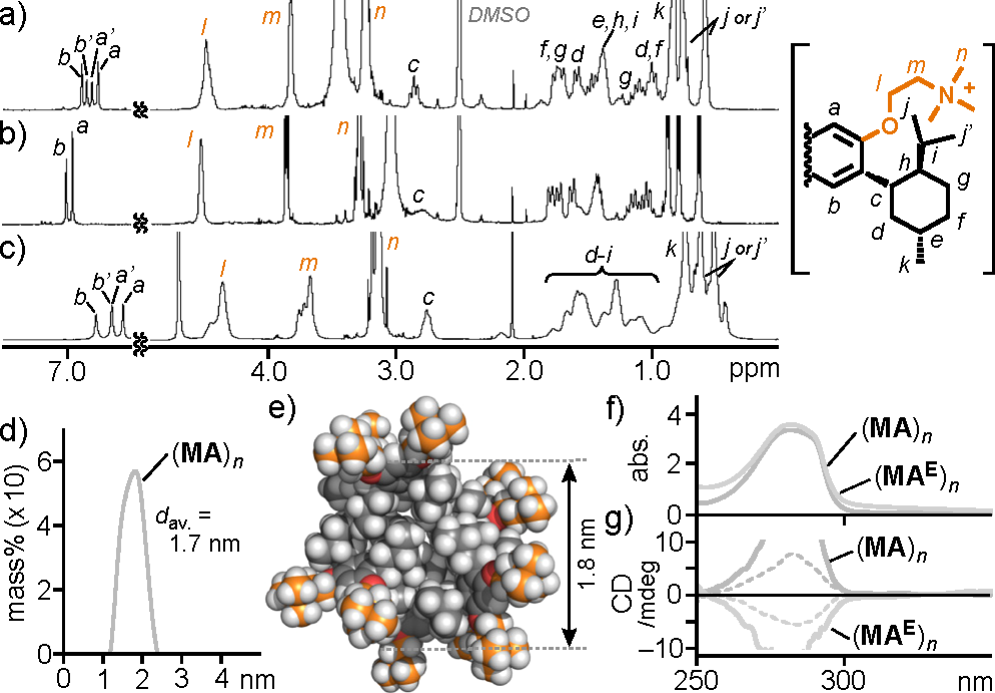
^1^H NMR spectra (400 MHz, 25 mM based on **MA**) of **MA** in DMSO-*d*_6_ at (a) r.t. and (b) 80 °C, and (c) (**MA**)_*n*_ in D_2_O at r.t. (d) DLS chart (H_2_O, r.t., 25 mM based on **MA**) of (**MA**)_*n*_. (e) Optimized structure of (**MA**)_6_.^18^ (f) UV–vis spectra (12 mM based on the subunit) of (**MA**)_*n*_ and (**MA**^**E**^)_*n*_ and (g) their CD spectra (12 mM (solid lines) and 5.0 mM (dotted lines)) in H_2_O at r.t.

To clarify the uptake ability of capsule (**MA**)_*n*_ toward fluorescent dyes and the chiral properties of the resultant host–guest composites, tetraphenylethene (**TPE**) and hexaphenylsilole (**HPS**) were employed as hydrophobic AIE-active molecules.^19^ As the optimized procedure, a mixture of **MA** and **TPE** (2.0 μmol each) was manually ground for 2 min using an agate mortar and pestle.^9^ The mixed solid was dissolved in H_2_O (2.0 mL) at room temperature. The centrifugation and filtration of the suspended mixture gave rise to a clear colorless solution, including host–guest composite (**MA**)_*n*_·(**TPE**)_*m*_ (Figure 3a). The UV–vis spectrum showed new broad absorption bands at <270 and 320 nm, derived from (**TPE**)_*m*_ within the capsule (Figure 3b, top). The CD spectrum of (**MA**)_*n*_·(**TPE**)_*m*_ exhibited positive and negative Cotton effects in the range 250 to 340 nm with a moderate absorption dissymmetry factor (|*g*_abs_| = 2.7 × 10^–4^ at 259 nm), whose band intensity is higher than that of (**MA**)_*n*_ (>3-fold) at the same host concentration (1.0 mM based on **MA**; Figure 3c). The mirror symmetric spectrum was observed from the enantiomer (**MA**^**E**^)_*n*_·(**TPE**)_*m*_. In contrast, no Cotton effect was detected in the CD spectra of related, nonchiral hosts including **TPE** dyes, such as (**PBA**^20^ or **CHA** or **SDS**)_*n*_·(**TPE**)_*m*_, under the same conditions (Figure 3d and Figure S38). These results demonstrated efficient chirality transfer from capsule (**MA**)_*n*_ to dye aggregate (**TPE**)_*m*_ through guest uptake. When host–guest composite (**MA**)_*n*_·(**HPS**)_*m*_ was obtained in a manner similar to (**MA**)_*n*_·(**TPE**)_*m*_, the CD study verified moderate host–guest chirality transfer (|*g*_abs_| = 1.9 × 10^–4^ at 258 nm) under the same conditions (Figure S47).^9,21^

**Figure 3:**
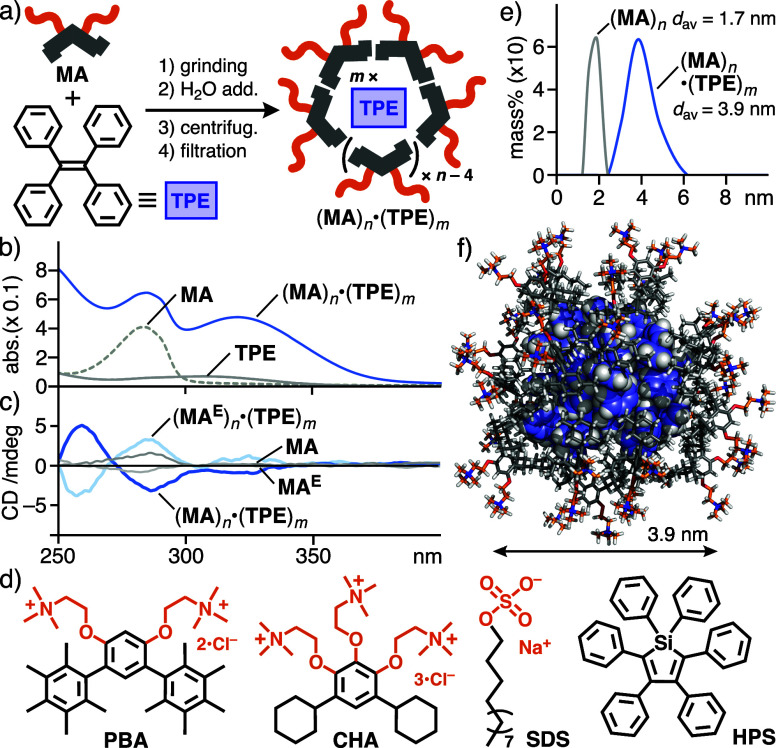
(a) Formation of host–guest composite (**MA**)_*n*_·(**TPE**)_*m*_ in water. (b) UV–vis spectra (H_2_O, r.t., 1.0 mM based on **MA**) of (**MA**)_*n*_·(**TPE**)_*m*_, **MA**, and **TPE** in CH_2_Cl_2_ (0.1 mM) and (c) the CD spectra of (**MA** or **MA**^**E**^)_*n*_·(**TPE**)_*m*_, **MA**, and **MA**^**E**^ under the same conditions. (d) Bent and linear amphiphiles studied herein and **HPS**. (e) DLS charts (H_2_O, r.t., 25/1.0 mM based on **MA**) of (**MA**)_*n*_ and (**MA**)_*n*_·(**TPE**)_*m*_. (f) Optimized structure of (**MA**)_21_·(**TPE**)_12_.^18^

The structure of (**MA**)_*n*_·(**TPE**)_*m*_ was revealed by a combination of DLS, UV–vis, NMR, and molecular modeling studies. The DLS measurement of (**MA**)_*n*_·(**TPE**)_*m*_ indicated the formation of small particles with an average core diameter of 3.9 nm (Figure 3e). The average **MA**:**TPE** ratio of the particles was estimated to be 7:4 on the basis of the ^1^H NMR integral of isolated (**MA**)_*n*_·(**TPE**)_*m*_ in CDCl_3_ after lyophilization (Figure S31b).^9^ Molecular modeling studies suggested that the product structure is composed of (**MA**)_21_·(**TPE**)_12_ in a spherical fashion, providing a core diameter of 3.9 nm (Figure 3f),^18^ due to efficient host–guest CH−π interactions and the hydrophobic effect in the cavity.

Capsule (**MA**)_*n*_ also bound planar polyaromatic as well as sterically demanding fluorescent dyes in the cavity and subsequently displayed chirality-transfer-based CD bands in water. After the uptake procedure using **MA** with coronene (**Cor**) or perylene (**Per**), the corresponding host–guest composites (**MA**)_*n*_·(**Cor**)_*m*_ and (**MA**)_*n*_·(**Per**)_*m*_ were obtained as clear aqueous solutions (Figure 4a).^9^ In the same way, the treatment of **MA** with di(*tert*-butyl)pentamethyl BODIPY (**DBB**) and its pentamethyl analogue (**PMB**) gave rise to (**MA**)_*n*_·(**DBB**)_*m*_ and (**MA**)_*n*_·(**PMB**)_*m*_, respectively (Figure 4c,e).^9^ The UV–vis spectra showed new absorption bands derived from the dyes with −40 or +10 nm shifts, indicating their efficient encapsulation within the capsule.^9,22^ Host–guest composites (**MA**)_*n*_·(**Cor**)_*m*_ and (**MA**)_*n*_·(**Per**)_*m*_ provided moderate Cotton effects at 270–320 nm (|*g*_abs_| = 6.7 × 10^–5^ at 292 nm) and 350–480 nm (1.9 × 10^–4^ at 458 nm), respectively (Figure 4b). These |*g*_abs_| values are 2–4 times lower than that of (**MA**)_*n*_·(**TPE**)_*m*_ (Figure 5a), owing to inefficient chirality transfer to the symmetrical/asymmetrical disc-shaped dyes. The dissymmetry factor derived from (**MA**)_*n*_·(**DBB**)_*m*_ (|*g*_abs_| = 1.9 × 10^–4^ at 484 nm) is comparable to that of (**MA**)_*n*_·(**Per**)_*m*_ (Figure 4d) yet much higher than that of *tert*-butyl-free (**MA**)_*n*_·(**PMB**)_*m*_ under the same conditions (|*g*_abs_| = <1.0 × 10^–5^; Figure 4d, inset). These results indicate that the steric bulkiness of organic dyes is important for effective host-to-guest, optical chirality transfer through efficient van der Waals interactions between the host and guest aliphatic frameworks in this system. Interestingly, a large band enhancement was found in the CD spectrum of (**MA**)_*n*_·(**DBB**)_*m*_ at room temperature, after heating the aqueous solution at 80 °C for 10 min (Figure 4d). On the basis of the absorption dissymmetry factor at 487 nm (|*g*_abs_| = 6.4 × 10^–4^), the guest-based CD band increased 3.3-fold through the thermal stimulus (Figure 5a). This unusual behavior, which was not observed with the other host–guest composites studied herein, most probably stems from the rearrangement of the guest aggregate within the capsule.^23^ The product sizes of (**MA**)_*n*_·(**Cor**)_*m*_ and (**MA**)_*n*_·(**DBB**)_*m*_ were estimated from the DLS analysis (Figure 4f,g), and their composition ratios were revealed by the NMR studies.^24^ The molecular modeling suggested the formation of (**MA**)_14_·(**DBB**)_10_ as an average structure (Figure 4h).

**Figure 4:**
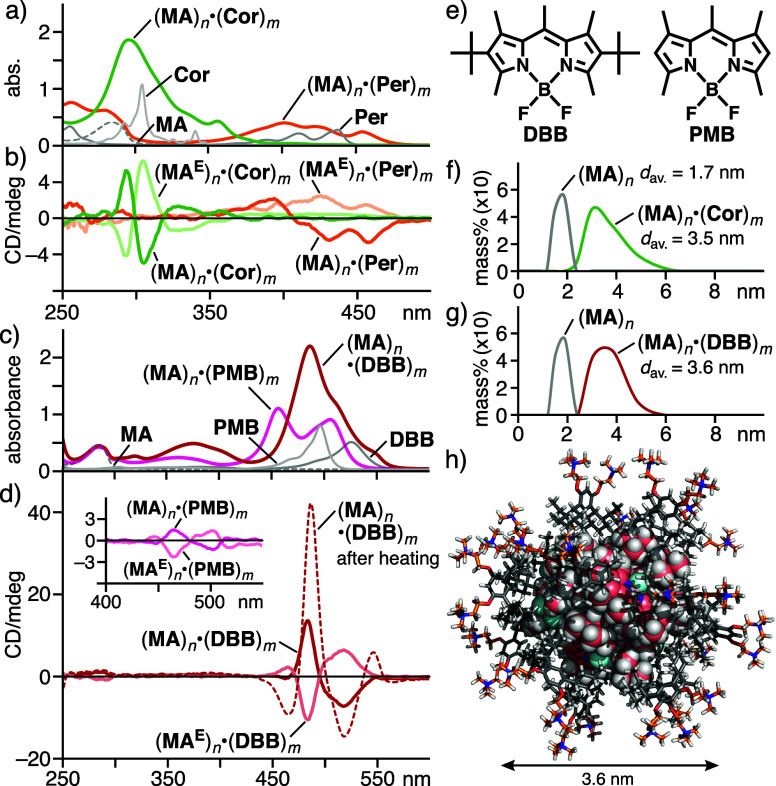
(a) UV–vis spectra (H_2_O, r.t., 1.0 mM based on **MA**) of (**MA**)_*n*_·(**Cor** or **Per**)_*m*_, **MA**, **Cor**, and **Per** in CH_2_Cl_2_ (0.1 mM) and (b) the CD spectra of (**MA** or **MA**^**E**^)_*n*_·(**Cor** or **Per**)_*m*_ under the same conditions. (c) UV–vis spectra (H_2_O, r.t., 1.0 mM based on **MA**) of (**MA**)_*n*_·(**DBB** or **PMB**)_*m*_, **MA**, **DBB**, and **PMB** in CH_2_Cl_2_ (0.1 mM) and (d) CD spectra of (**MA** or **MA**^**E**^)_*n*_·(**DBB** or **PMB**)_*m*_ and (**MA**)_*n*_·(**DBB**)_*m*_, after heating at 80 °C, under the same conditions. (e) Dyes **DBB** and **PMB**. DLS charts (H_2_O, r.t., 25/1.0 mM based on **MA**) of (f) (**MA**)_*n*_ and (**MA**)_*n*_·(**Cor**)_*m*_, and (g) (**MA**)_*n*_ and (**MA**)_*n*_·(**DBB**)_*m*_. (h) Optimized structure of (**MA**)_14_·(**DBB**)_10_.^18^

**Figure 5:**
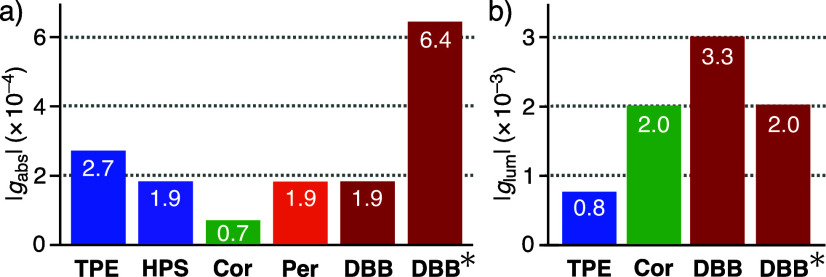
(a) Absolute absorption dissymmetry factors (H_2_O, r.t., 1.0 mM based on **MA**) of host–guest composites (**MA**)_*n*_ including dyes **TPE**, **HPS**, **Cor**, **Per**, and **DBB**, respectively (*: after heating at 80 °C), and (b) the selected absolute emission asymmetry factors.

Prompted by the efficient chirality transfer within the host–guest composites observed above, finally, their photoluminescence (PL) and CPL properties were elucidated under ambient aqueous conditions. Free **TPE** and **HPS** in CH_2_Cl_2_ showed weak or no PL with emission quantum yields (Φ_F_) of 0–1%, upon irradiation at 320 and 366 nm, respectively (Figures S39 and S49). Whereas capsule (**MA**)_*n*_ itself also showed weak PL (Φ_F_ = 10%) in water, the aqueous solutions of (**MA**)_*n*_·(**TPE**)_*m*_ and (**MA**)_*n*_·(**HPS**)_*m*_ emitted relatively strong blue fluorescence with Φ_F_ = 20% (λ_max_ = 450 nm) and 63% (λ_max_ = 485 nm), respectively (Figure 6a and Figures S39 and S49). The quantum yields are significantly higher as compared with those in organic solvent, due to unusual encapsulation-induced AIE behavior.^25^ Unlike the usual AIE systems in *mixed solvent*, (**MA**)_*n*_·(**TPE**)_*m*_ and (**MA**)_*n*_·(**HPS**)_*m*_ could be handled in 100% water. The CPL spectra of (**MA**)_*n*_·(**TPE**)_*m*_ and (**MA**)_*n*_·(**HPS**)_*m*_ as well as their **MA**^**E**^-based isomers displayed roughly symmetric mirror-like bands in the ranges 400–550 and 420–550 nm (Figure 6d and Figure S53), respectively. The absolute emission asymmetry factors (|*g*_lum_|) of the host–guest composites including (**TPE**)_*m*_ and (**HPS**)_*m*_ were estimated to be 0.8 × 10^–3^ and 1.7 × 10^–4^, respectively. In the same way, the PL and CPL studies of (**MA**)_*n*_·(**Cor**)_*m*_ and (**MA**)_*n*_·(**Per**)_*m*_ in water showed green and orange fluorescence (Φ_F_ = 13 and 6%) with |*g*_lum_| = 2.0 × 10^–3^ and <10^–5^, respectively (Figure 6b,e and Figure S62).

**Figure 6:**
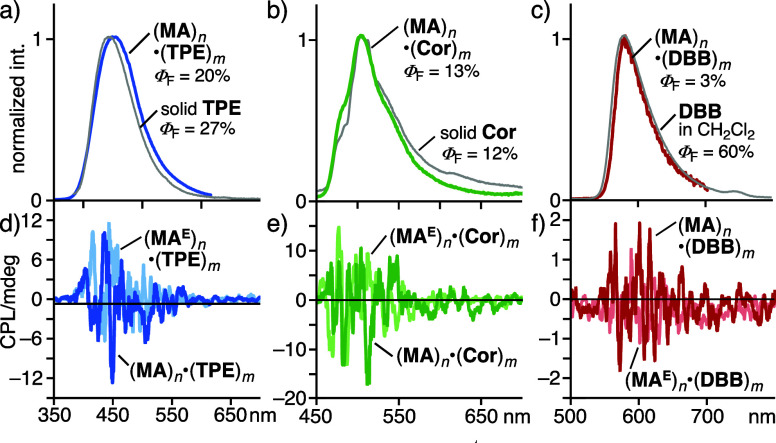
(a) Fluorescence spectra (H_2_O, r.t., 1.0 mM based on **MA**) of (**MA**)_*n*_·(**TPE**)_*m*_ and solid **TPE** (λ_ex_ = 320 nm), (b) (**MA**)_*n*_·(**Cor**)_*m*_ and solid **Cor** (λ_ex_ = 295, 405 nm, respectively), and (c) (**MA**)_*n*_·(**DBB**)_*m*_ and **DBB** in CH_2_Cl_2_ (0.1 mM; λ_ex_ = 320 nm), and their quantum yields. CPL spectra (H_2_O, r.t., 1.0 mM based on the subunit) of (d) (**MA**)_*n*_·(**TPE**)_*m*_, (e) (**MA**)_*n*_·(**Cor**)_*m*_, and (f) (**MA**)_*n*_·(**DBB**)_*m*_, and their enantiomers.

The highest |*g*_lum_| value was observed from BODIPY-based host–guest composite (**MA**)_*n*_·(**DBB**)_*m*_, among the composites studied herein. It should be noted that there have been many reports on covalent CPL systems so far, yet noncovalent host–guest composites, featuring both small size (e.g., *d* < 10 nm) and moderate to high CPL values (|*g*_lum_| > 10^–3^), are still rare in water.^26^ The aqueous solution of (**MA**)_*n*_·(**DBB**)_*m*_ emitted orange fluorescence with an emission band of λ_max_ = 586 nm (Φ_F_ = 3%; Figure 6c). The CPL bands were observed in the range 540 to 680 nm, and the |*g*_lum_| was estimated to be 3.3 × 10^–3^, which is 6- and 1.5-fold higher than that of (**MA**)_*n*_·(**TPE**)_*m*_ and (**MA**)_*n*_·(**Cor**)_*m*_, respectively (Figure 5b). A roughly mirror-like CPL spectrum was found in (**MA**^**E**^)_*n*_·(**DBB**)_*m*_ (Figure 6f).

In summary, we have developed a novel chiroptically active host–guest system using terpene-based, bent amphiphiles as a biorelated chiral subunit. A new micellar capsule with a well-defined, flexible chiral cavity formed in water from the amphiphiles in a spontaneous and quantitative fashion. The present capsular cavity facilitated the efficient uptake of various achiral fluorescent dyes, such as AIE-active, polyaromatic, and BODIPY compounds in water. The resultant host–guest composites exhibited strong CD bands, due to efficient noncovalent, optical chirality transfer from the host to guest dyes. Thanks to the nonaromatic chiral frameworks, the host–guest composites emitted efficient CPL with moderate to high emission asymmetry factors. Notably, the present system provides the following advantages. (i) The capsule allows facile preparation of well-defined host–guest composites (∼4 nm in diameter) with tunable chiroptical properties, through simple uptake of various achiral dyes, unlike the majority of previously reported hosts with rigid chiral cavities.^3−5^ (ii) The resultant host–guest composites can be used in 100% water, without covalent functionalization of the corresponding dyes, even for AIE-active dyes, which are usually usable in mixed water–organic solvents. (iii) Other chiral frameworks, derived from not only biorelated groups (e.g., steroids and alkaloids) but also synthetic ones, will be applicable to the bent amphiphile for the construction of new chiroptically active capsules. Based on the present findings, an ongoing research project in our laboratory focuses on multicomponent host–guest systems with higher chiroptical functions.

## Data Availability

The data underlying this study are available in the published article and its Supporting Information.

## Supporting Information Available

Experimental procedures, and NMR, MS, IR, UV–vis, fluorescence, DLS, CD, CPL, X-ray, and calculation data (PDF)
