# Supplementary material for: Chiroptically Active Host–Guest Composites Using a Terpene-Based Micellar Capsule
Source: J Am Chem Soc. 2024 Aug 19;146(34):23669–73. doi: 10.1021/jacs.4c07193 (PMC11363019; doi:10.1021/jacs.4c07193)
Supplement: Supplementary file 1 — ja4c07193_si_001.pdf [file ja4c07193_si_001.pdf]

Supporting Information

Chiroptically Active Host-Guest Composites Using a Terpene-based Micellar Capsule

Yoshihisa Hashimoto, Yuya Tanaka*, Daiya Suzuki, Yoshitane Imai, and Michito Yoshizawa*

Laboratory for Chemistry and Life Science, Institute of Innovative Research, Tokyo Institute of Technology, 4259 Nagatsuta, Midori-ku, Yokohama 226-8503, Japan

Graduate School of Science and Engineering, Kindai University, 3-4-1 Kowakae, Higashi-Osaka, Osaka 577-8502, Japan

Contents

- Materials and methods, and References
- Synthesis of **1** and **1**^{E} (^{1}H, ^{13}C NMR, and MS spectra)
- Synthesis of **2** and **2**^{E} (^{1}H, ^{13}C NMR, and MS spectra)
- Synthesis of **MA** and **MA**$^{E'}$ (^{1}H, ^{13}C, DEPT 135, ^{1}H-^{1}H COSY, HSQC, HMBC, and NOESY NMR and MS spectra, optimized structure, and UV-visible spectra)
- Crystal structure and data of **MA’**
- Formation of capsule (**MA**)$_n$ (^{1}H, ^{13}C, DOSY NMR spectra, DLS charts, UV-visible, fluorescence spectra, and optimized structure)
- Formation of (**MA**)$_n$•(**TPE**)$_m$ (UV-visible, CD, ^{1}H NMR spectra, DLS chart, optimized structure, fluorescence data, and CPL spectra)
- Formation of (**MA**)$_n$•(**Cor**)$_m$ (UV-visible, ^{1}H NMR spectra, DLS chart, fluorescence data)
- Formation of (**MA**)$_n$•(**DBB**)$_m$ (UV-visible, CD, ^{1}H NMR spectra, DLS chart, optimized structure, fluorescence data, and CPL spectra)
- Construction of optimized host-guest structures

Materials and methods

NMR: Bruker AVANCE-400 (400 MHz) and AVANCE III HD 500 (500 MHz), ESI-TOF MS: Bruker micrOTOF II, UV-visible: JASCO V-670DS, Fluorescence: Hitachi F-7000, Absolute PL quantum yield: Hamamatsu C9920-02G with an integration sphere, Fluorescence lifetime: Hamamatsu C7700-ABS-N, FT-IR: SHIMADZU IRSpirit-T, DLS: Wyatt Technology DynaPro NanoStar, XRD: Rigaku XtaLAB Synergy-DW, CD: JASCO J-820, CPL: JASCO CPL-300.

Circularly Polarized Luminescence (CPL): All CPL spectra were measured in H_2O using a quartz cell (10 mm path length) at room temperature. The data were accumulated 2 or 4 times under a detector voltage of 700 or 750 V. The instrument used a scattering angle of 0° from the excitation of unpolarized, monochromated incident light with a bandwidth of 10 nm, in addition to an excitation wavelength of 402 nm and an emitted light bandwidth of 10 nm. Luminescent dissymmetry factors (g_{lum}) were calculated by JASCO Spectra Manager software.

Molecular mechanics calculation (geometry optimization): Forcite module, BIOVIA Materials Studio 2020, version 20.1.0.5 (Dassault Systèmes Co.). DFT calculation: Gaussian 16 program (Rev. C.01) package

Solvents and reagents were purchased from TCI Co., Ltd., FUJIFILM Wako Chemical Co., Kanto Chemical Co., Inc., Sigma-Aldrich Co., and Cambridge Isotope Laboratories, Inc. Mg turnings were used after the treatment with 1.2 M HCl aq., CH_3OH, and THF. (–)-Menthyl chloride and (+)-menthyl chloride were synthesized from the corresponding menthols according to ref. S1.

References

[S1] Kuhwald, C.; Kirschning, A. *Org. Lett.* **2021**, *23*, 4300–4304.

[S2] Ho, C.-Y.; Chan, C.-W.; He, L. *Angew. Chem. Int. Ed.* **2015**, *54*, 4512–4516.

[S3] Kondo, K.; Akita, M.; Nakagawa, T.; Matsuo, Y.; Yoshizawa, M. *Chem. Eur. J.* **2015**, *21*, 12741–12746.

Synthesis of 1 and 1^E YH370, 527

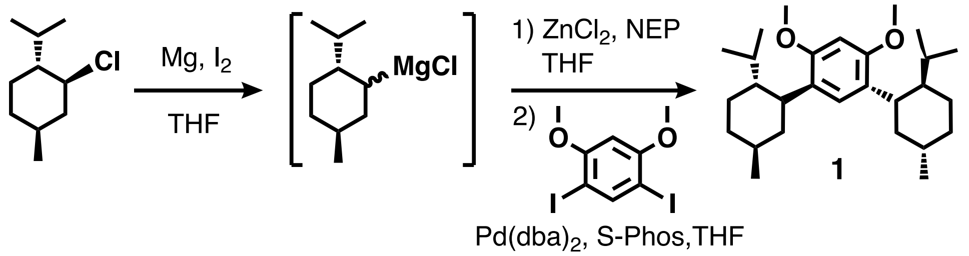

Activated Mg turnings (122 mg, 5.00 mmol), I_2 (30.1 mg, 0.118 mmol), and dry THF (8.0 mL) were added to a 50 mL 2-necked glass flask filled with N_2. The mixture was sonicated for 3 min and stirred at 75 °C for 10 min. (–)-Menthyl chloride (874 mg, 5.00 mmol) was added to the mixture dropwise and then stirred at 70 °C for 3 h. A dry THF solution (8.0 mL) of $ZnCl_2$ (817 mg, 6.00 mmol) and 1-ethyl-2-pyrrolidone (NEP, 0.75 mL, 6.63 mmol) was added to the solution dropwise at r.t. and the resultant mixture was stirred for 5 min.[S2] 1,5-Diiodo-2,4-dimethoxybenzene (488 mg, 1.25 mmol), $Pd(dba)_2$ (58.0 mg, 0.101 mmol), S-Phos (41.1 mg, 0.100 mmol), and dry THF (8.0 mL) were added to a 2-necked 20 mL glass flask filled with N_2. After sonication for 1 min, the mixture was added to the 50 mL flask dropwise. The resultant solution was stirred at 70 °C for 3 h and at r.t. overnight. After the addition of saturated NH_4Cl aq. (20 mL) at r.t., the mixture was extracted with Et_2O (3 × 10 mL). The combined organic phase was washed with H_2O (10 mL), dried over brine (10 mL) and Na_2SO_4, filtrated, and concentrated under reduced pressure. The crude product was purified by column chromatography (hexane/ethyl acetate = 5:1) and GPC ($CHCl_3$) to afford **1** (200.2 mg, 0.484 mmol, 39%) as a colorless liquid. The same procedure using (+)-menthyl chloride (874 mg, 5.00 mmol) and 1,5-diiodo-2,4-dimethoxybenzene (490 mg, 1.25 mmol) afforded **1**^{E} (210 mg, 0.507 mmol, 41%) as a colorless liquid.

Compound **1**: ^{1}H NMR (500 MHz, $CDCl_3$, r.t.): δ 0.61-0.68 (m, 6H), 0.80-0.82 (m, 6H), 0.89 (d, J = 6.5 Hz, 6H), 0.94-1.04 (m, 4H), 1.17-1.19 (m, 2H), 1.42-1.50 (m, 6H), 1.73-1.82 (m, 6H), 1.98 (br, 0.5H), 2.30 (br, 0.5H), 3.00 (br, 2H), 3.81 (s, 6H), 6.41 (s, 1H), 6.81-6.89 (s × 2, 1H). ^{13}C NMR (125 MHz, CD_3OD, r.t.): δ 15.2-16.0 (CH_3 × 3), 21.6 (CH_3), 22.6 (CH_3), 24.8-25.3 (CH_2 × 3), 27.5-28.4 (CH × 2), 33.3-33.7 (CH × 2), 35.5 (CH_2), 37.0-37.3 (CH × 2), 44.1-44.9 (CH_2 × 3), 47.2-47.5 (CH × 2), 55.2-55.8 (CH_3 × 3), 95.1-96.2 (CH × 2), 125.4-130.1 (5 peaks, CH & C_q), 155.1-156.4 (C_q × 3). FT-IR (ATR, cm^{-1}): 2949, 1612, 1586, 1502, 1454, 1368, 1288, 1198, 1172, 1111, 1040, 815. HR MS (ESI, CH_3OH): m/z Calcd. For $C_{28}H_{46}O_2Na$ 437.3390 $[M + Na]^+$, Found 437.3391.

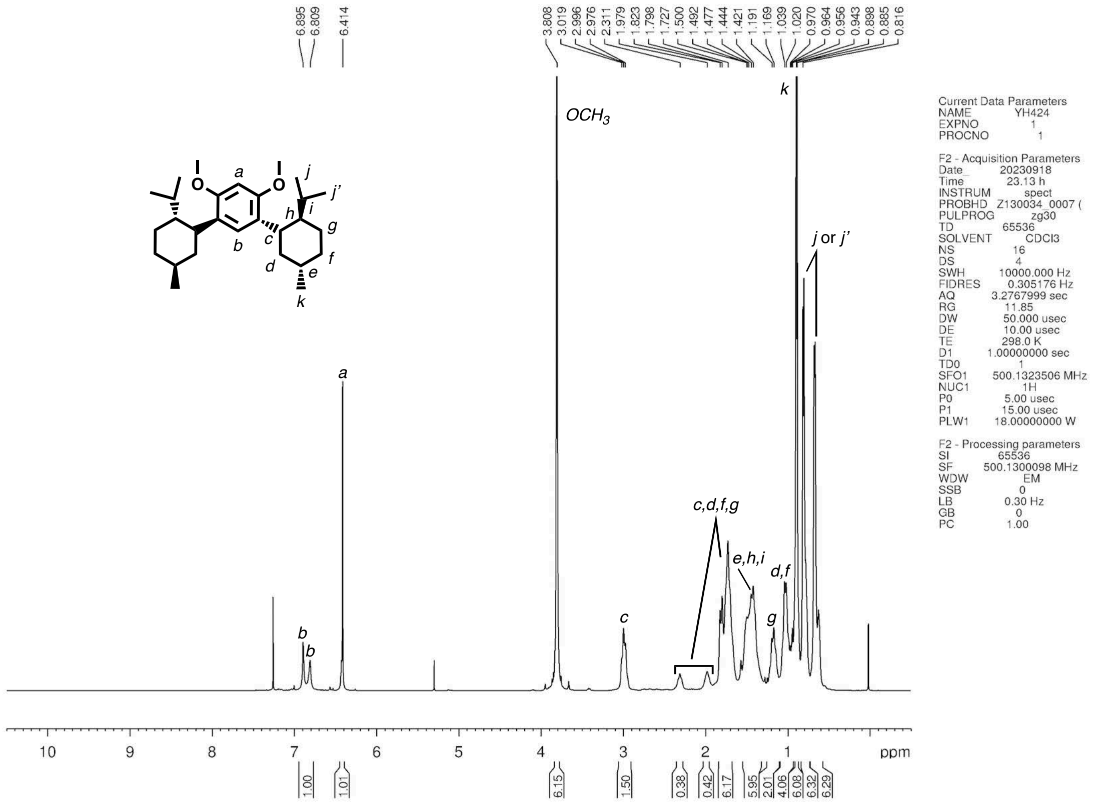

Figure S1. ^{1}H NMR spectrum (500 MHz, $CDCl_3$, r.t.) of **1**.

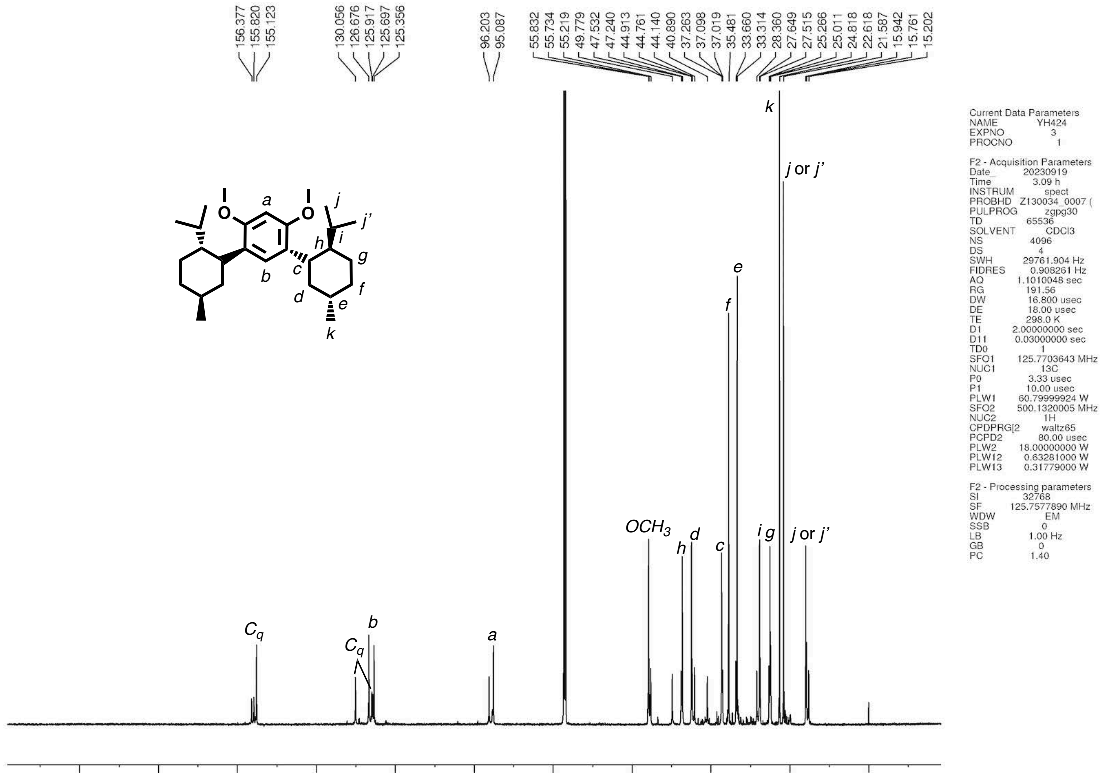

Figure S2. ^{13}C NMR spectrum (125 MHz, $CDCl_3$, r.t.) of **1**.

Analysis Info

Analysis Name	D:\Data\akita\22hashimoto\ME-OMe 231101\ME-OMe.d	Acquisition Date	2023/11/01 14:23:43
Method	esi_posi_low.m	Operator	BDAL@DE
Sample Name	ME-OMe	Instrument	micrOTOF 213750.10321
Comment			

Acquisition Parameter

Source Type	ESI	Ion Polarity	Positive	Set Nebulizer	0.3 Bar
Focus	Not active			Set Dry Heater	180 • ‹C
Scan Begin	50 m/z	Set Capillary	4500 V	Set Dry Gas	4.0 l/min
Scan End	1000 m/z	Set End Plate Offset	-500 V	Set Divert Valve	Waste

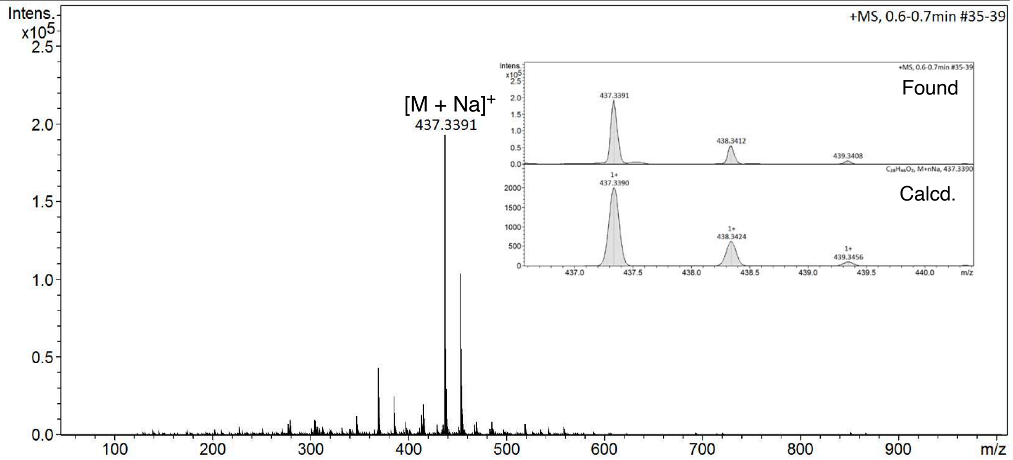

Figure S3. HR MS spectrum (CH_3OH) of **1**.

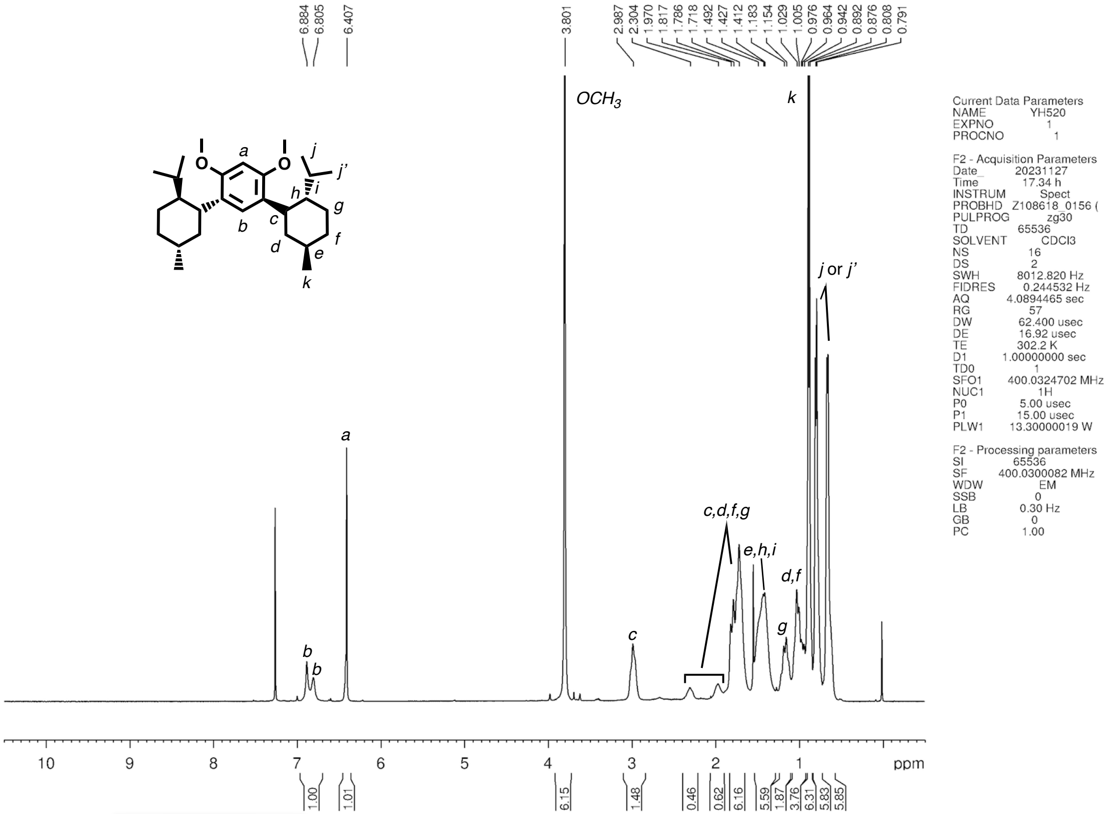

Figure S4. ^{1}H NMR spectrum (400 MHz, $CDCl_3$, r.t.) of **1^E^**.

Synthesis of 2 and 2^E YH426, 535

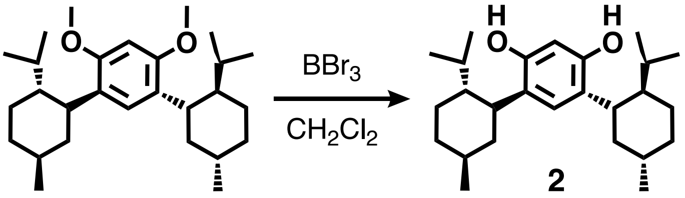

Compound **1** (250 mg, 0.603 mmol) and dry CH_2Cl_2 were added to a 2-necked 50 mL glass flask filled with N_2. A CH_2Cl_2 solution of BBr_3 (1.0 M, 2.41 mL, 2.41 mmol) was added dropwise to this flask at 0 ºC under N_2. The mixture was stirred at 0 ºC to r.t. for 3 h. The reaction was quenched with H_2O (10 mL). The two phases were separated and the aqueous phase was extracted with CH_2Cl_2 (10 mL). The combined organic phase was dried over Na_2SO_4, filtrated, and concentrated under reduced pressure to afford **2** (214.2 mg, 0.554 mmol, 92%) as a white solid. The same procedure using $\mathbf{1^E}$ (571 mg, 1.38 mmol) afforded $\mathbf{2^E}$ (476 mg, 1.23 mmol, 89%) as a white solid.

Compound **2**: ^{1}H NMR (400 MHz, CD_3OD, r.t.): δ 0.67 (d, J = 6.4 Hz, 6H), 0.81-0.83 (m, 6H), 0.89 (d, J = 6.4 Hz, 6H), 0.93-1.05 (m, 4H), 1.15-1.18 (m, 2H), 1.48 (br, 6H), 1.73-1.82 (m, 6H), 2.35 & 2.70 (br, 2H), 4.46 (s, 2H), 6.19-6.24 (br, 1H), 6.76-6.82 (s × 2, 1H). ^{13}C NMR (100 MHz, CD_3OD, r.t.): δ 15.2-16.0 (2 peaks, CH_3), 21.5 (CH_3), 22.5 (CH_3), 25.1 (CH_2), 27.6-28.4 (CH × 2), 33.3 (CH_2), 35.4 (CH), 38.0 (CH), 44.9 (CH_2), 47.1 (CH), 102.5-103.8 (CH × 2), 123.8-125.9 (CH × 3), 130.6 (C_q), 150.8-151.9 (C_q × 3). FT-IR (ATR, cm^{-1}): 2922, 1616, 1512, 1456, 1368, 1196, 1104, 999, 939, 760, 735. HR MS (ESI, CH_3OH): m/z Calcd. For $C_{26}H_{42}O_2Na$ 409.3077 $[M + Na]^+$, Found 409.3078.

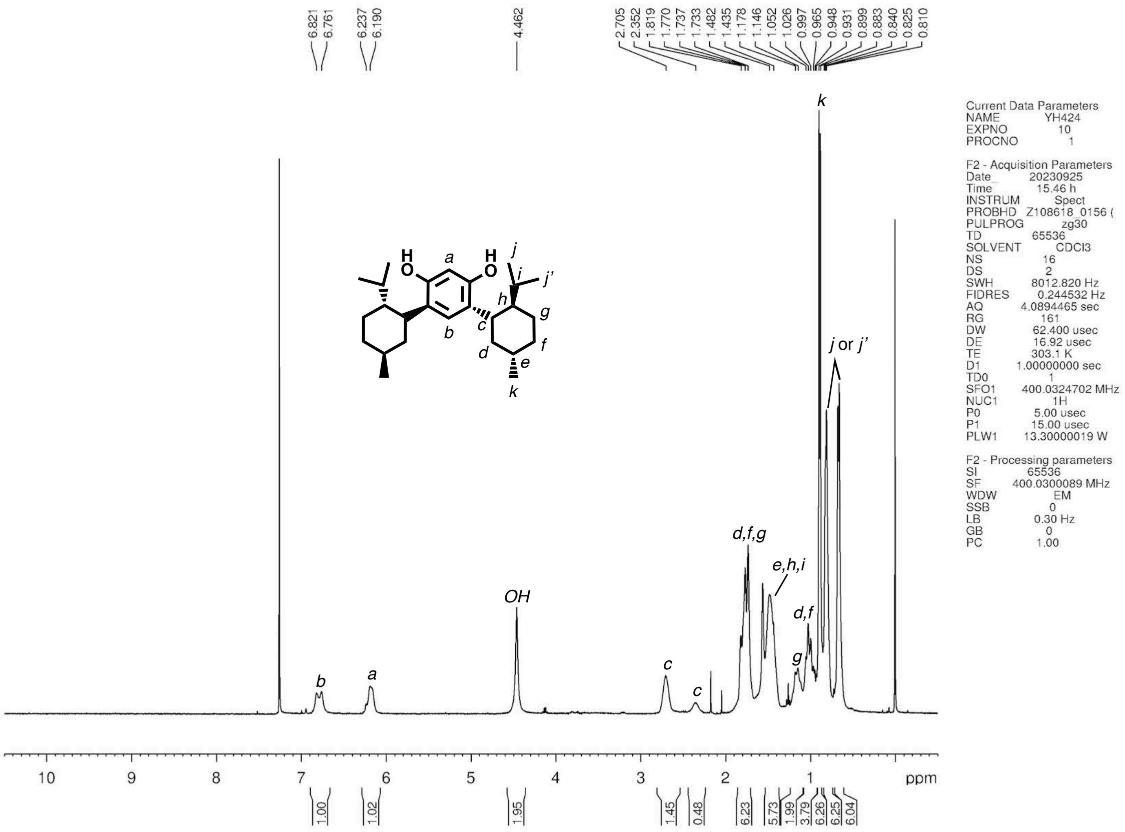

Figure S5. ^{1}H NMR spectrum (400 MHz, $CDCl_3$, r.t.) of **2**.

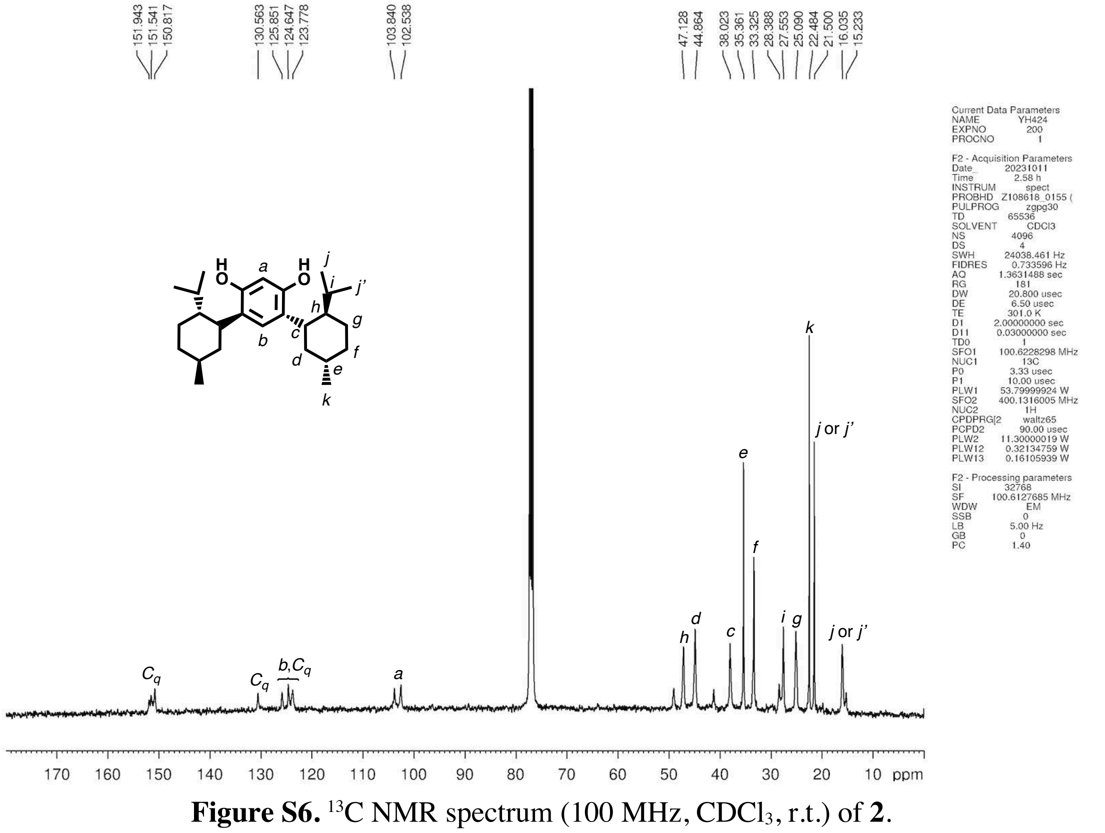

Figure S6. ^{13}C NMR spectrum (100 MHz, $CDCl_3$, r.t.) of **2**.

Analysis Info

Analysis Name	D:\Data\akita\22hashimoto\ME-OMe 231101\ME-OMe.d	Acquisition Date	2023/11/01 14:23:43
Method	esi_posi_low.m	Operator	BDAL@DE
Sample Name	ME-OMe	Instrument	micrOTOF 213750.10321
Comment			

Acquisition Parameter

Source Type	ESI	Ion Polarity	Positive	Set Nebulizer	0.3 Bar
Focus	Not active			Set Dry Heater	180 • ‹C
Scan Begin	50 m/z	Set Capillary	4500 V	Set Dry Gas	4.0 l/min
Scan End	1000 m/z	Set End Plate Offset	-500 V	Set Divert Valve	Waste

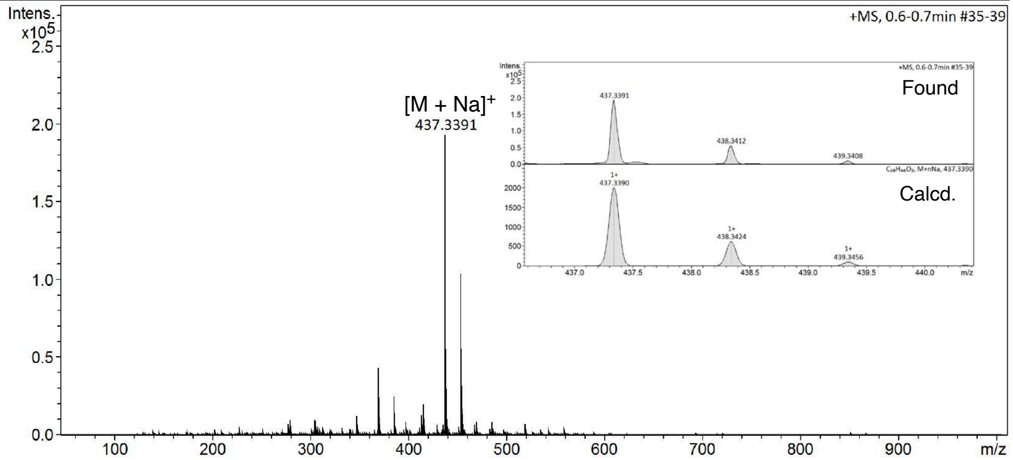

Figure S7. HR MS spectrum (CH_3OH) of **2**.

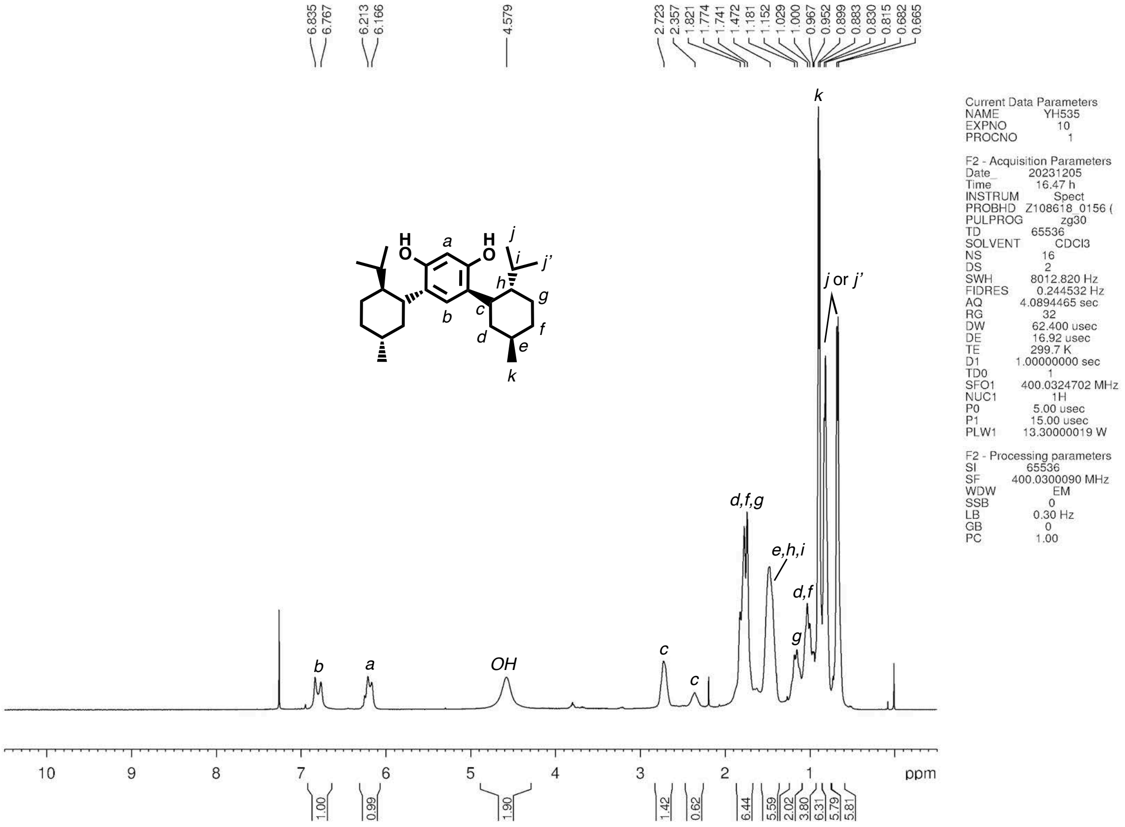

Figure S8. ^{1}H NMR spectrum (400 MHz, $CDCl_3$, r.t.) of $\mathbf{2^E}$.

Synthesis of MA and MA^E YH420, 425, 543, 567

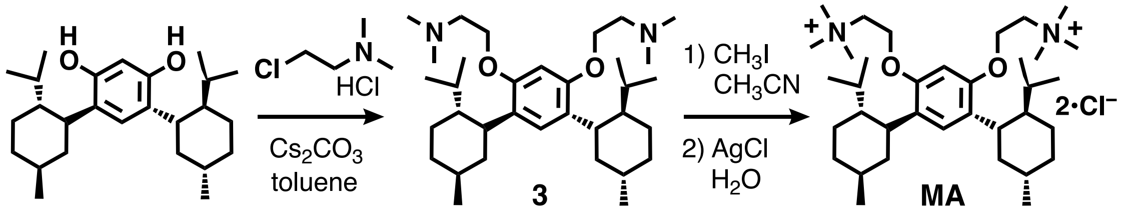

Compound **2** (166.2 mg, 0.429 mmol), Cs_2CO_3 (4.19 g, 12.9 mmol), and dry toluene (10 mL) were added to a 2-necked 30 mL glass flask. The mixture was stirred at 80 ºC for 30 min. 2-Chloro-*N,N*-dimethylethanamine hydrochloride (494 mg, 3.43 mmol) was added to the reaction mixture at r.t. The resultant mixture was stirred at 130 ºC overnight. The resultant mixture was filtered at r.t., and then concentrated under reduced pressure. The crude product was extracted with EtOAc (3 × 10 mL). The combined organic phase was dried over Na_2SO_4, filtrated, and concentrated under reduced pressure to afford **3** (crude; 159 mg, 0.300 mmol) as a brown solid. Compound **3** (159 mg, 0.300 mmol), CH_3I (0.30 mL, 4.85 mmol), and CH_3CN (3.0 mL) were added to a 50 mL glass flask. The mixture was stirred at r.t. overnight. The resultant mixture was concentrated under reduced pressure. The crude product was washed with Et_2O to afford **4** (221 mg, 0.273 mmol, 64% based on **2** (two steps)) as a brown solid. Compound **4** (200 mg, 0.246 mmol) and AgCl (141 mg, 0.984 mmol) were stirred in H_2O (3.0 mL) at 80 ºC overnight. After the addition of CH_3OH (5.0 mL), the resultant solution was centrifugated, filtered, and concentrated under reduced pressure. The crude product was washed with acetone to afford **MA** (177 mg, 0.274 mmol, 89%) as a white solid. The same procedure using **3^E** (200 mg, 0.246 mmol) afforded **MA^E** (99.5 mg, 0.158 mmol, 64%) as a white solid.

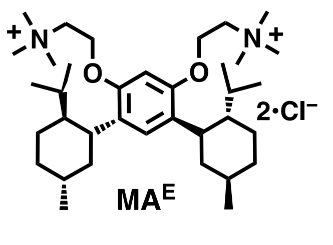

Compound **3** (crude): ^{1}H NMR (400 MHz, $CDCl_3$, r.t.): δ 0.64 (d, J = 6.5 Hz, 6H), 0.78 (d, J = 6.8 Hz, 6H), 0.86-1.00 (m, 10H), 1.13-1.16 (m, 2H), 1.41-1.43 (m, 6H), 1.68-1.79 (m, 8H), 2.34 (s, 12H), 2.72-2.75 (m, 4H), 4.00-4.02 (m, 4H), 6.39 (s, 1H), 6.82 (s × 2, 1H). ^{13}C NMR (100 MHz, $CDCl_3$, r.t.): δ 15.2-16.1 (3 peaks, CH_3), 21.5 (CH_3), 22.6 (CH_3), 25.1 (CH_2), 27.6-28.3 (CH × 2), 33.4 (CH), 35.5 (CH_2), 37.5 (CH), 45.0 (CH_2), 46.1 (CH_3), 47.1-47.3 (CH × 2), 58.6 (CH_2), 66.8-67.3 (CH_2 × 2), 97.3-98.0 (CH), 125.4-130.1 (5 peaks, CH & C_q), 154.3-155.4 (C_q × 3).

MA: ^{1}H NMR (400 MHz, CD_3OD, r.t.): δ 0.67 (d, J = 6.9 Hz, 6H), 0.83 (d, J = 6.9 Hz, 6H), 0.91 (d, J = 6.5 Hz, 6H), 0.95-1.11 (m, 4H), 1.18-1.26 (m, 2H), 1.46-1.55 (m, 6H), 1.69-1.72 (m, 2H), 1.79-1.87 (m, 4H), 2.95-3.00 (m, 2H), 3.35 (s, 18H), 3.97 (t, J = 4.4 Hz, 4H), 4.60 (br, 4H), 6.78-6.86 (s × 2, 1H), 6.99 (s, 1H). ^{13}C NMR (100 MHz, CD_3OD, r.t.): δ 15.2 (CH_3), 20.4 (CH_3), 21.5 (CH_3), 24.8 (CH_2), 27.4 (CH), 33.3 (CH), 35.1 (CH_2), 38.0 (CH), 45.2 (CH_2), 46.7 (CH), 53.5 (CH_3), 62.7 (CH_2), 65.6 (CH_2), 98.2 (CH), 125.6 (CH), 127.5 (C_q), 135.4 (C_q). FT-IR (ATR, cm^{-1}): 3375, 2992, 1611, 1481, 1368, 1282, 1199, 1111, 1054, 963, 876, 598, 519. ESI-TOF MS (CH_3OH): m/z 593.6 [M – Cl^-]$^+$, 279.3 [M – 2•Cl^-]$^{2+}$.

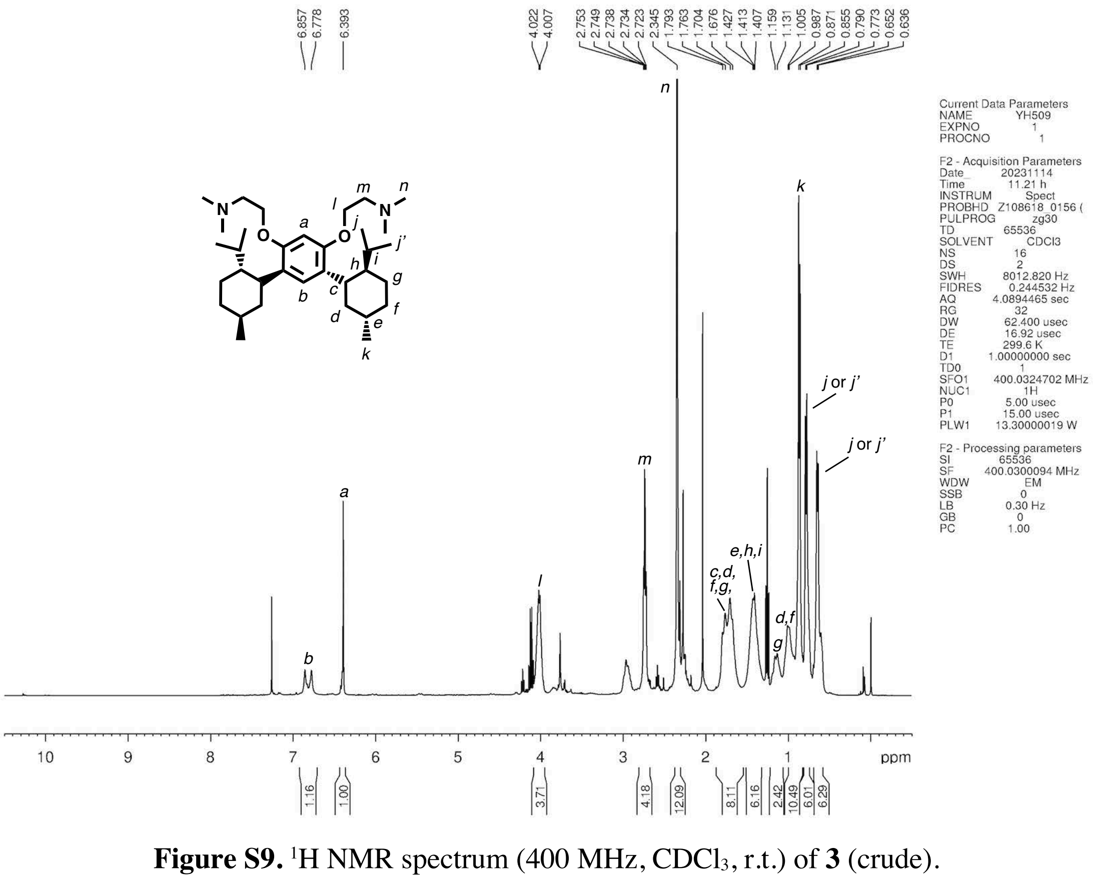

Figure S9. ^{1}H NMR spectrum (400 MHz, $CDCl_3$, r.t.) of **3** (crude).

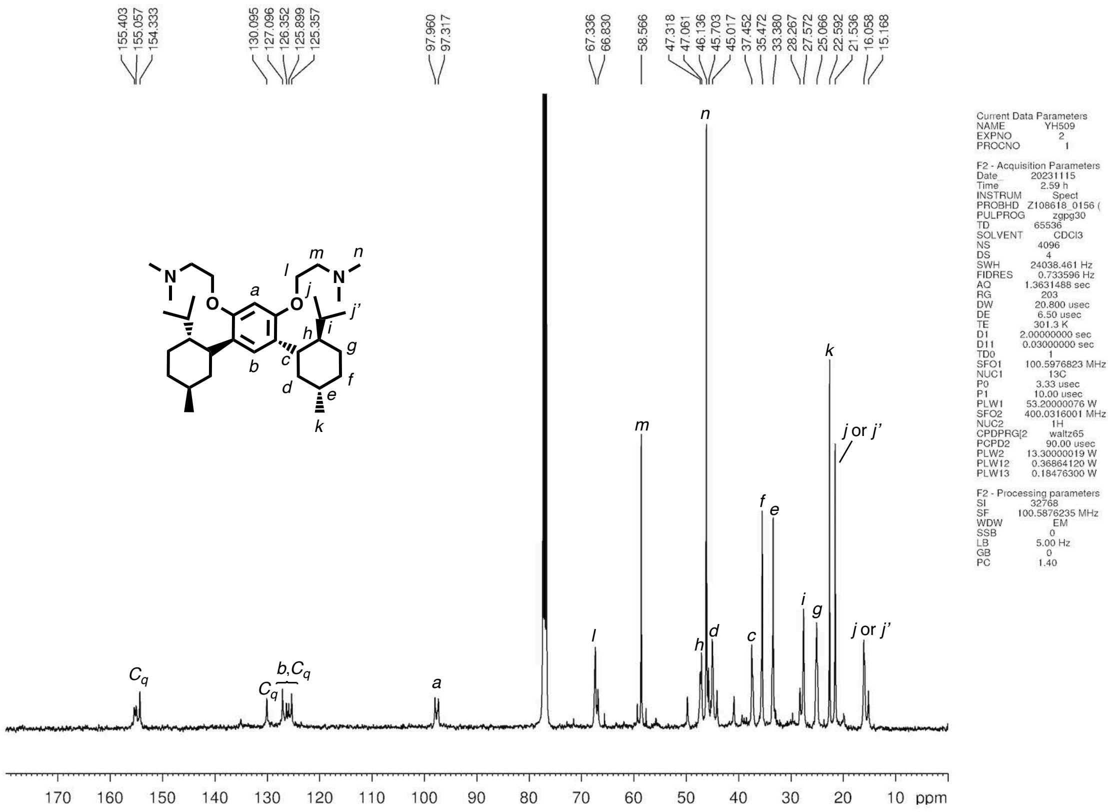

Figure S10. ^{13}C NMR spectrum (100 MHz, $CDCl_3$, r.t.) of **3** (crude).

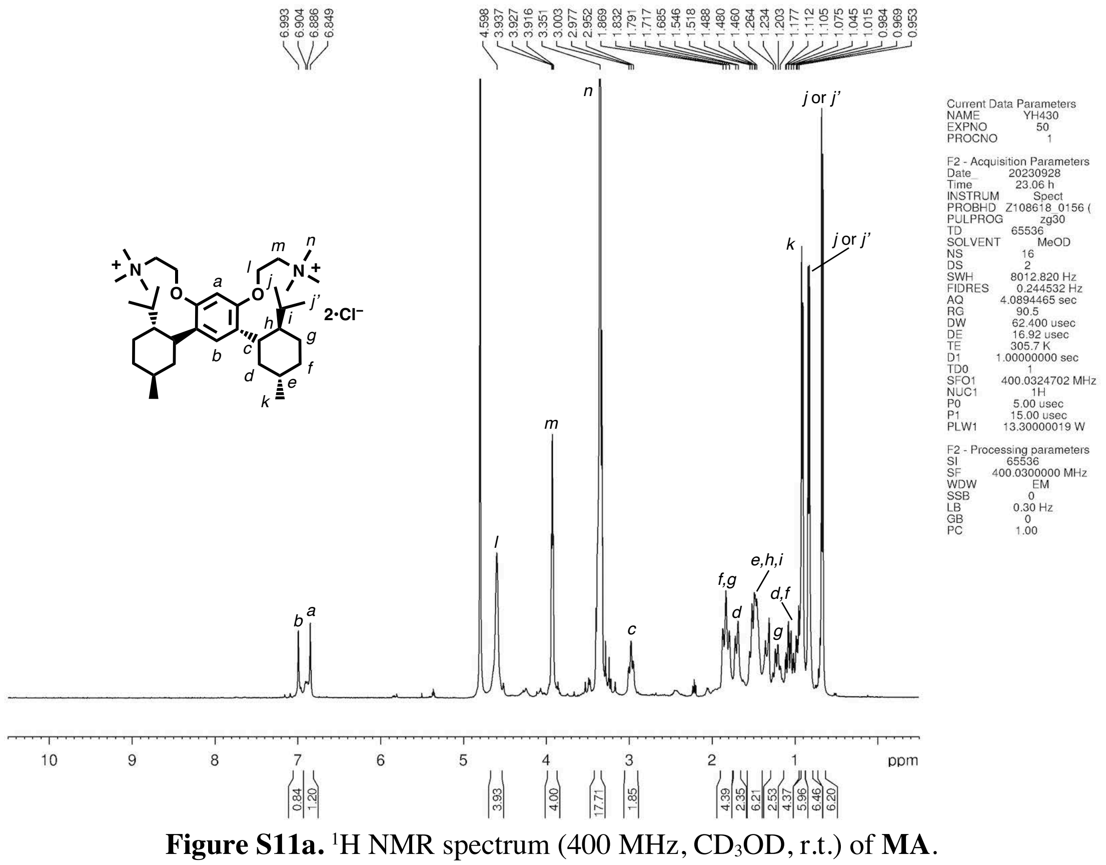

Figure S11a. ^{1}H NMR spectrum (400 MHz, CD_3OD, r.t.) of **MA**.

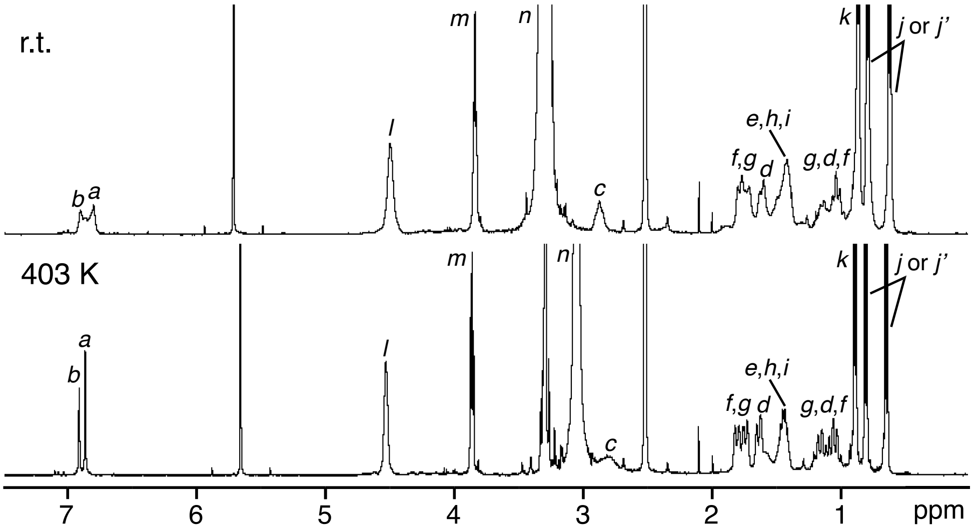

Figure S11b. ^{1}H NMR spectra (400 MHz, DMSO-d_6) of **MA** at r.t. (top) and 403 K (bottom).

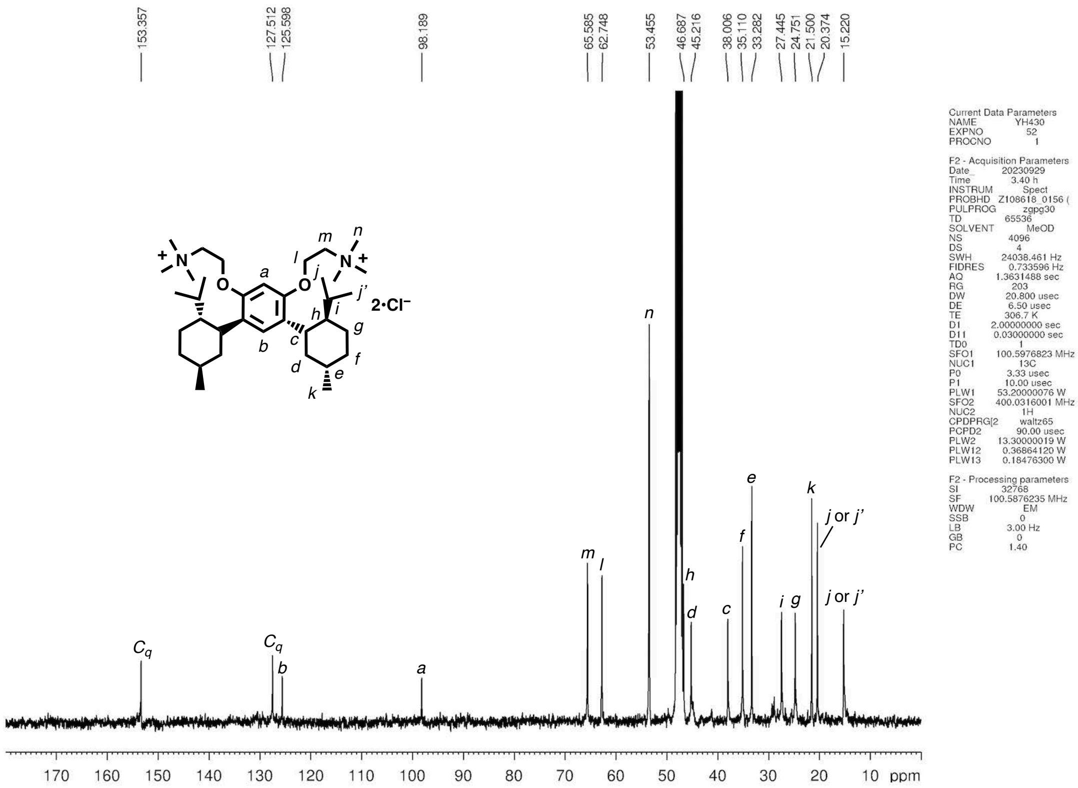

Figure S12a. ^{13}C NMR spectrum (100 MHz, CD_3OD, r.t.) of **MA**.

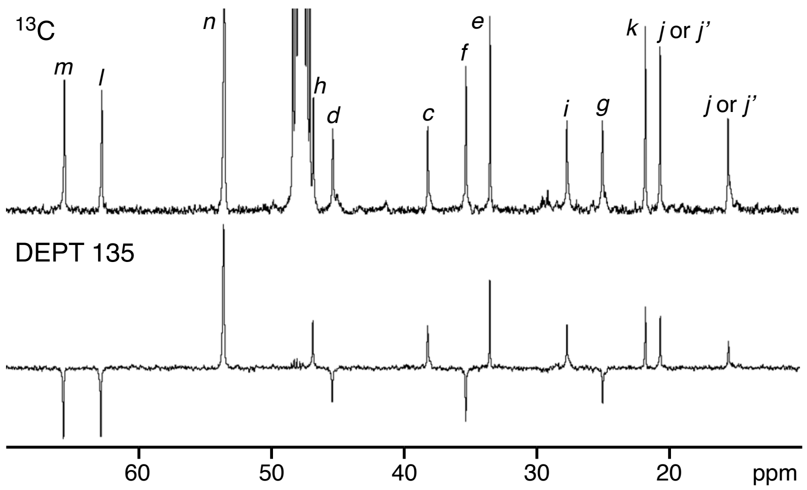

Figure S12b. ^{13}C and DEPT 135 NMR spectra (100 MHz, CD_3OD, r.t.) of **MA** (10-70 ppm).

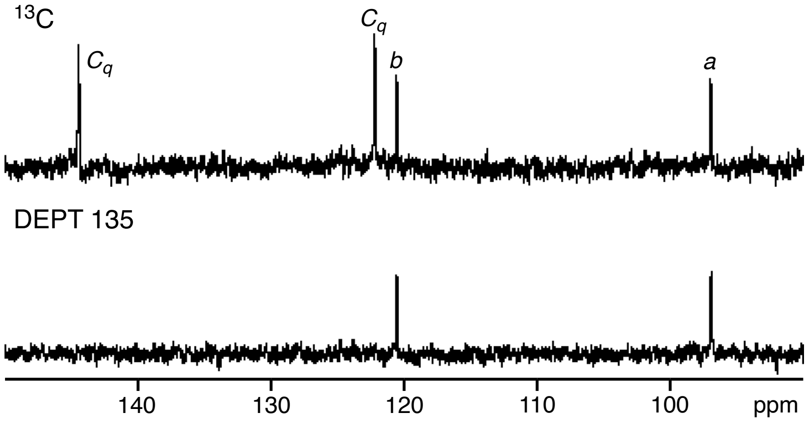

Figure S12c. ^{13}C and DEPT 135 NMR spectra (100 MHz, CD_3OD, r.t.) of **MA** (90-150 ppm).

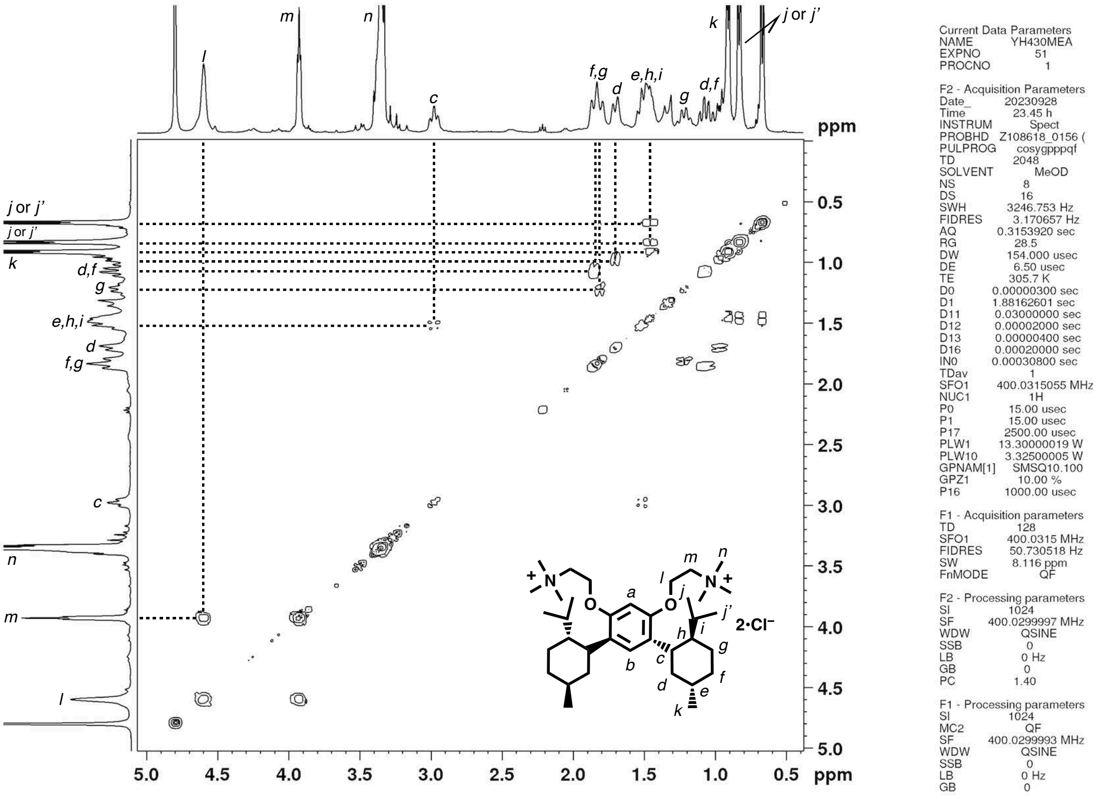

Figure S13. ^{1}H-^{1}H COSY spectrum (400 MHz, CD_3OD, r.t.) of **MA**.

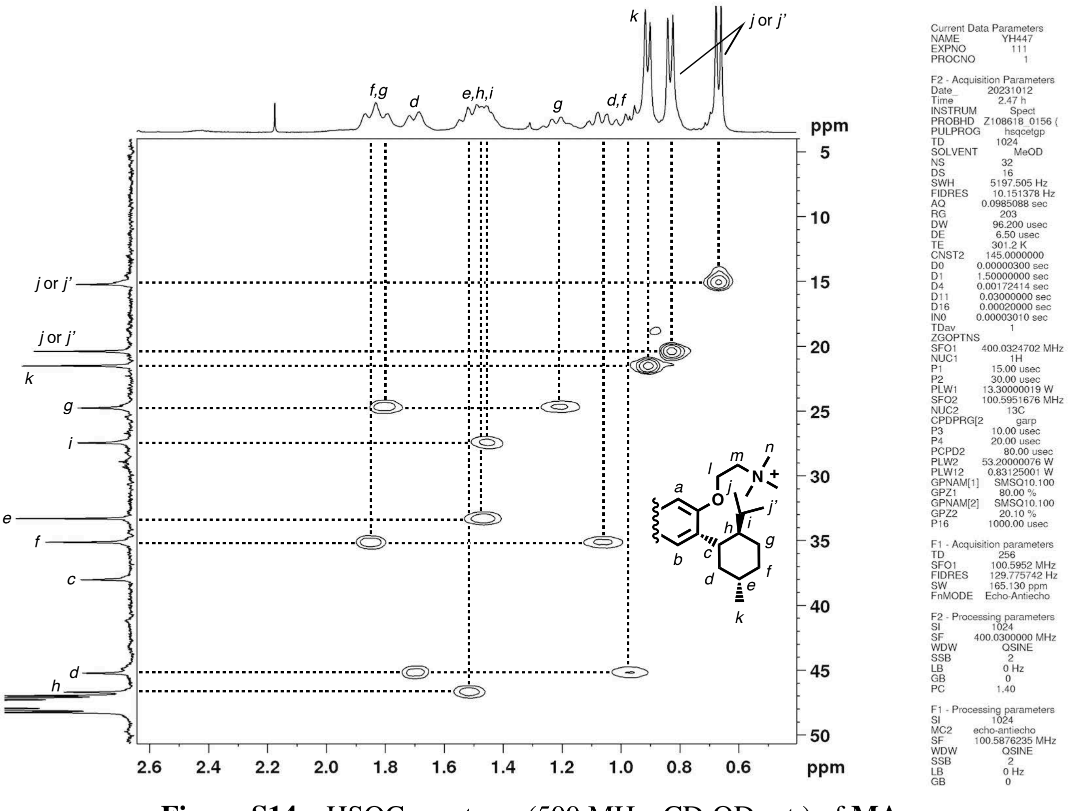

Figure S14a. HSQC spectrum (500 MHz, CD_3OD, r.t.) of **MA**.

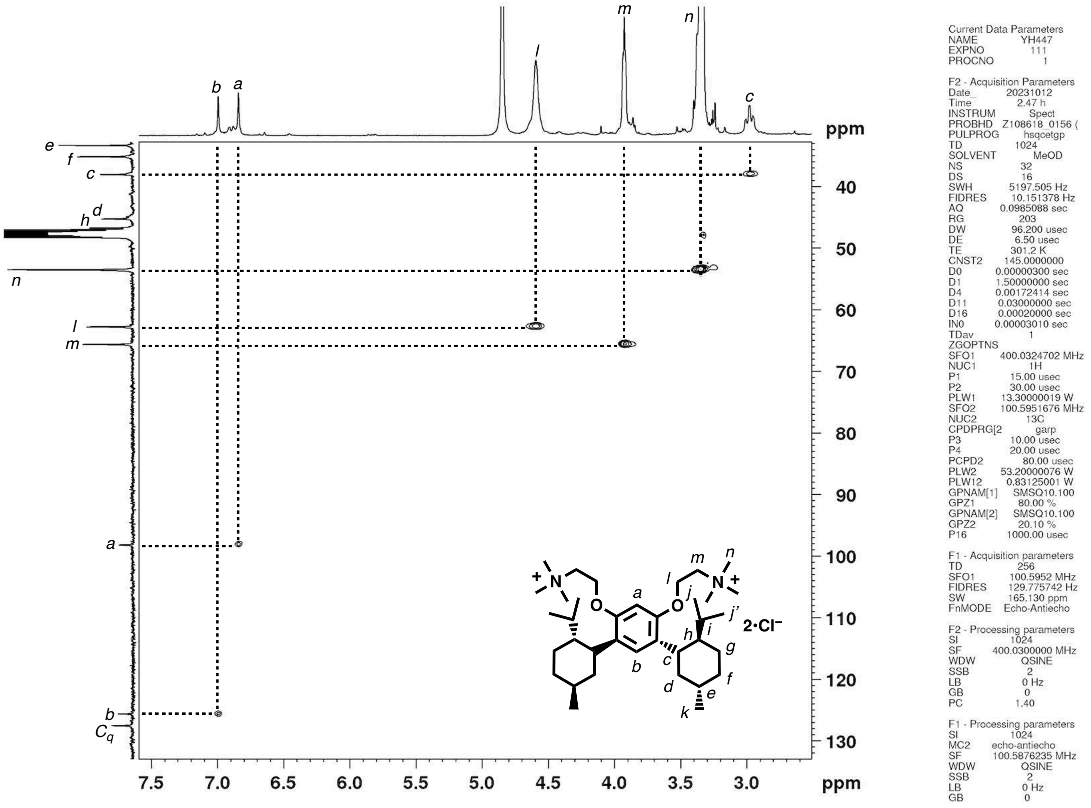

Figure S14b. HSQC spectrum (500 MHz, CD_3OD, r.t.) of **MA**.

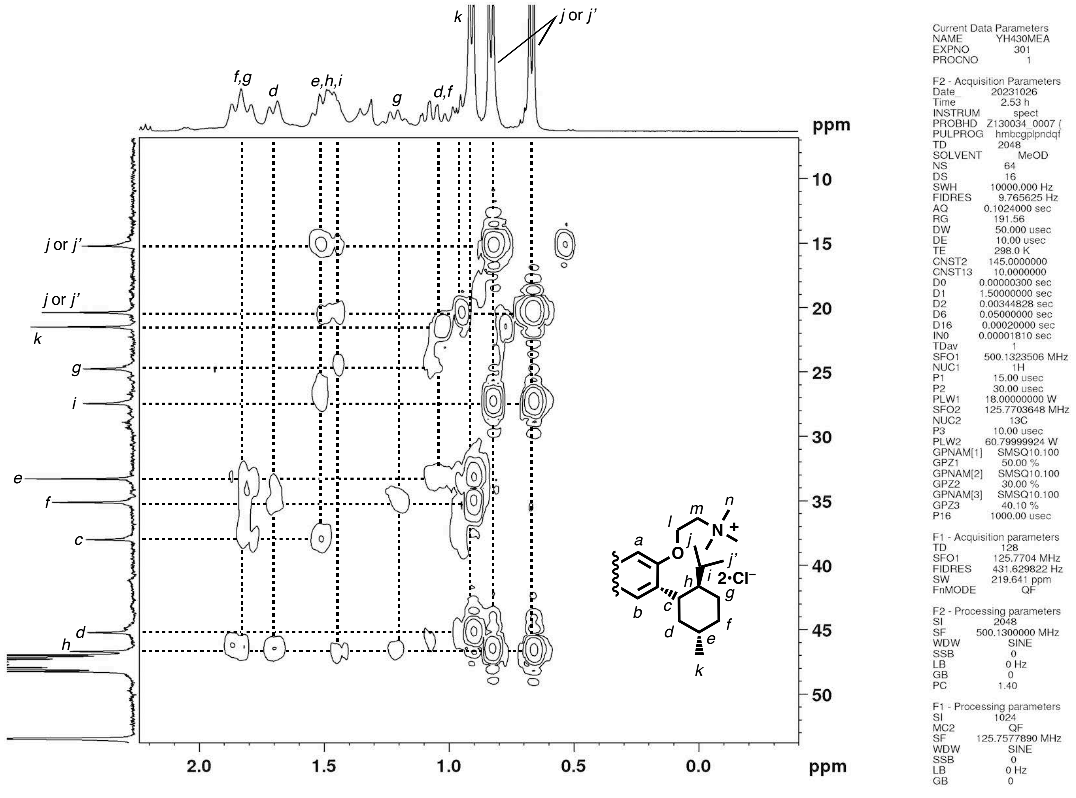

Figure S15a. HMBC spectrum (125 MHz, CD_3OD, r.t.) of **MA**.

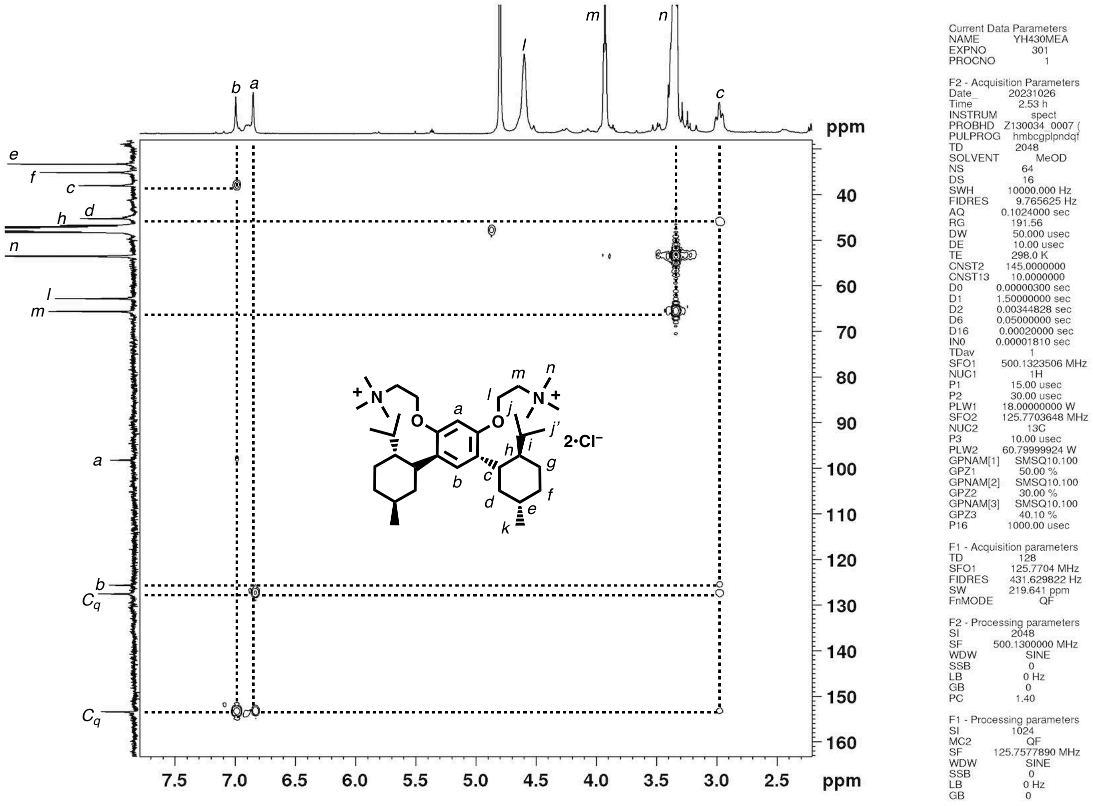

Figure S15b. HMBC spectrum (125 MHz, CD_3OD, r.t.) of **MA**.

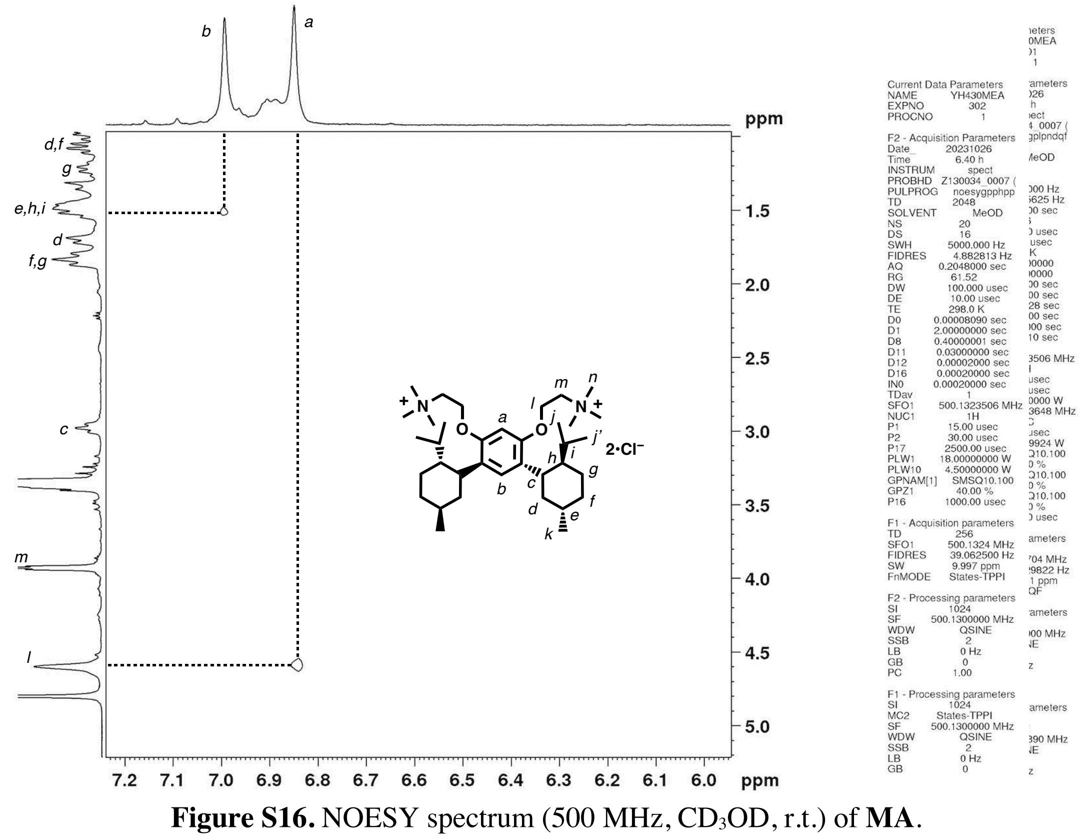

Figure S16. NOESY spectrum (500 MHz, CD_3OD, r.t.) of **MA**.

Analysis Info

Analysis Name	D:\Data\akita\22hashimoto\MEA 231017\Acq000001.d	Acquisition Date	2023/10/17 17:41:51
Method	esi_posi_low.m	Operator	BDAL@DE
Sample Name	MEA 231017	Instrument	micrOTOF 213750.10321
Comment			

Acquisition Parameter

Source Type	ESI	Ion Polarity	Positive	Set Nebulizer	0.3 Bar
Focus	Not active			Set Dry Heater	40 • ‹C
Scan Begin	50 m/z	Set Capillary	4500 V	Set Dry Gas	4.0 l/min
Scan End	1000 m/z	Set End Plate Offset	-500 V	Set Divert Valve	Waste

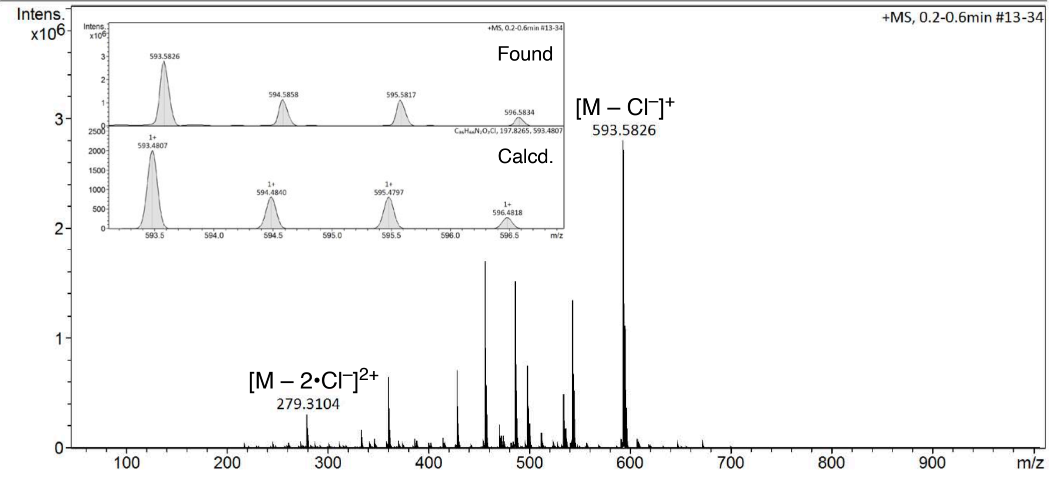

Figure S17. ESI-TOF MS spectrum (CH_3OH) of **MA**.

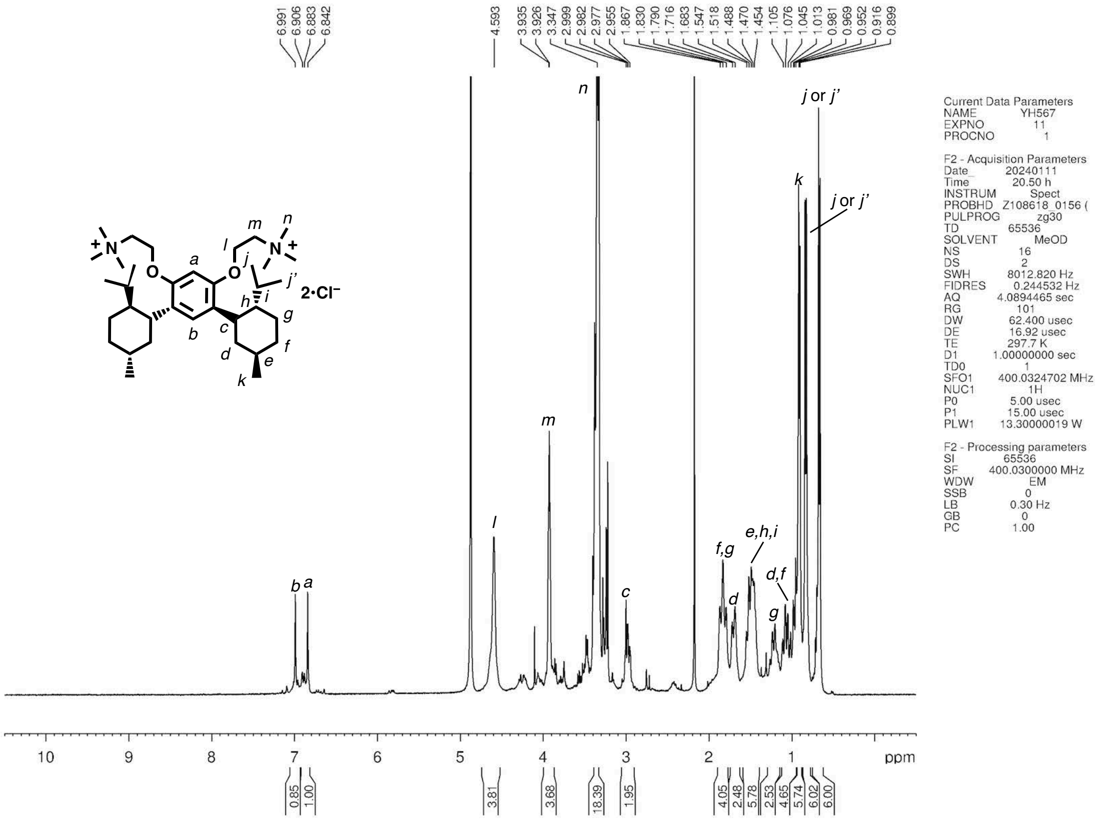

Figure S18a. ^{1}H NMR spectrum (400 MHz, CD_3OD, r.t.) of **MA^E**.

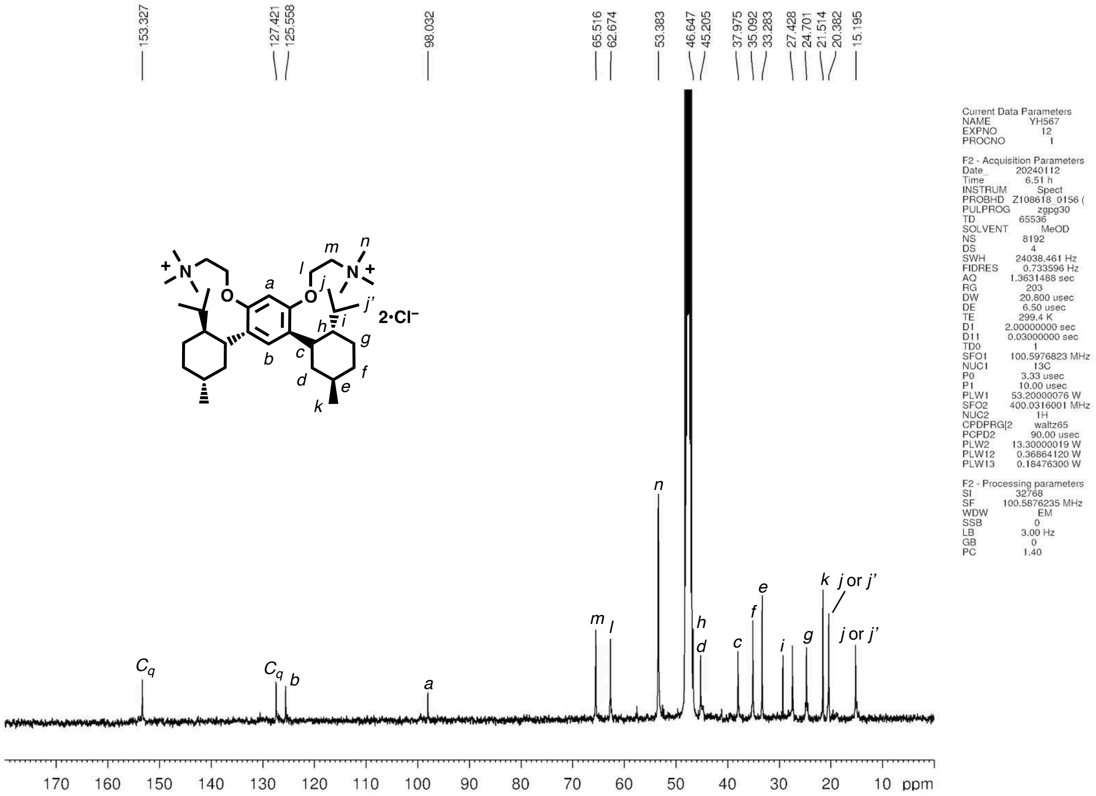

Figure S18b. ^{13}C NMR spectrum (100 MHz, CD_3OD, r.t.) of **MA^E**.

Analysis Info

Analysis Name	D:\Data\akita\22hashimoto\MAR 240126\MAR 240126000001.d	Acquisition Date	2024/01/26 12:19:26
Method	esi_posi_low.m	Operator	BDAL@DE
Sample Name	MAR 240126	Instrument	micrOTOF 213750.10321
Comment			

Acquisition Parameter

Source Type	ESI	Ion Polarity	Positive	Set Nebulizer	0.3 Bar
Focus	Not active			Set Dry Heater	180 • ‹C
Scan Begin	50 m/z	Set Capillary	4500 V	Set Dry Gas	4.0 l/min
Scan End	1000 m/z	Set End Plate Offset	-500 V	Set Divert Valve	Waste

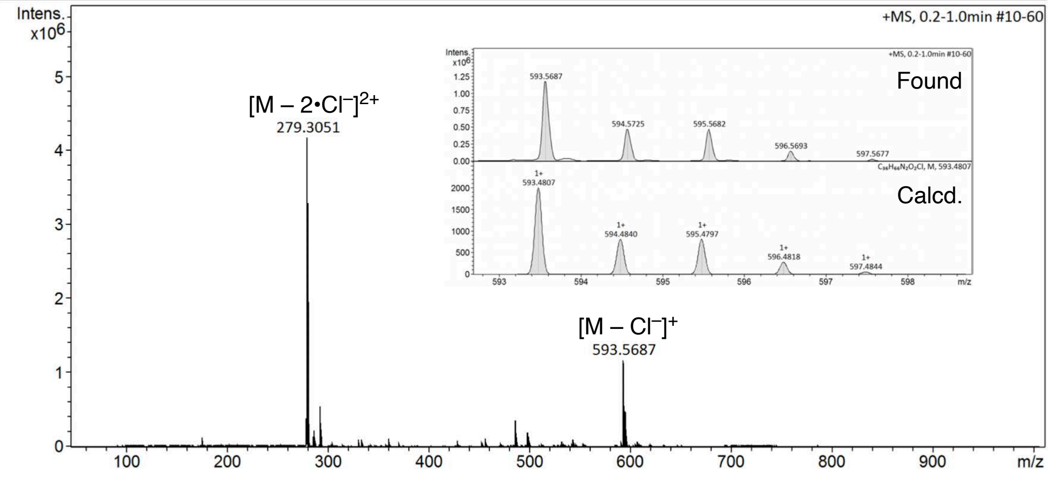

Figure S18c. ESI-TOF MS spectrum (CH_3OH) of **MA^E**.

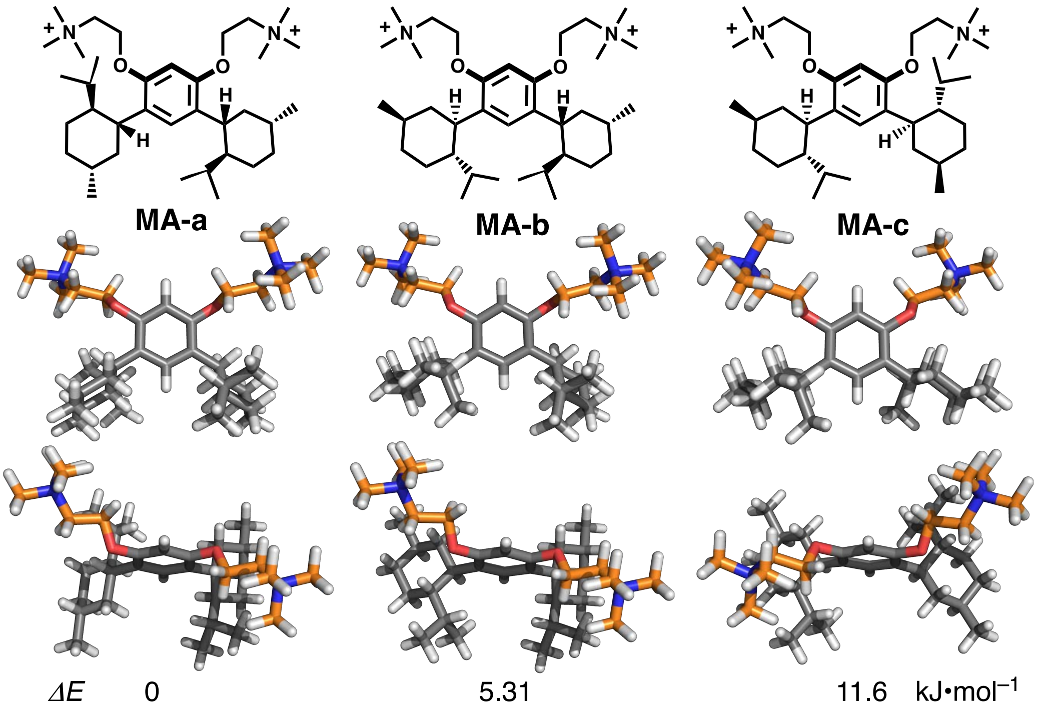

Figure S19. Optimized structures and relative energies of rotational isomers **MA-a**, **MA-b**, and **MA-c** (DFT, B3LPY/6-31G(d,p) level).

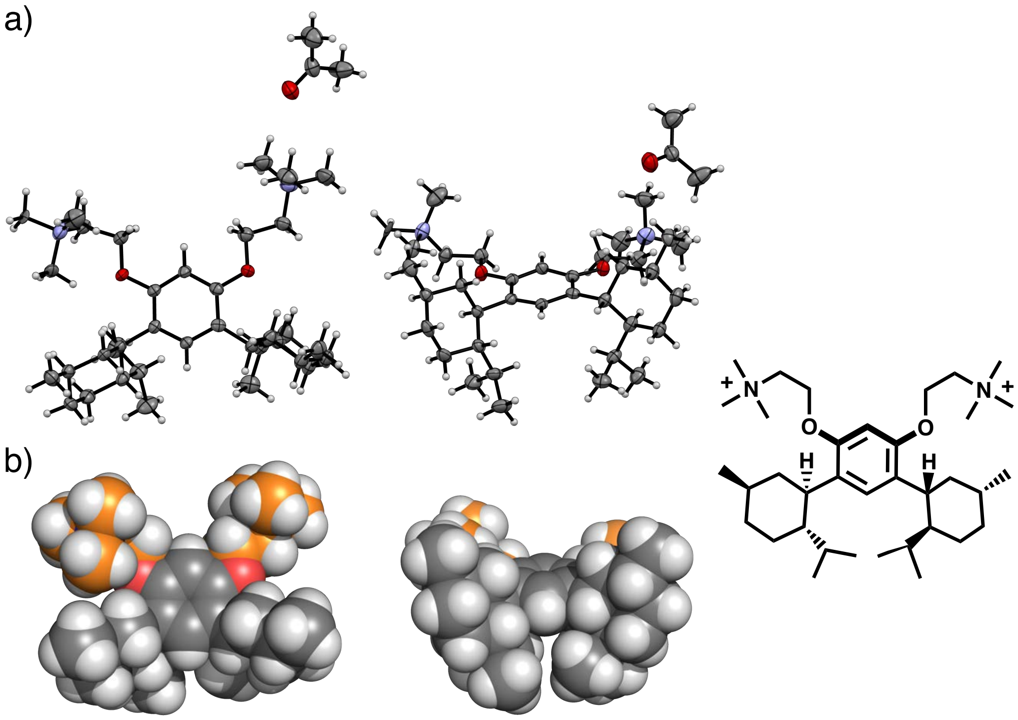

Figure S20. Selected crystal structure of **MA'** (front and bottom views): a) ORTEP drawing (the thermal ellipsoids are drawn at 50% probability) and b) space-filling model (solvents and counter anions are omitted for clarity).

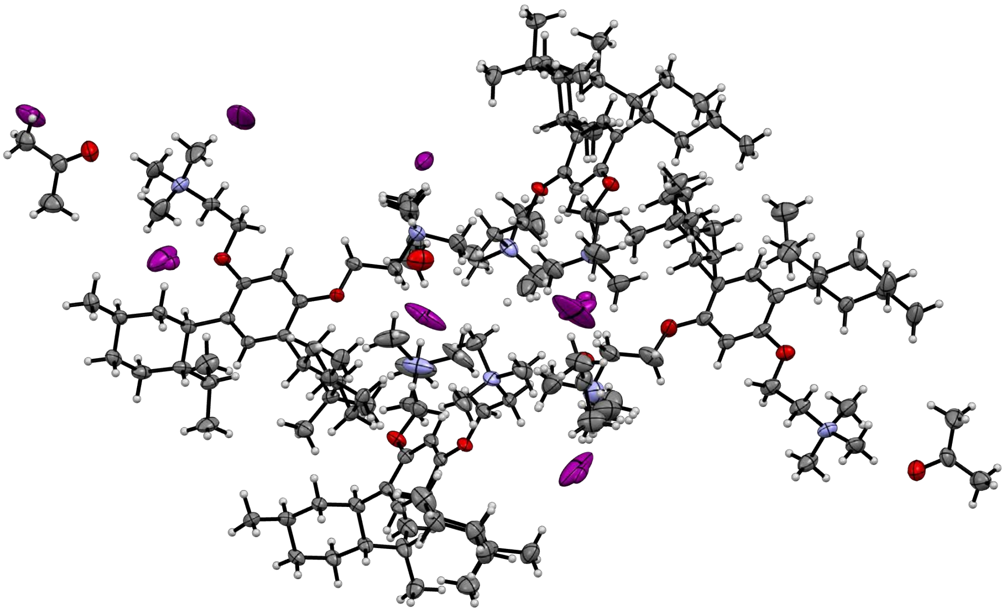

Figure S21. ORTEP drawings of **MA'**. The thermal ellipsoids are drawn at 50% probability.

Table S1. Crystal data and structure refinement for **MA'**.

Identification code	MEA-I3_auto
Empirical formula	$C_{78}H_{143}I_4N_4O_6$
Formula weight	1740.56
Temperature/K	93(2)
Crystal system	monoclinic
Space group	$P2_1$
a/Å	13.59920(10)
b/Å	40.1707(2)
c/Å	16.07000(10)
α/°	90
β/°	90.4440(10)
γ/°	90
Volume/Å^3	8778.61(10)
Z	4
ρ_{calc}g/cm^3	1.317
μ/mm^{-1}	11.502
F(000)	3596.0
Crystal size/mm^3	0.04 × 0.04 × 0.02
Radiation	CuKα (λ = 1.54184)
2θ range for data collection/°	4.4 to 149.904
Index ranges	−16 ≤ h ≤ 16, −46 ≤ k ≤ 50, −20 ≤ l ≤ 20
Reflections collected	82576
Independent reflections	29485 [R_{int} = 0.0442, R_{sigma} = 0.0457]
Data/restraints/parameters	29485/98/1782
Goodness-of-fit on F^2	1.023
Final R indexes [I>=2σ (I)]	R_1 = 0.0636, wR_2 = 0.1676
Final R indexes [all data]	R_1 = 0.0670, wR_2 = 0.1701
Largest diff. peak/hole / e Å^{-3}	3.21/−1.64
Flack parameter	0.000(3)

The supplementary crystallographic data (CCDC 2357518) can be obtained free of charge from the Cambridge Crystallographic Data Centre via www.ccdc.cam.ac.uk/structures.

Formation of capsule (MA)$_n$ YH445

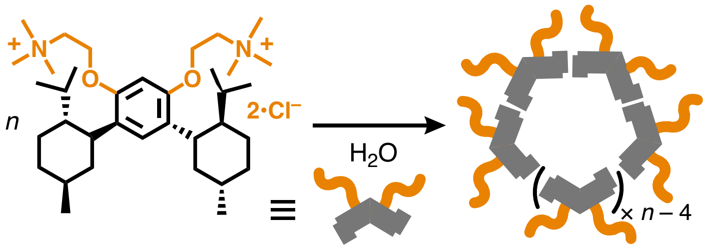

Amphiphile **MA** (15.7 mg, 25.0 μmol) was added to water (1.0 mL) and the solution was stirred at r.t. for 1 min to give a clear solution including capsule (**MA**)$_n$. The quantitative formation and structure of (**MA**)$_n$ were confirmed by ^{1}H and ^{13}C NMR, and DLS analyses. The optimized structure of capsule (**MA**)$_6$ was obtained by molecular mechanics calculation (Forcite module, Materials Studio).

^{1}H NMR (400 MHz, D_2O, r.t., 25 mM based on **MA**): δ 0.53-0.62 (m, 6H), 0.70-0.77 (m, 6H), 0.83-1.02 (m, 10H), 1.18-1.46 (m, 8H), 1.66-1.86 (m, 6H), 2.27 & 2.86 (br, 2H), 3.23 (s, 18H), 3.77 (m, 4H), 4.46 (m, 4H), 6.67-6.88 (m, 2H). ^{13}C NMR (100 MHz, D_2O, r.t., 25 mM based on **MA**): δ 15.2-16.1 (3 peaks, CH_3), 21.2-21.4 (2 peaks, CH_3), 22.3-22.6 (2 peaks, CH_3), 22.5-25.1 (3 peaks, CH_2), 27.2-28.2 (3 peaks, CH), 33.0-33.4 (2 peaks, CH), 35.3 (CH_2), 37.5-37.7 (2 peaks, CH), 44.1-45.0 (2 peaks, CH_2), 46.7 (CH), 54.0 (CH_3), 62.6-62.7 (2 peaks, CH_2), 65.6-65.8 (2 peaks, CH_2), 97.7-99.3 (2 peaks, CH), 125.3-130.0 (5 peaks, CH & C_q), 153.2-154.6 (3 peaks, C_q). DOSY NMR (500 MHz, D_2O, 12 mM based on **MA**, 25 °C): $D = 1.95 \times 10^{-10}$ m^2 s^{-1}.

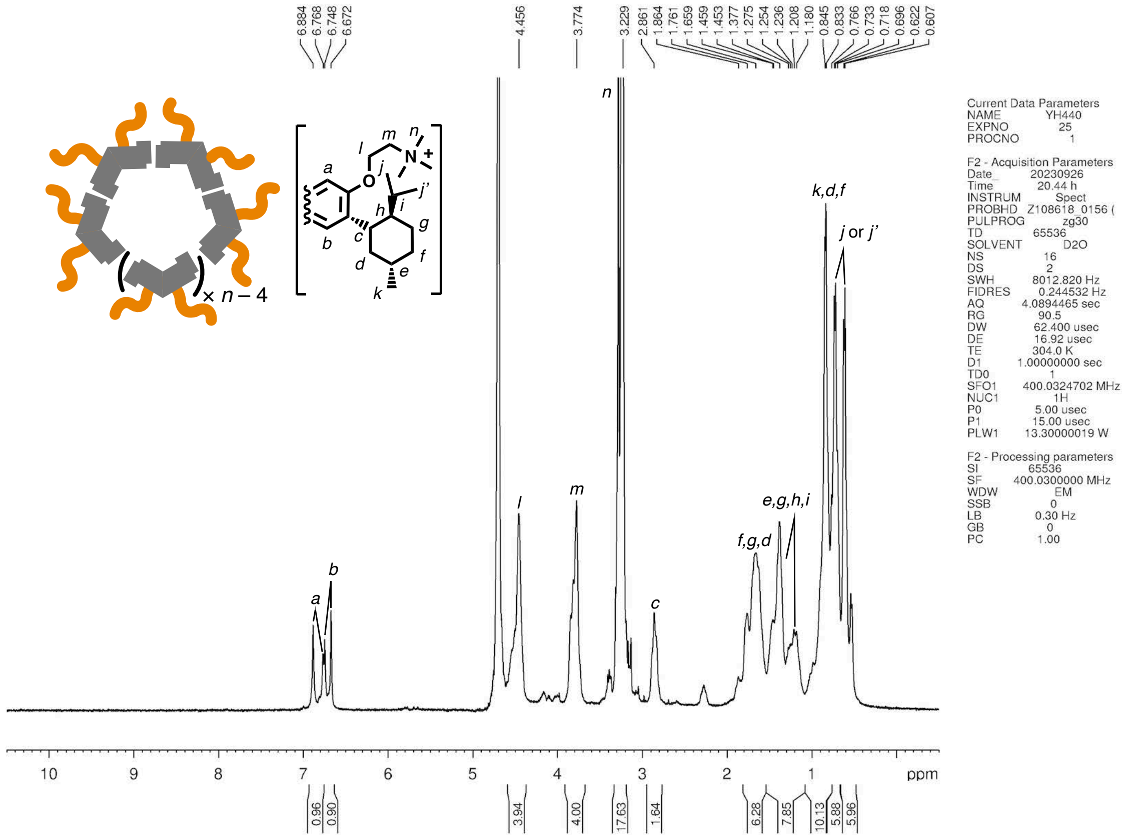

Figure S22. ^{1}H NMR spectrum (400 MHz, D_2O, r.t., 25 mM based on **MA**) of $(\mathbf{MA})_n$.

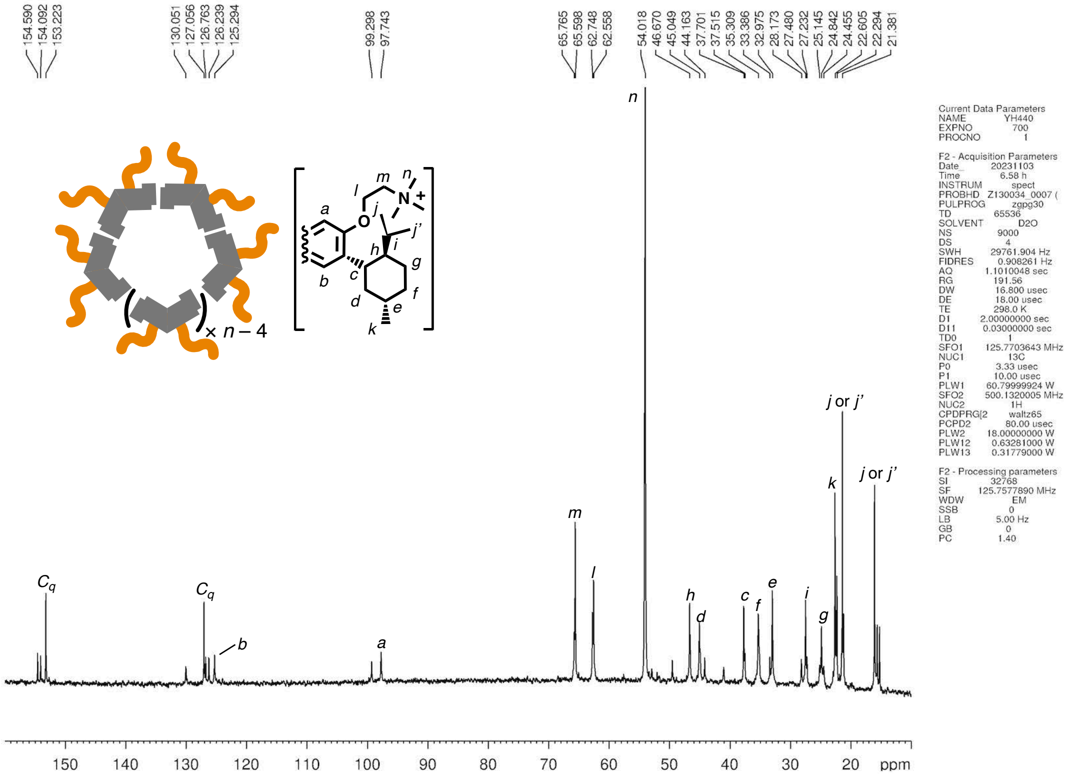

Figure S23. ^{13}C NMR spectrum (125 MHz, D_2O, r.t., 50 mM based on **MA**) of $(\mathbf{MA})_n$.

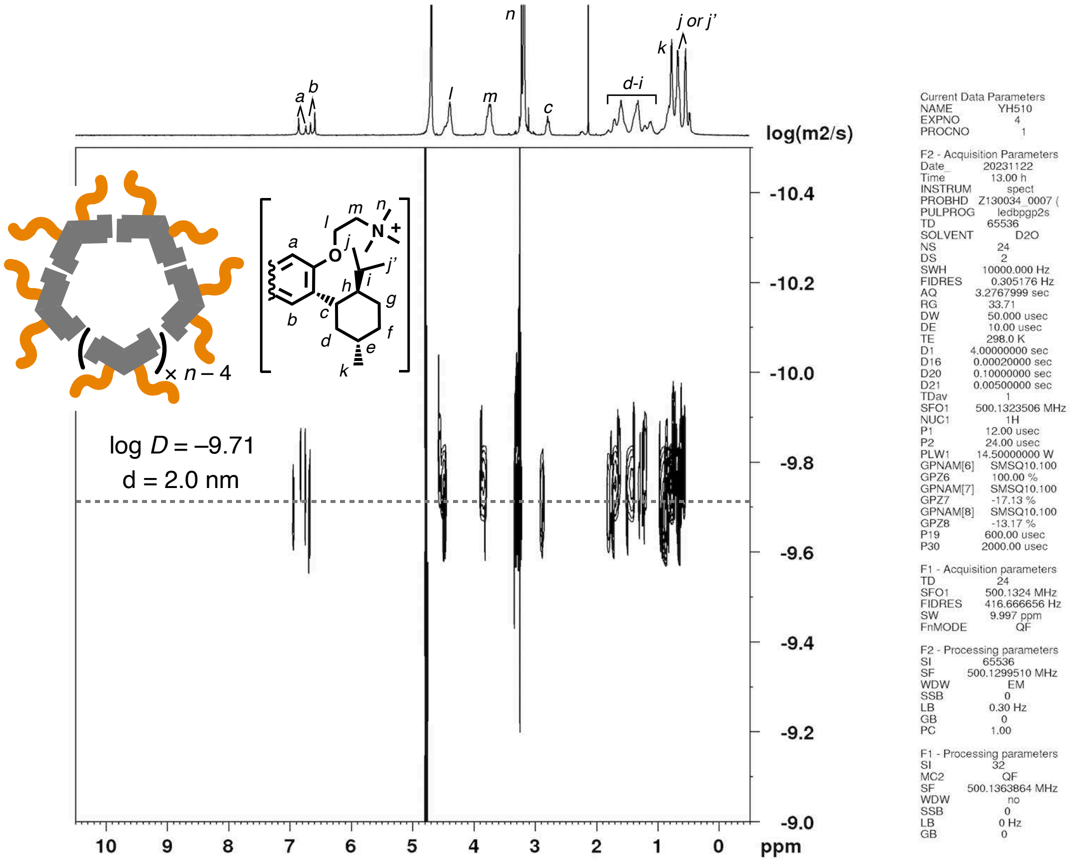

Figure S24. DOSY NMR spectrum (500 MHz, D_2O, 298 K, 12 mM based on **MA**) of (**MA**)$_n$.

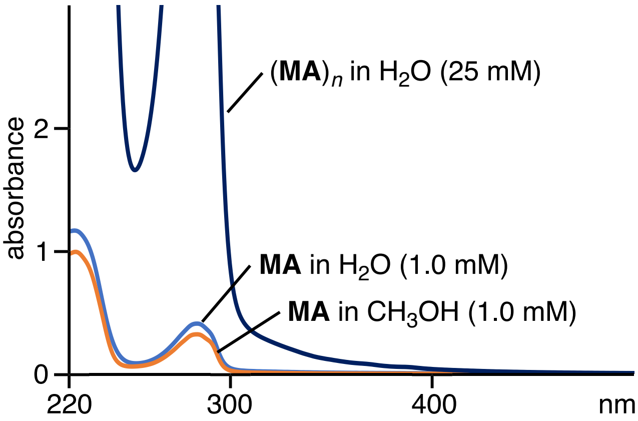

Figure S25. UV-visible spectra (r.t., 1.0 or 25 mM based on **MA**) of **MA** in H_2O and CH_3OH, and capsule (**MA**)$_n$ in H_2O.

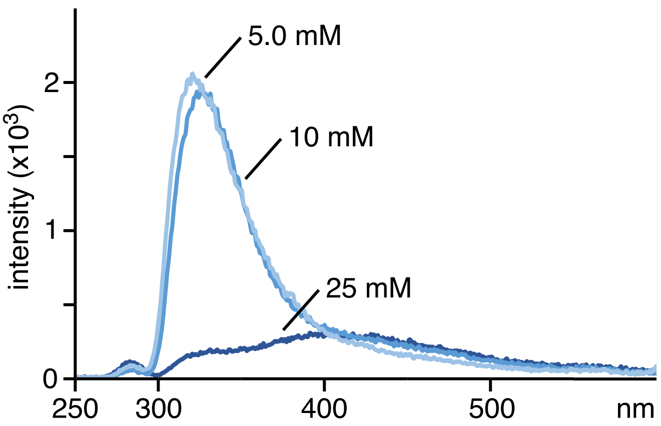

Figure S26. Concentration-dependent fluorescence spectra (r.t., 5.0-25 mM based on **MA**, λ_{ex} = 283 nm) of $(\mathbf{MA})_n$ in H_2O.

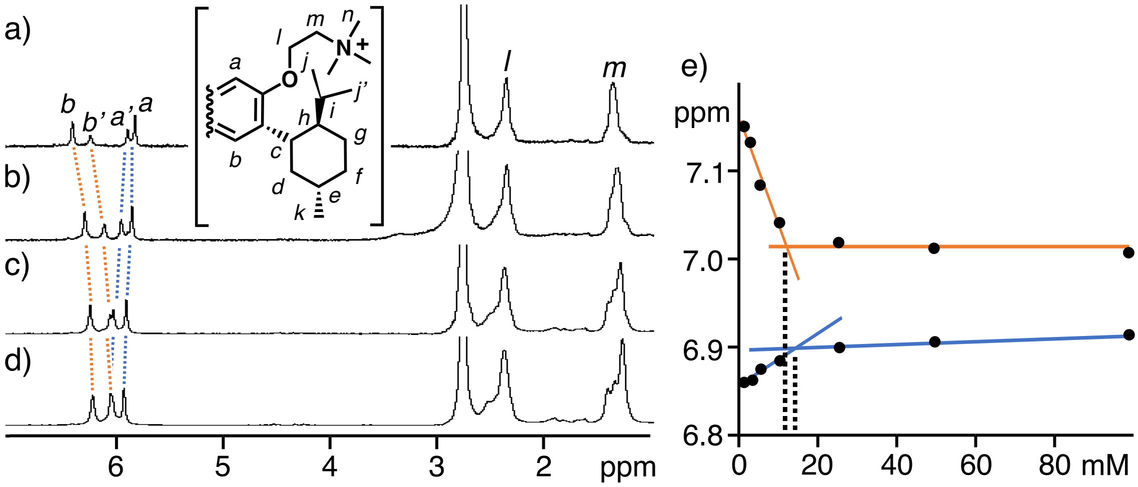

Figure S27. Concentration-dependent ^{1}H NMR spectra (400 MHz, D_2O, r.t.) of $(\mathbf{MA})_n$: a) 5.0, b) 10, c) 25, and d) 50 mM based on **MA**. e) Plot of the chemical shift of H_a (blue line) and H_b (orange line) versus the concentration of **MA**.

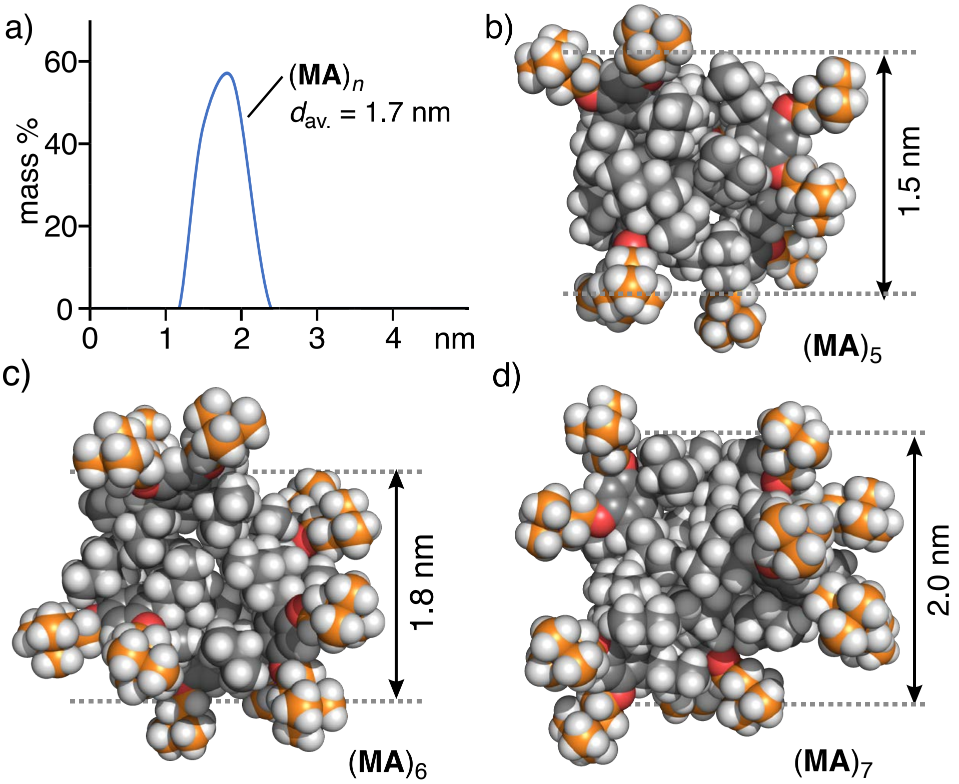

Figure S28. a) DLS chart (H_2O, r.t., 25 mM based on **MA**) of capsule (**MA**)$_n$. The optimized structure (MM calculation) of b) (**MA**)$_5$, c) (**MA**)$_6$, and d) (**MA**)$_7$.

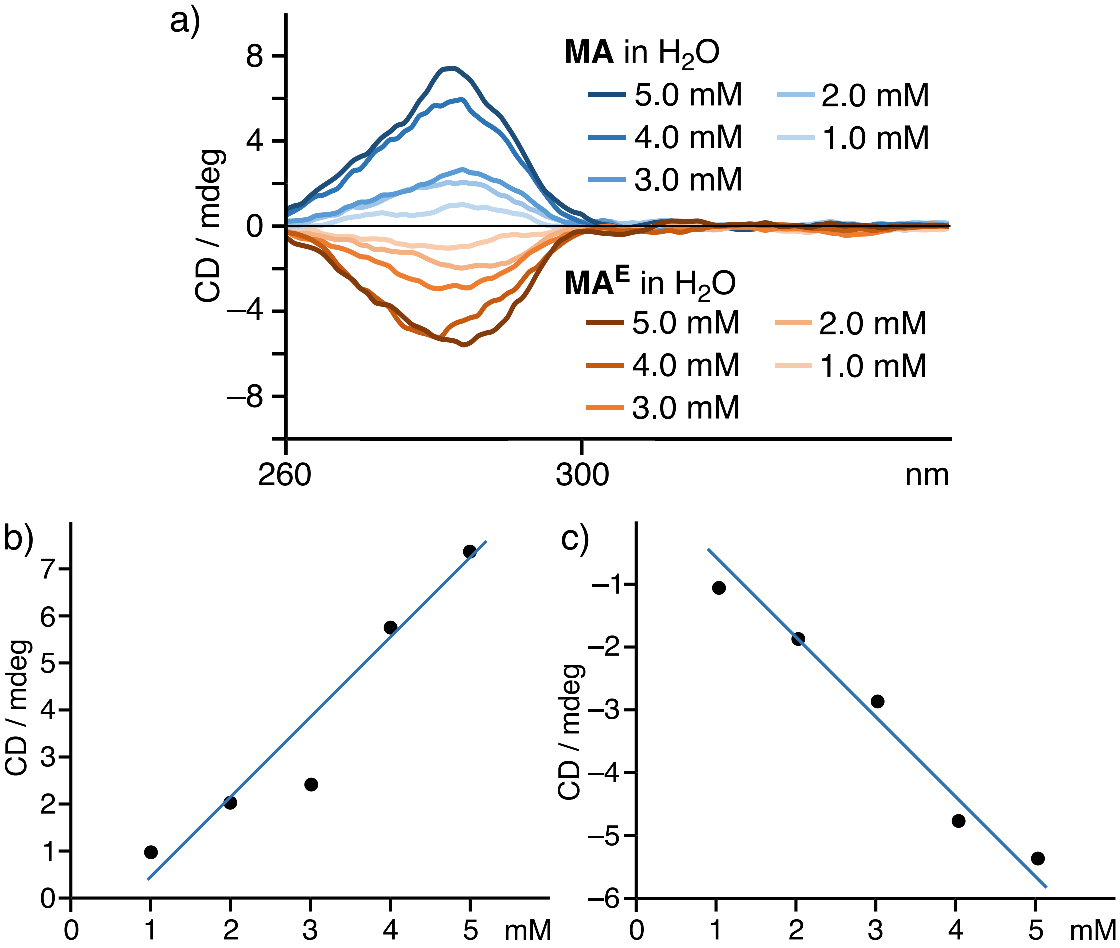

Figure S29. a) CD spectra (r.t., 1.0, 2.0, 3.0, 4.0, and 5.0 mM) of **MA** and **MA**$^{\mathbf{E}}$ in H_2O. Plots of the CD intensity (282 nm) versus concentration of b) **MA** and c) **MA**$^{\mathbf{E}}$.

Formation of (MA)$_n$•(TPE)$_m$ YH524

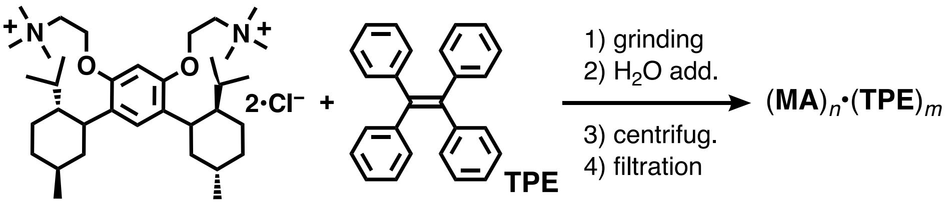

A mixture of **MA** (1.3 mg, 2.0 μmol) and tetraphenylethene (**TPE**; 0.7 mg, 2.0 μmol) was ground for 2.0 min using an agate mortar and pestle.[S3] After the addition of H_2O (2.0 mL) to the mixture, the suspended solution was centrifuged (14,800 rpm, 10 min) and then filtered by a membrane filter (pore size: 200 nm) to give a clear colorless solution of (**MA**)$_n$•(**TPE**)$_m$. The product structure was confirmed by UV-visible and DLS (d = 3.9 nm) analyses. After freeze drying, the concentration of encapsulated **TPE** was estimated to be 0.38 mM by ^{1}H NMR analysis ($CDCl_3$). In the same way, (**MA**)$_n$•(**HPS**)$_m$ was obtained from **MA** (1.3 mg, 2.0 μmol) and 1,1,2,3,4,5-hexaphenylsilole (**HPS**; 1.1 mg, 2.0 μmol).

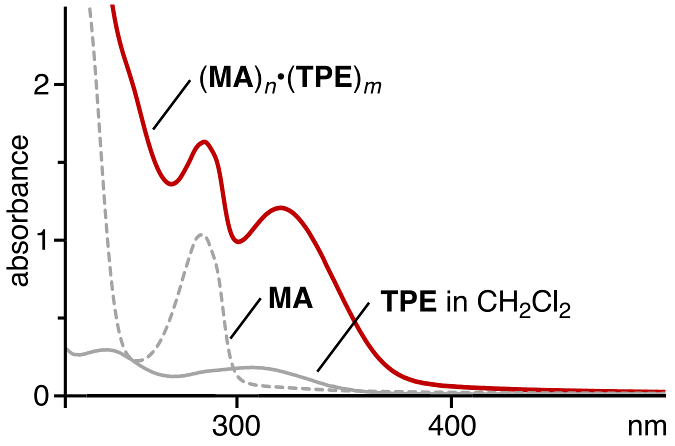

Figure S30. UV-visible spectra (H_2O, r.t., 1.0 mM based on **MA**) of (**MA**)$_n$•(**TPE**)$_m$, **MA**, and **TPE** in CH_2Cl_2.

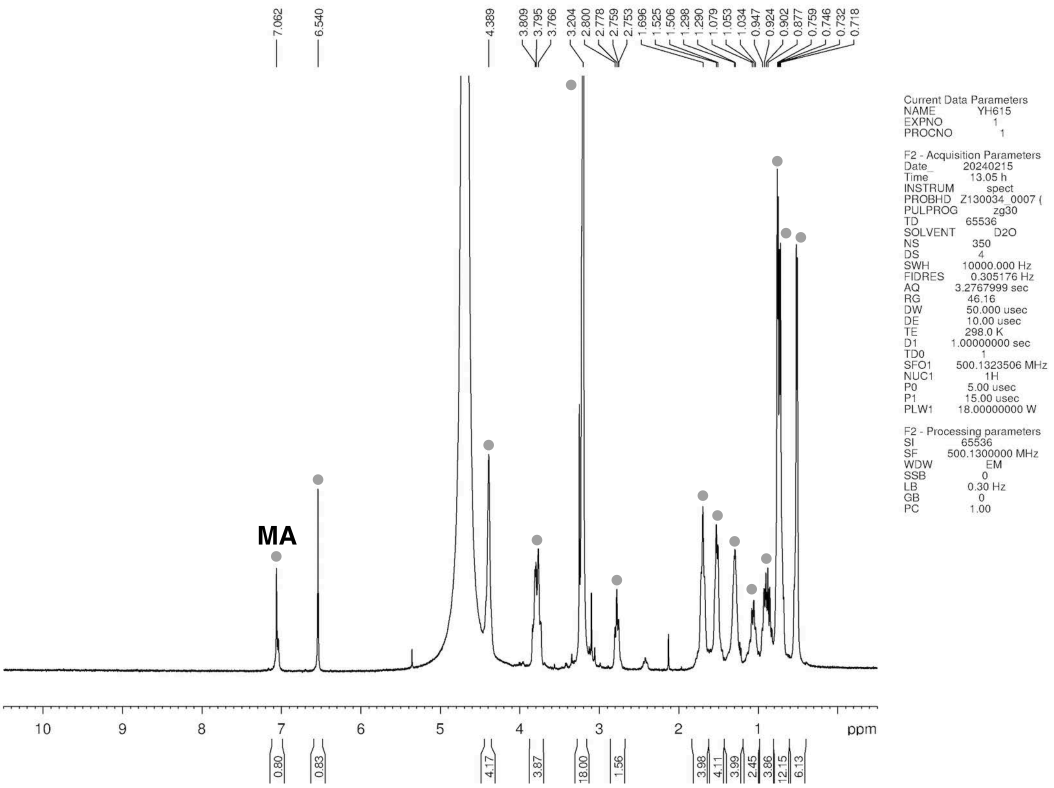

Figure S31a. ^{1}H NMR spectrum (500 MHz, D_2O, r.t., 1.0 mM based on **MA**) of (**MA**)$_n$•(**TPE**)$_m$ (gray circles: **MA**).

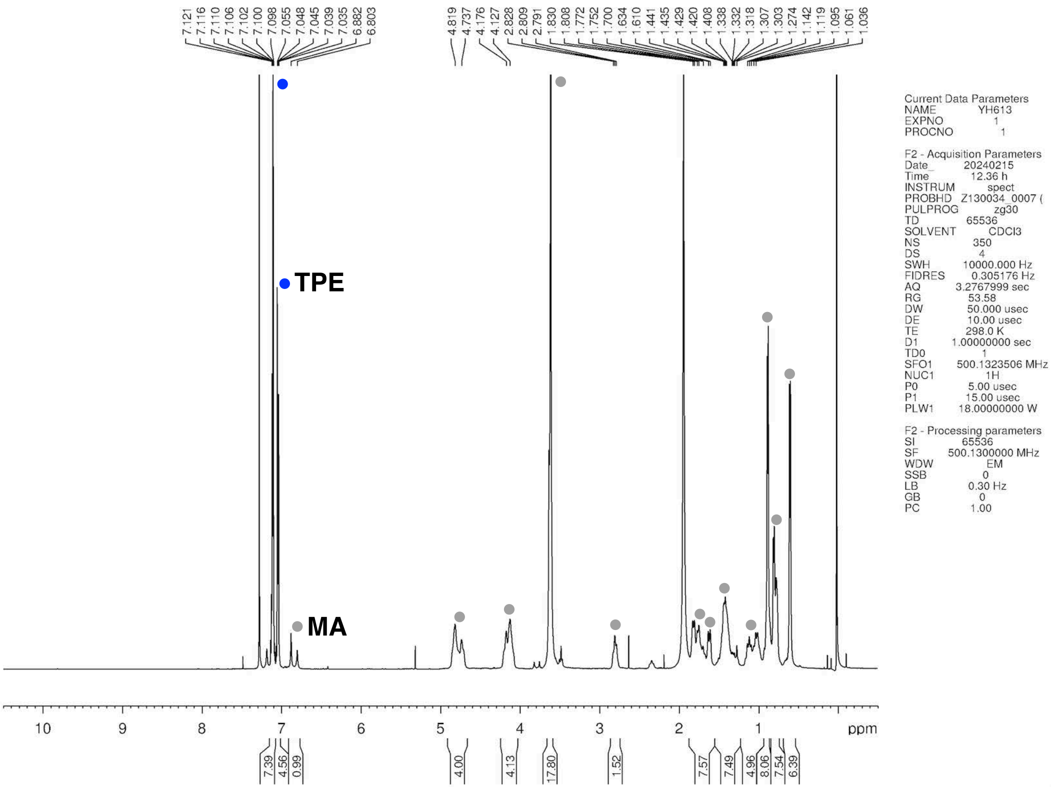

Figure S31b. ^{1}H NMR spectrum (500 MHz, $CDCl_3$, r.t.) of isolated (**MA**)$_n$•(**TPE**)$_m$ (gray circles: **MA**, blue circles: **TPE**).

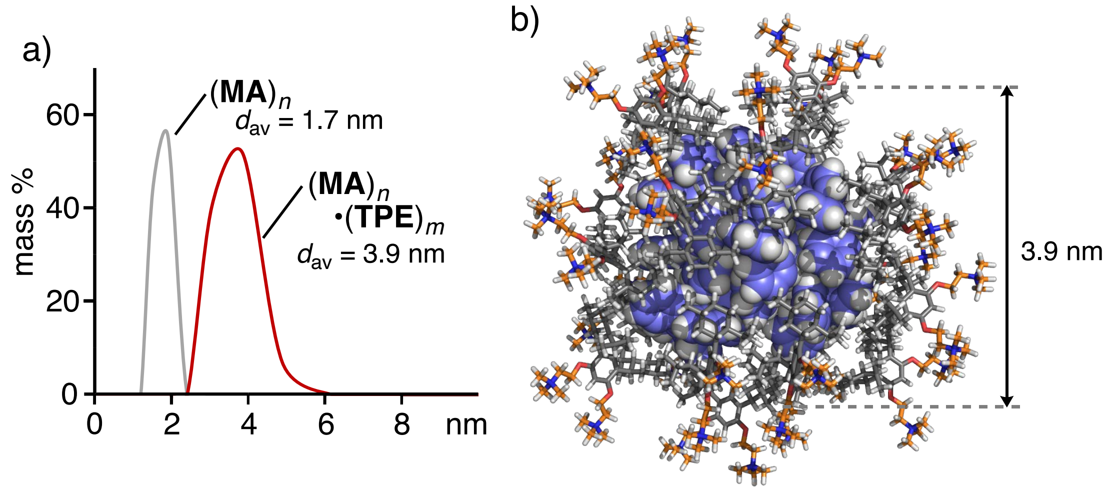

Figure S32. a) DLS charts (H_2O, r.t., 1.0 or 25 mM based on **MA**) of (**MA**)$_n$•(**TPE**)$_m$ and (**MA**)$_n$. b) Optimized structure of (**MA**)$_{21}$•(**TPE**)$_{12}$ (MM calculation).

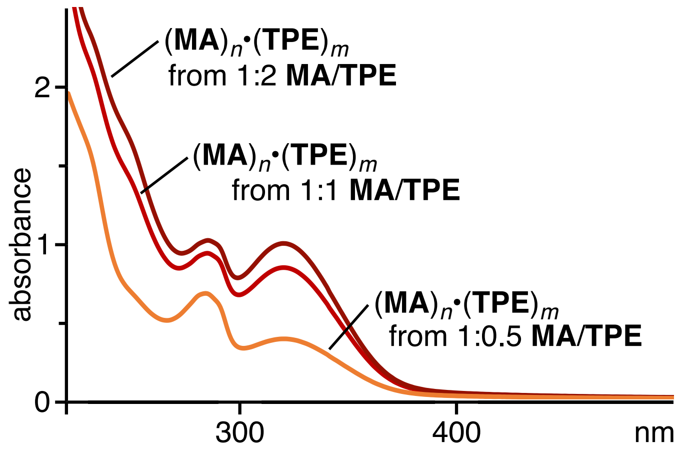

Figure S33. UV-visible spectra (H_2O, r.t., 1.0 mM based on **MA**) of (**MA**)$_n$•(**TPE**)$_m$ prepared from **MA** and **TPE** using their different molar ratios.

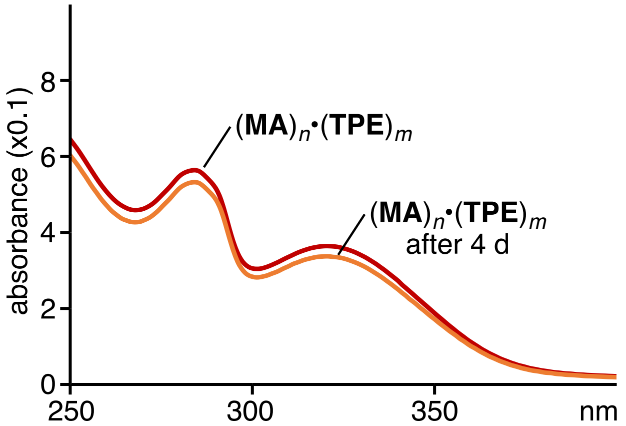

Figure S34. UV-visible spectra (H_2O, r.t., 1.0 mM based on **MA**) of (**MA**)$_n$•(**TPE**)$_m$ before and after 4 d.

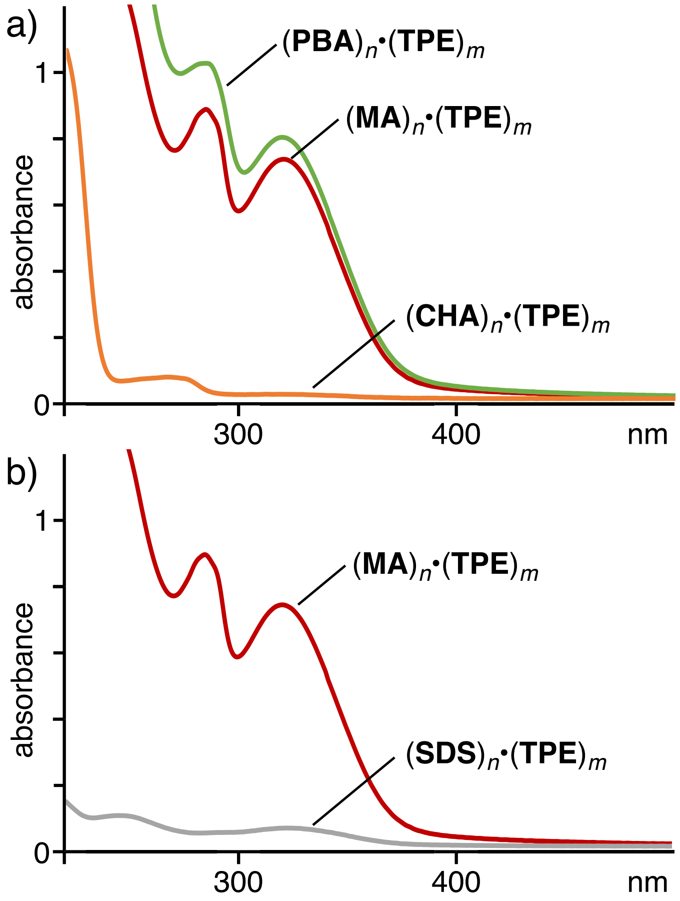

Figure S35. UV-visible spectra (H_2O, r.t., 1.0 mM based on amphiphiles) of a) (**MA** or **PBA** or **CHA**)$_n$•(**TPE**)$_m$ and b) (**MA** or **SDS**)$_n$•(**TPE**)$_m$.

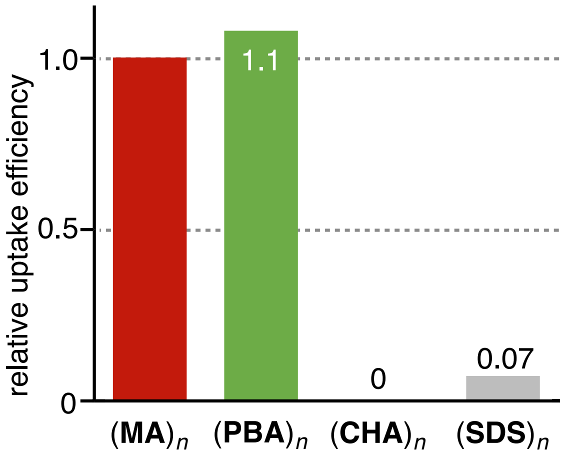

Figure S36. Relative uptake efficiencies of various micelles toward **TPE** in water.

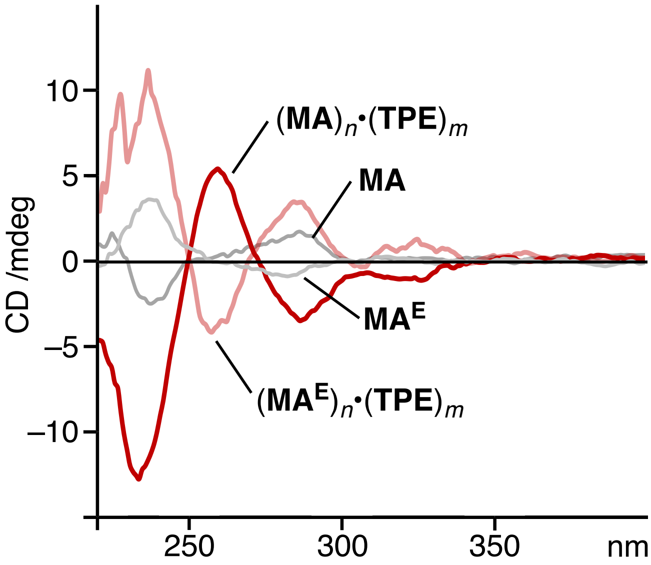

Figure S37. CD spectra (H_2O, r.t., 1.0 mM based on **MA**) of (**MA**)$_n$•(**TPE**)$_m$, (**MA**^{E})$_n$•(**TPE**)$_m$, **MA**, and **MA**^{E}.

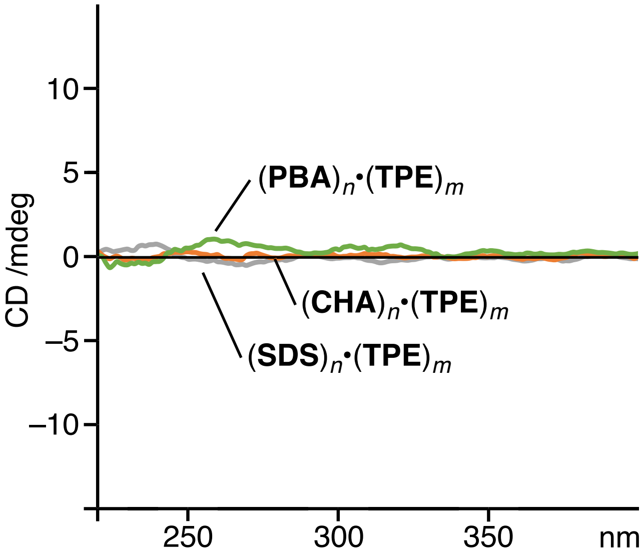

Figure S38. CD spectra (H_2O, r.t., 1.0 mM based on **MA**) of (**PBA**)$_n$•(**TPE**)$_m$, (**CHA**)$_n$•(**TPE**)$_m$, and (**SDS**)$_n$•(**TPE**)$_m$

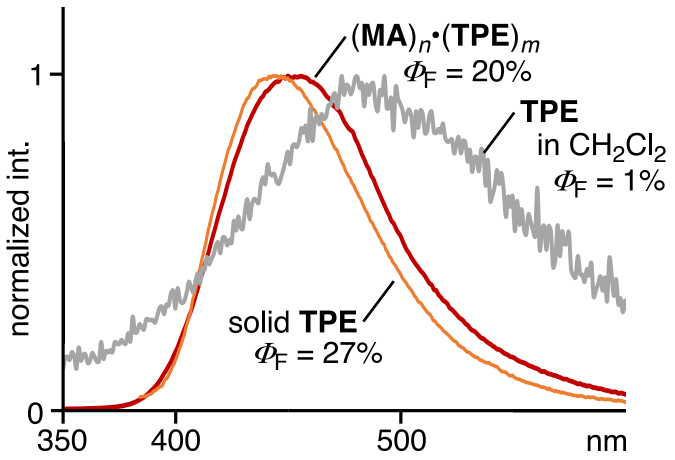

Figure S39. Fluorescence spectra (r.t., λ_{ex} = 320 nm) of (**MA**)$_n$•(**TPE**)$_m$ in H_2O (1.0 mM based on **MA**), solid **TPE**, and **TPE** in CH_2Cl_2 (0.1 mM), and their emission quantum yields.

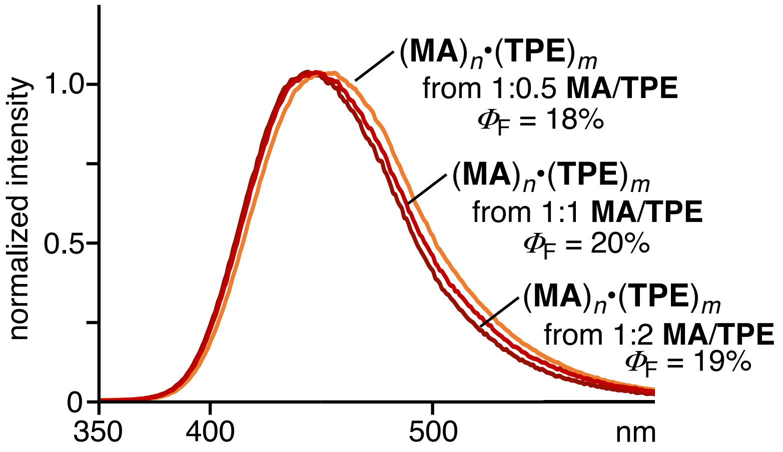

Figure S40. Fluorescence spectra (H_2O, r.t., λ_{ex} = 320 nm, 1.0 mM based on **MA**) of (**MA**)$_n$•(**TPE**)$_m$ prepared from **MA** and **TPE** in different molar ratios.

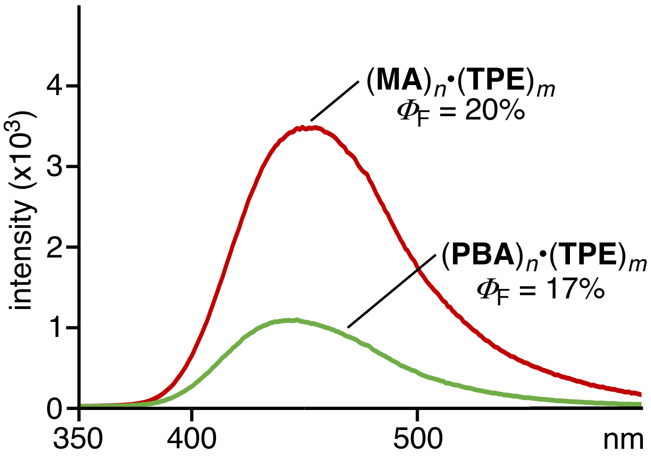

Figure S41. Fluorescence spectra (H_2O, r.t., λ_{ex} = 320 nm, 1.0 mM based on amphiphiles) of (**MA** or **PBA**)$_n$•(**TPE**)$_m$ and their emission quantum yields.

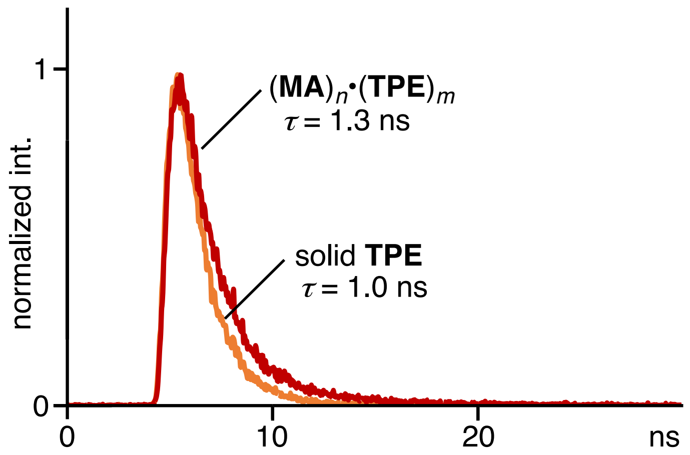

Figure S42. Fluorescence decay profiles (H_2O, r.t., λ_{ex} = 280 nm, λ_{det} = 450 nm, 1.0 mM based on **MA**) of (**MA**)$_n$•(**TPE**)$_m$ and solid **TPE**.

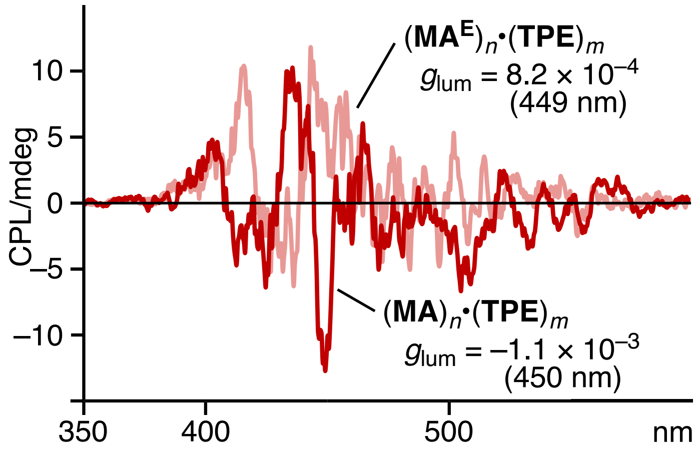

Figure S43. CPL spectra (H_2O, r.t., λ_{ex} = 320 nm, 1.0 mM based on amphiphiles) of (**MA**)$_n$•(**TPE**)$_m$ and (**MA**$^{\mathbf{E}}$)$_n$•(**TPE**)$_m$.

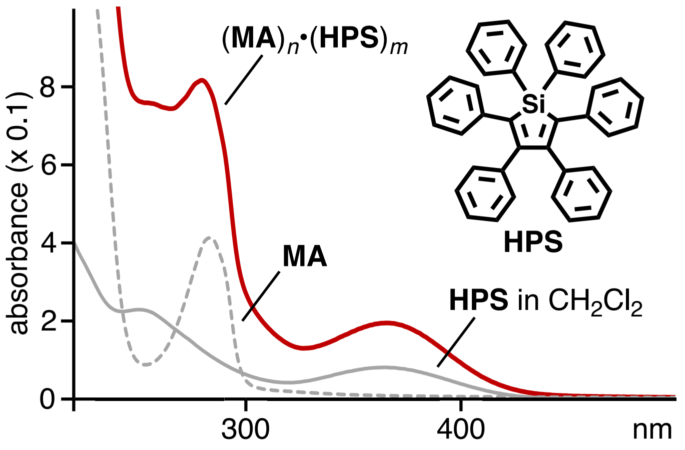

Figure S44. UV-visible spectra (H_2O, r.t., 1.0 mM based on **MA**) of (**MA**)$_n$•(**HPS**)$_m$, **MA**, and **HPS** in CH_2Cl_2.

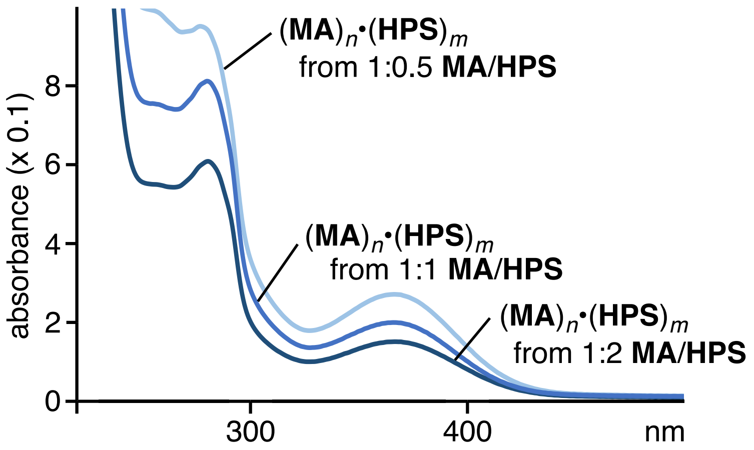

Figure S45. UV-visible spectra (H_2O, r.t., 1.0 mM based on **MA**) of (**MA**)$_n$•(**HPS**)$_m$ prepared from **MA** and **HPS** in different molar ratios.

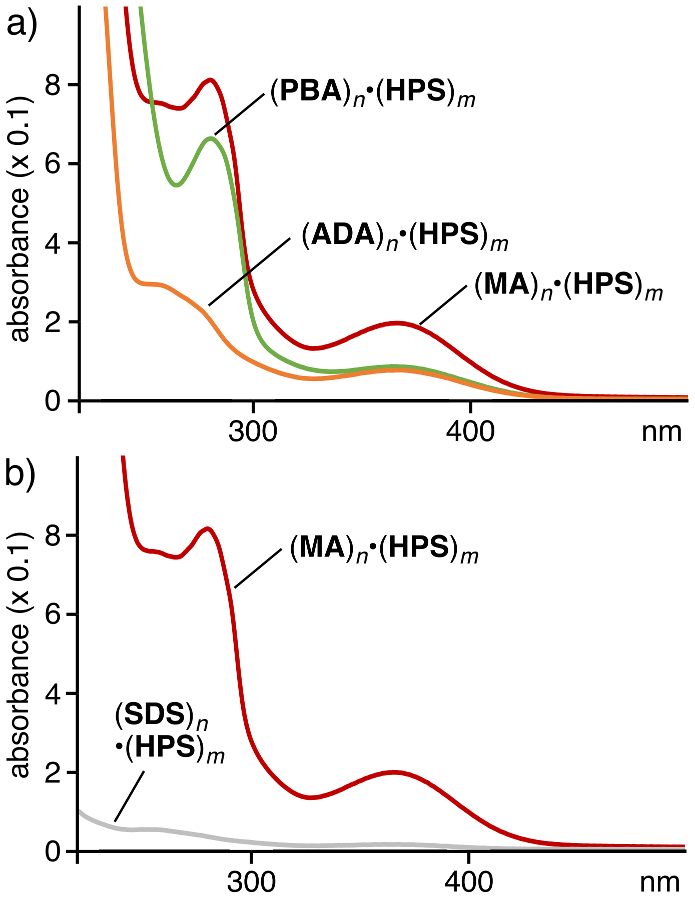

Figure S46. UV-visible spectra (H_2O, r.t., 1.0 mM based on amphiphiles) of a) (**MA** or **PBA** or **ADA**)$_n$•(**HPS**)$_m$ and b) (**MA** or **SDS**)$_n$•(**HPS**)$_m$.

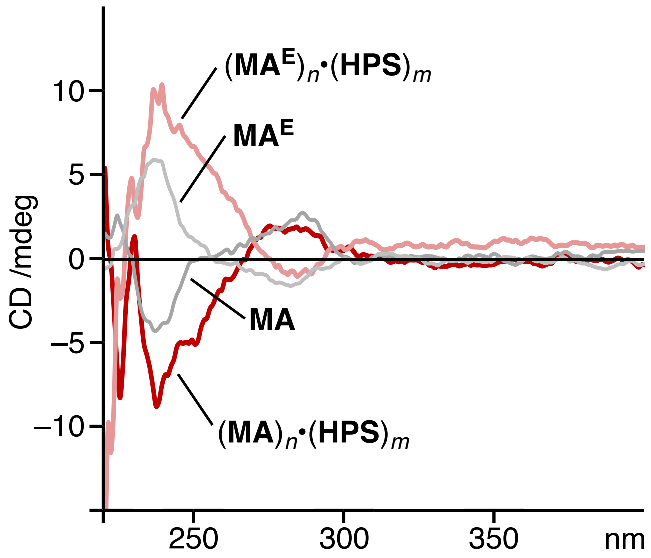

Figure S47. CD spectra (H_2O, r.t., 1.0 mM based on **MA**) of **(MA)**$_n$**•(HPS)**$_m$, **(MA**^{E}**)**$_n$**•(HPS)**$_m$, **MA**, and **MA**^{E}.

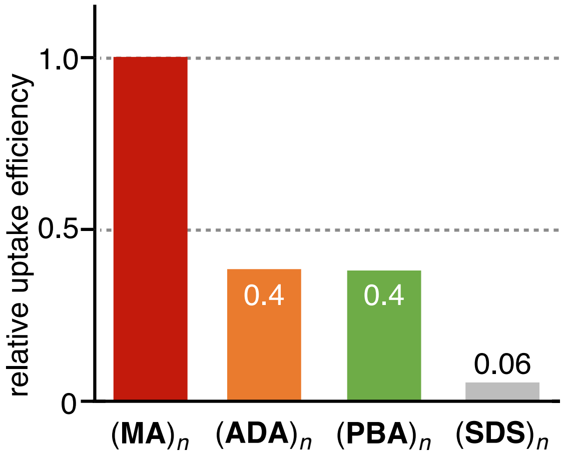

Figure S48. Relative uptake efficiencies of various micelles toward **HPS** in water.

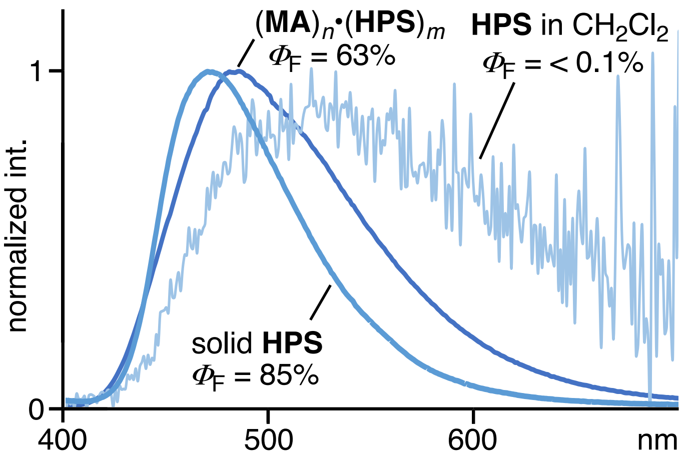

Figure S49. Fluorescence spectra (r.t., λ_{ex} = 366 nm) of **(MA)**$_n$**•(HPS)**$_m$ in H_2O (1.0 mM based on **MA**), solid **HPS**, and **HPS** in CH_2Cl_2 (0.1 mM) and their emission quantum yields.

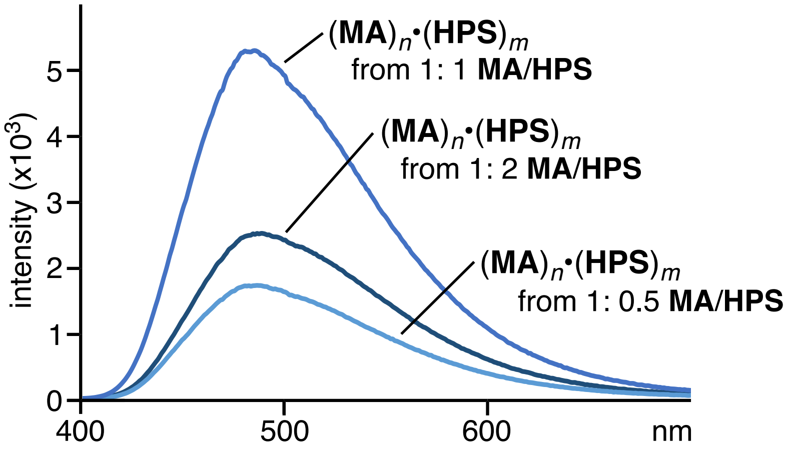

Figure S50. Fluorescence spectra (H_2O, r.t., λ_{ex} = 366 nm, 1.0 mM based on **MA**) of (**MA**)$_n$•(**HPS**)$_m$ prepared from **MA** and **HPS** in different molar ratios.

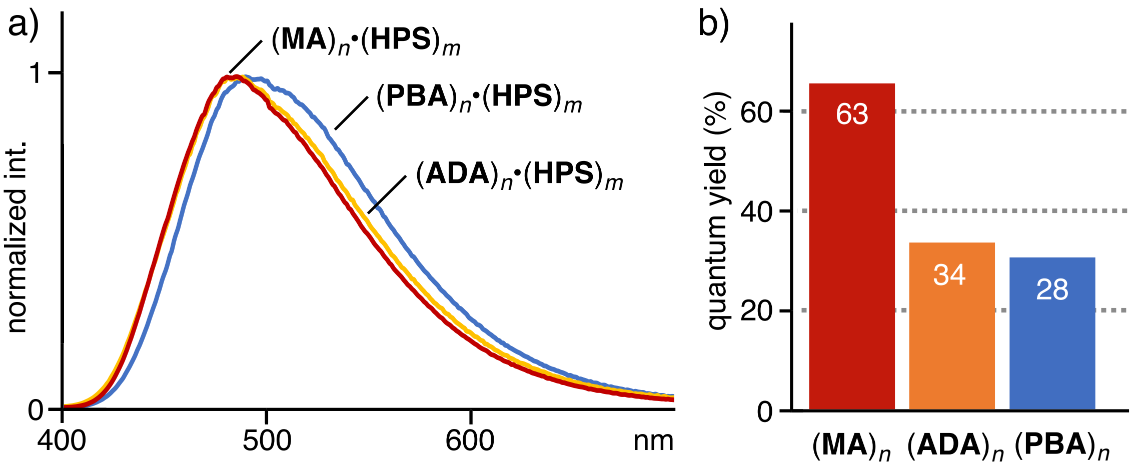

Figure S51. a) Fluorescence spectra (H_2O, r.t., λ_{ex} = 366 nm, 1.0 mM based on amphiphiles) of (**MA** or **PBA** or **ADA**)$_n$•(**HPS**)$_m$ and b) their emission quantum yields.

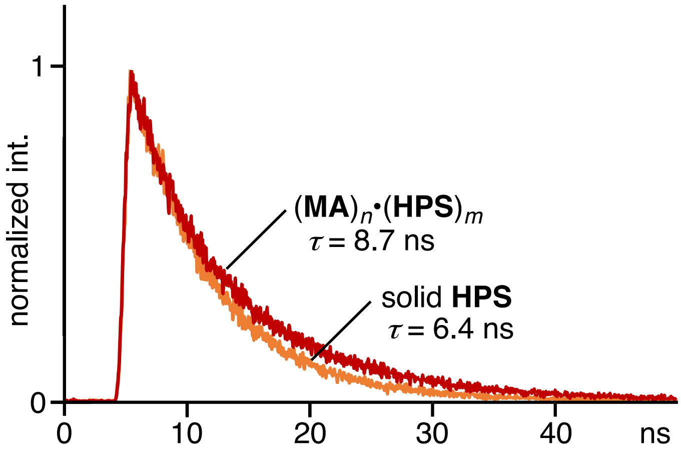

Figure S52. Fluorescence decay profiles (H_2O, r.t., λ_{ex} = 365 nm, λ_{det} = 490 nm, 1.0 mM based on **MA**) of (**MA**)$_n$•(**HPS**)$_m$ and solid **HPS**.

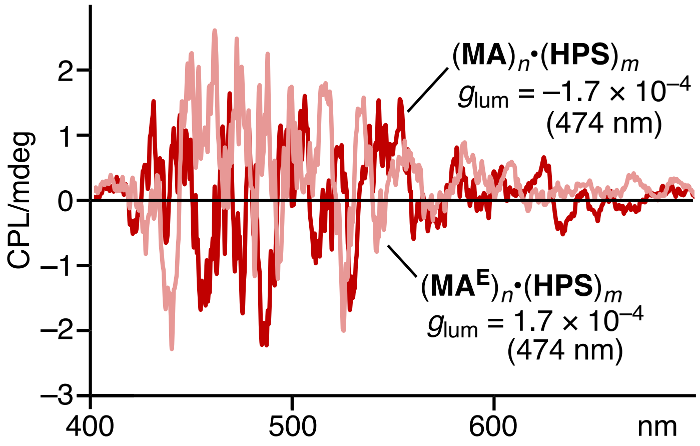

Figure S53. CPL spectra (H_2O, r.t., λ_{ex} = 366 nm, 1.0 mM based on amphiphiles) of **(MA)**$_n$**•(HPS)**$_m$ and **(MA**^{E}**)**$_n$**•(HPS)**$_m$.

Formation of (MA)$_n$•(Cor)$_m$ YH457

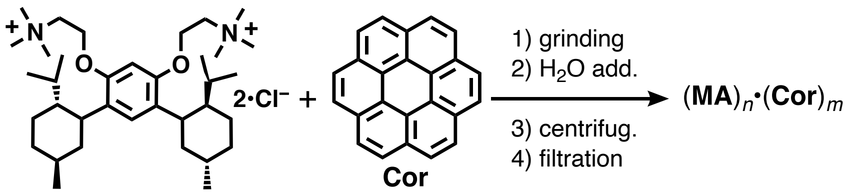

A mixture of **MA** (1.3 mg, 2.0 μmol) and coronene (**Cor**; 0.6 mg, 2.0 μmol) was ground for 2.0 min using an agate mortar and pestle.[S3] After the addition of H_2O (2.0 mL) to the mixture, the suspended solution was centrifuged (14,800 rpm, 10 min) and then filtered by a membrane filter (pore size: 200 nm) to give a clear colorless solution of **(MA)**$_n$**•(Cor)**$_m$. The product structure was confirmed by UV-visible and DLS (d = 3.5 nm) analyses. In the same way, **(MA)**$_n$**•(Per)**$_m$ was obtained from **MA** (1.3 mg, 2.0 μmol) and perylene (**Per**; 0.5 mg, 2.0 μmol).

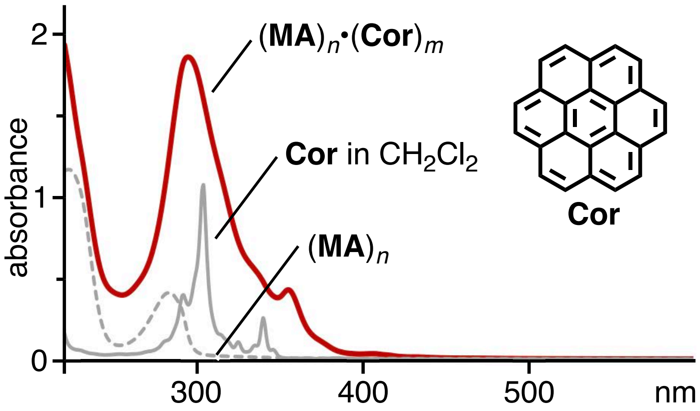

Figure S54a. UV-visible spectra (H_2O, r.t., 1.0 mM based on **MA**) of **(MA)**$_n$**•(Cor)**$_m$, **MA**, and **Cor** in CH_2Cl_2.

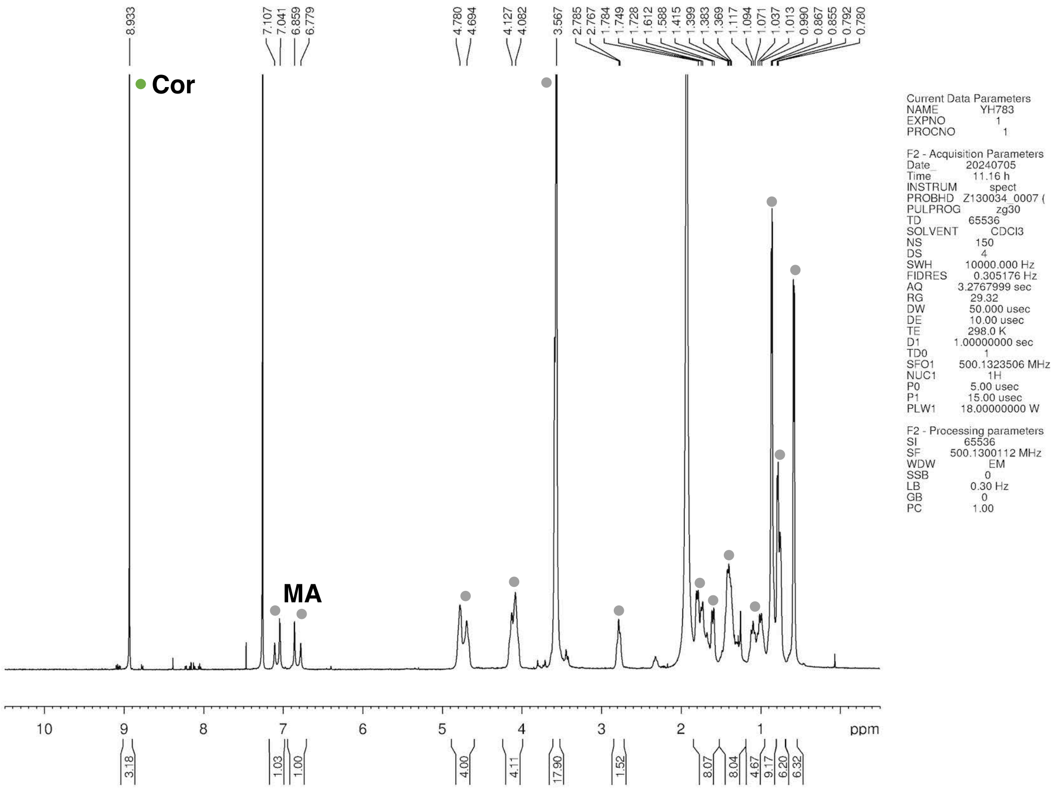

Figure S54b. ^{1}H NMR spectrum (500 MHz, $CDCl_3$, r.t.) of isolated (**MA**)$_n$•(**Cor**)$_m$ (gray circles: **MA**, green circle: **Cor**).

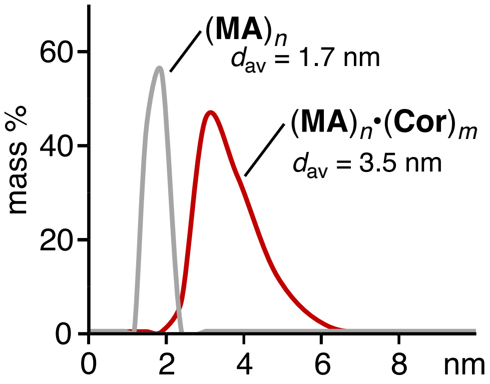

Figure S55. DLS charts (H_2O, r.t., 1.0 or 25 mM based on **MA**) of (**MA**)$_n$•(**Cor**)$_m$ and (**MA**)$_n$.

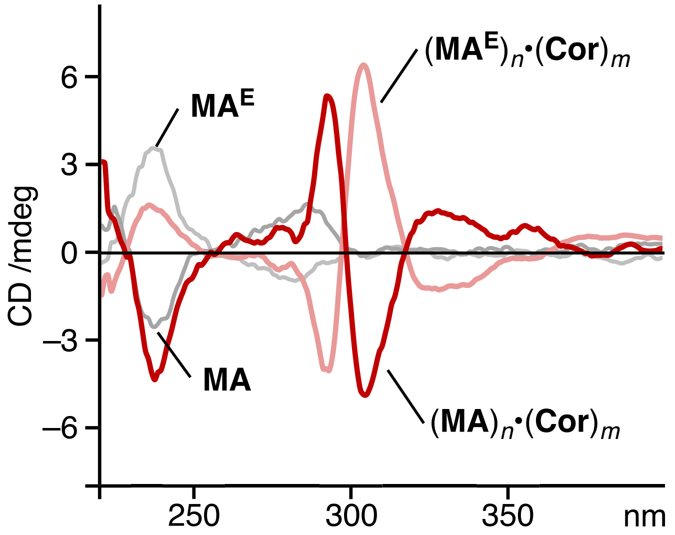

Figure S56. CD spectra (H_2O, r.t., 1.0 mM based on amphiphiles) of **(MA)**$_n$**•(Cor)**$_m$, **(MA**^{E}**)**$_n$**•(Cor)**$_m$, **MA**, and **MA**^{E}.

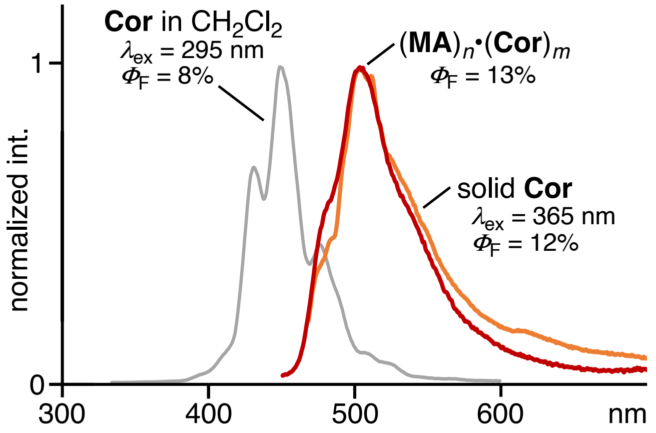

Figure S57. Fluorescence spectra (H_2O, r.t., λ_{ex} = 409 nm, 1.0 mM based on **MA**) of **(MA)**$_n$**•(Cor)**$_m$, solid **Cor**, and **Cor** in CH_2Cl_2 (0.1 mM).

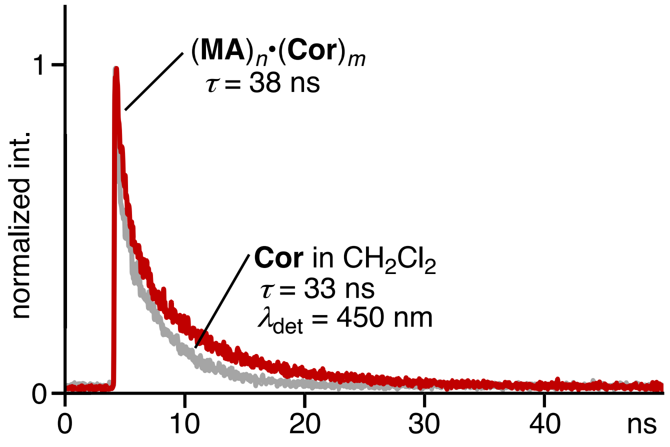

Figure S58. Fluorescence decay profiles (H_2O, r.t., λ_{ex} = 280 nm, λ_{det} = 500 nm, 1.0 mM based on **MA**) of **(MA)**$_n$**•(Cor)**$_m$ and **Cor** in CH_2Cl_2 (0.1 mM).

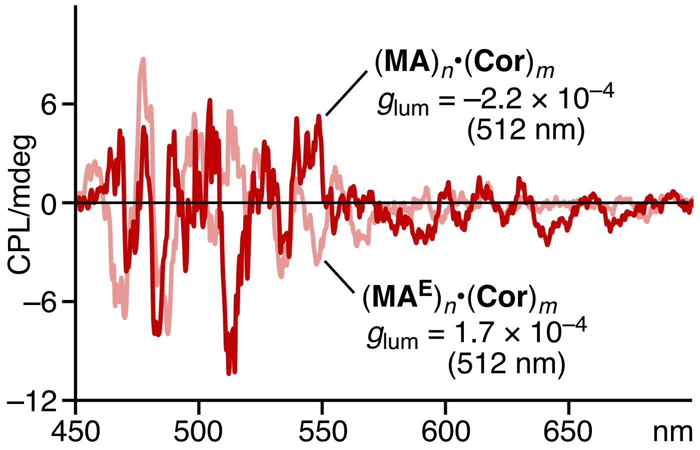

Figure S59. CPL spectra (H_2O, r.t., λ_{ex} = 295 nm, 1.0 mM based on amphiphiles) of **(MA)**$_n$**•(Cor)**$_m$ and **(MA^E)**$_n$**•(Cor)**$_m$.

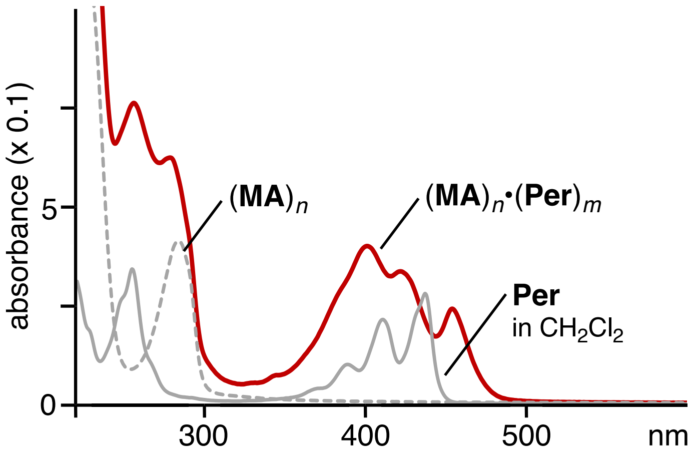

Figure S60. UV-visible spectra (H_2O, r.t., 1.0 mM based on **MA**) of **(MA)**$_n$**•(Per)**$_m$, **(MA)**$_n$, and **Per** in CH_2Cl_2 (0.1 mM).

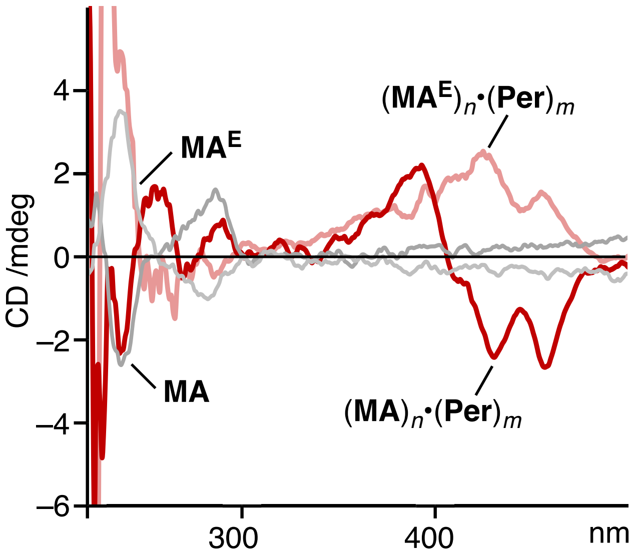

Figure S61. CD spectra (H_2O, r.t., 1.0 mM based on amphiphiles) of **(MA)**$_n$**•(Per)**$_m$, **(MA^E)**$_n$**•(Per)**$_m$, **MA**, and **MA^E**.

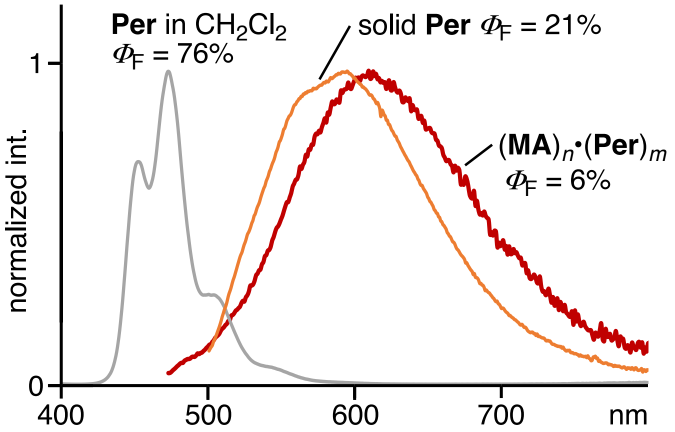

Figure S62. Emission spectra (r.t., λ_{ex} = 454 nm) of (**MA**)$_n$•(**Per**)$_m$ in water (1.0 mM based on **MA**), solid **Per**, and **Per** in CH_2Cl_2 (0.1 mM).

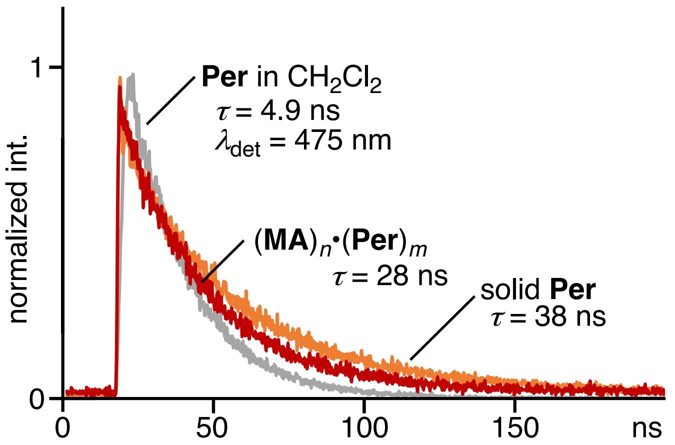

Figure S63. Emission decay profiles (H_2O, r.t., λ_{ex} = 405 nm, λ_{det} = 610 nm, 1.0 mM based on **MA**) of (**MA**)$_n$•(**Per**)$_m$, solid **Per**, and **Per** in CH_2Cl_2 (0.1 mM).

Formation of (MA)$_n$•(DBB)$_m$ YH465

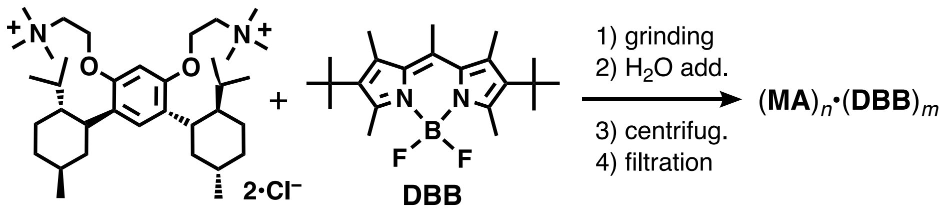

A mixture of **MA** (1.3 mg, 2.0 μmol) and di(*tert*-butyl)pentamethyl borondipyrromethene (**DBB**; 0.8 mg, 2.0 μmol) was ground for 2 min using an agate mortar and pestle.[S3] After the addition of H_2O (2.0 mL) to the mixture, the suspended solution was centrifuged (14,800 rpm, 10 min) and then filtered by a membrane filter (pore size: 200 nm) to give a clear red solution of (**MA**)$_n$•(**DBB**)$_m$. The product structure

was confirmed by UV-visible and DLS (d = 3.6 nm) analyses. After freeze drying, the concentration of encapsulated **DBB** was estimated to be 0.80 mM by ^{1}H NMR analysis. In the same way, (**MA**)$_n$•(**PMB**)$_m$ was obtained from **MA** (1.3 mg, 2.0 μmol) and pentamethyl borondipyrromethene (**PMB**; 0.5 mg, 2.0 μmol).

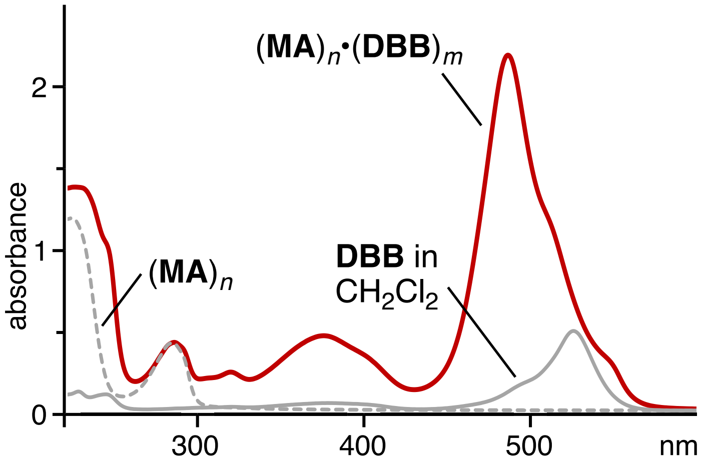

Figure S64. UV-visible spectra (H_2O, r.t., 1.0 mM based on **MA**) of (**MA**)$_n$•(**DBB**)$_m$, (**MA**)$_n$, and **DBB** in CH_2Cl_2 (0.1 mM).

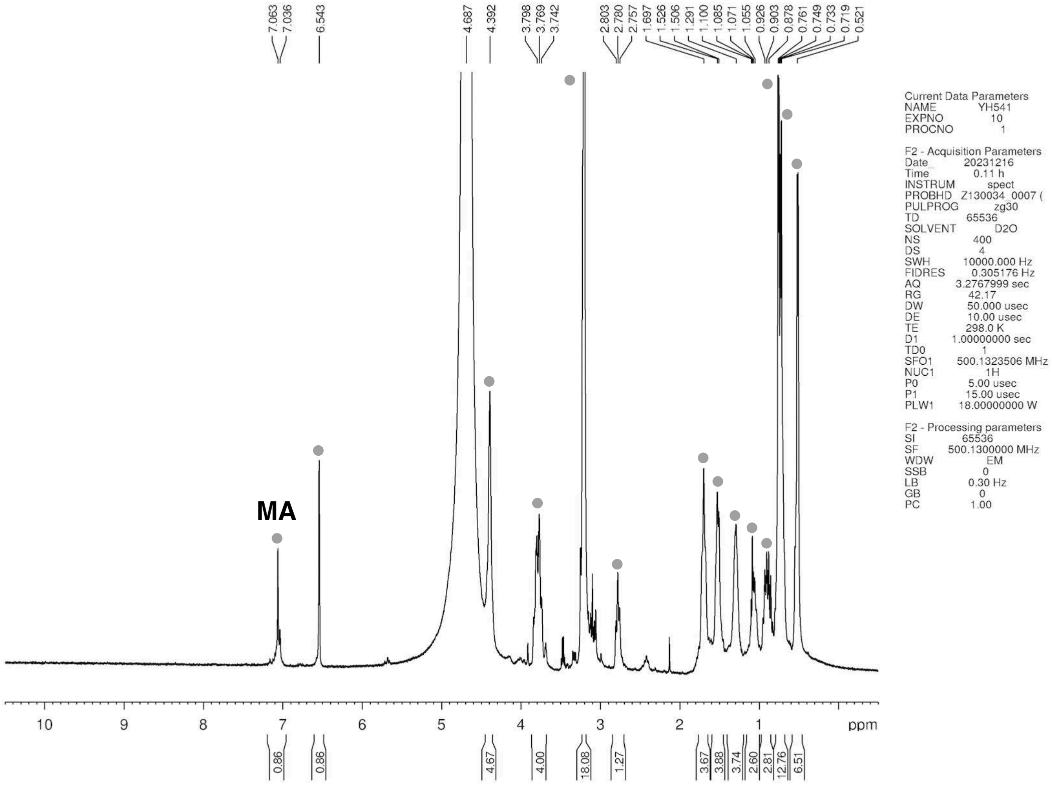

Figure S65a. ^{1}H NMR spectrum (500 MHz, D_2O, r.t., 1.0 mM based on **MA**) of (**MA**)$_n$•(**DBB**)$_m$ (gray circles: **MA**).

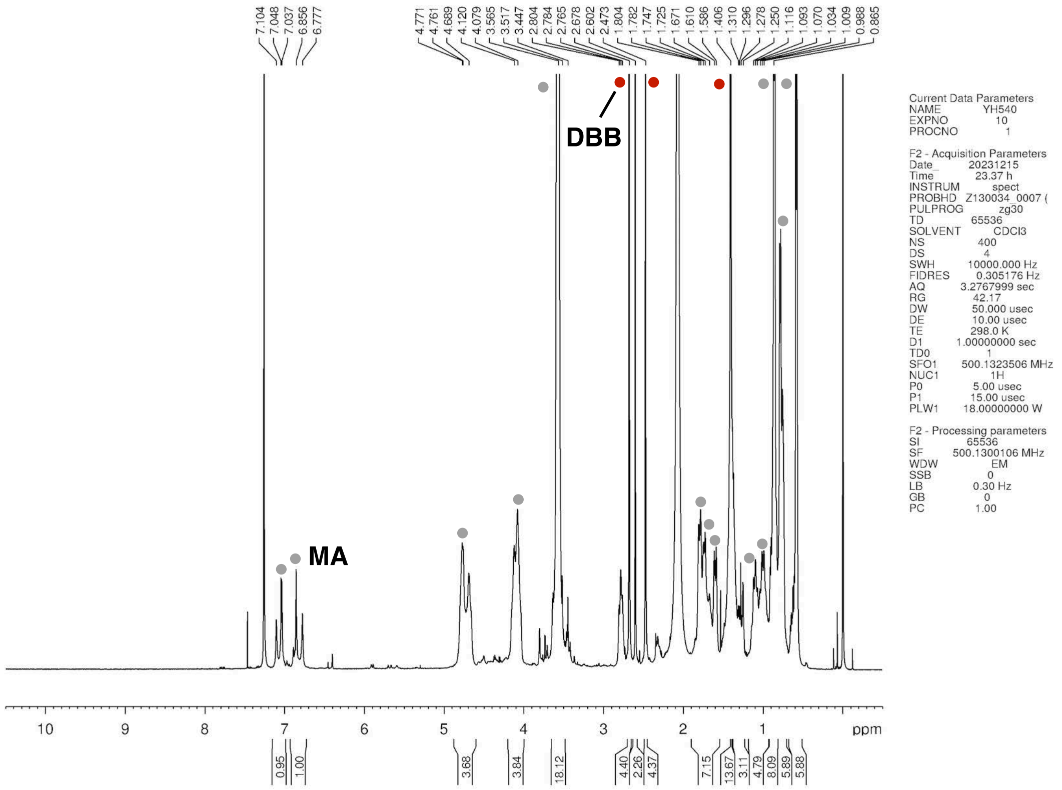

Figure S65b. ^{1}H NMR spectrum (500 MHz, $CDCl_3$, r.t.) of isolated $(\mathbf{MA})_n$•$(\mathbf{DBB})_m$ (gray circles: **MA**, red circles: **DBB**).

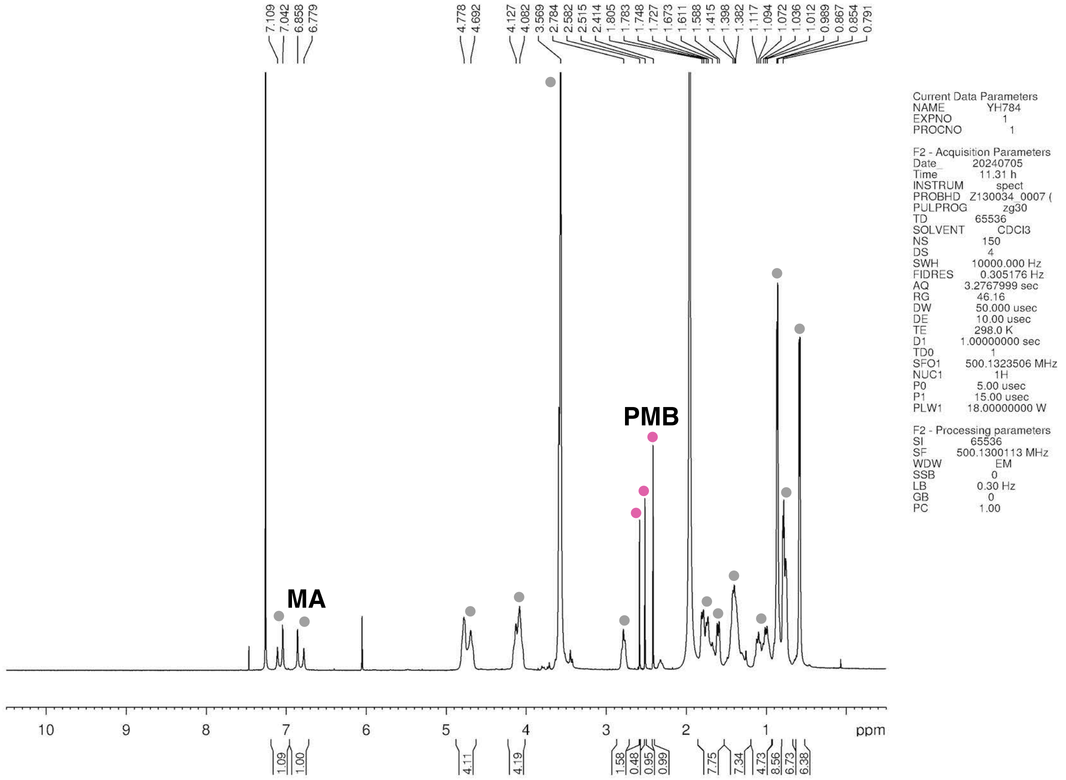

Figure S65c. ^{1}H NMR spectrum (500 MHz, $CDCl_3$, r.t.) of isolated $(\mathbf{MA})_n$•$(\mathbf{PMB})_m$ (gray circles: **MA**, pink circles: **PMB**).

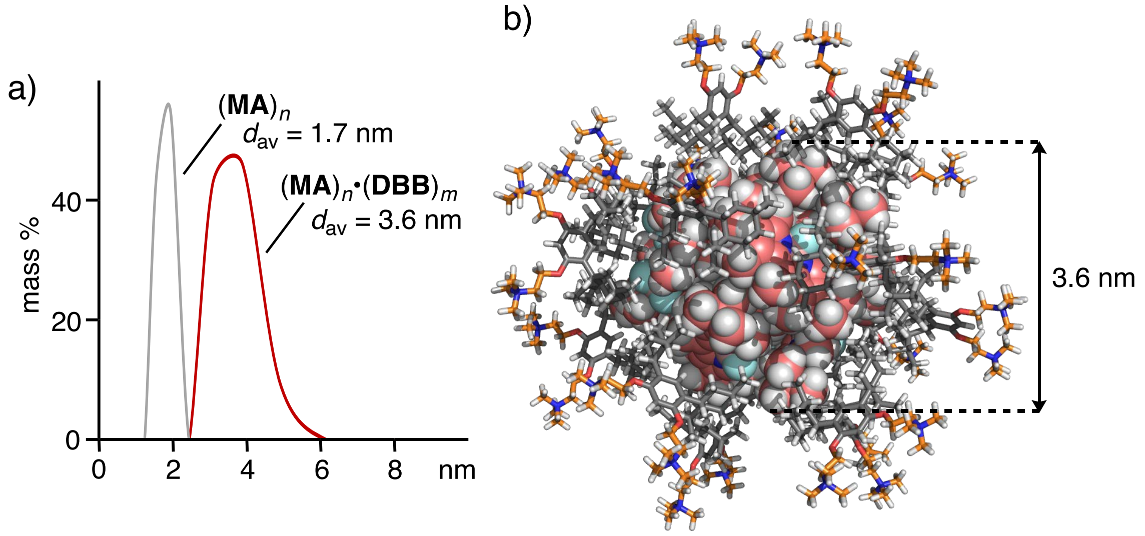

Figure S66. a) DLS charts (H_2O, r.t., 1.0 or 25 mM based on **MA**) of (**MA**)$_n$•(**DBB**)$_m$ and (**MA**)$_n$. b) Optimized structure of (**MA**)$_{14}$•(**DBB**)$_{10}$ (MM calculation).

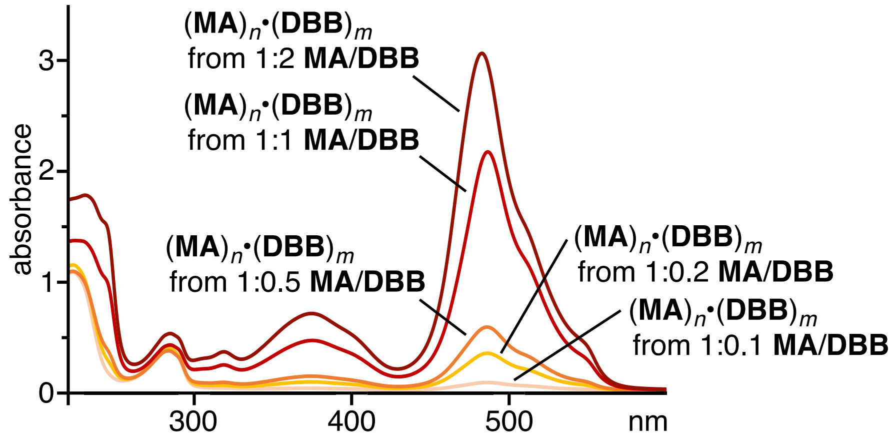

Figure S67. UV-visible spectra (H_2O, r.t., 1.0 mM based on **MA**) of (**MA**)$_n$•(**DBB**)$_m$ prepared from **MA** and **DBB** in different molar ratios.

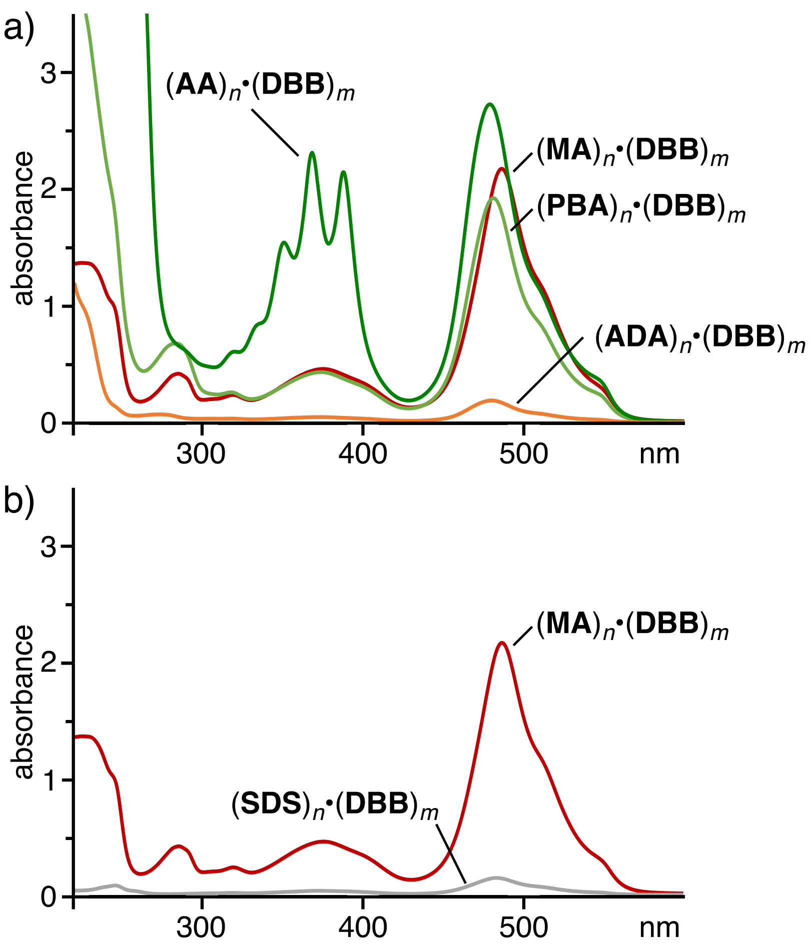

Figure S68. UV-visible spectra (H_2O, r.t., 1.0 mM based on amphiphiles) of a) (**MA** or **PBA** or **AA** or **ADA**)$_n$•(**DBB**)$_m$ and b) (**MA** or **SDS**)$_n$•(**DBB**)$_m$.

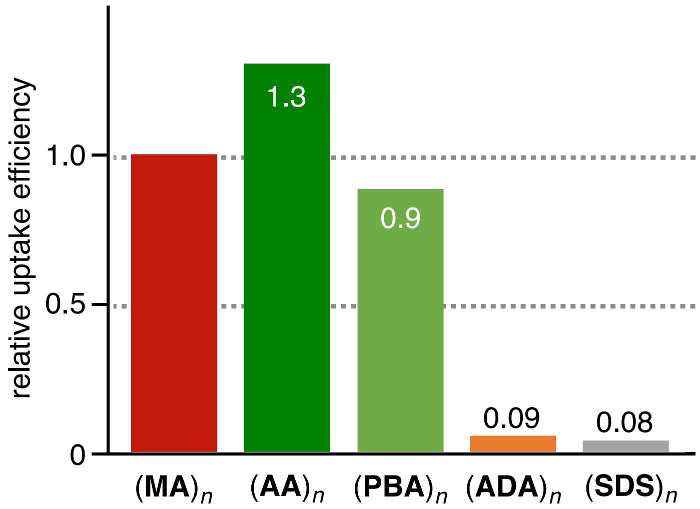

Figure S69. Relative uptake efficiencies of various micelles toward **DBB** in water.

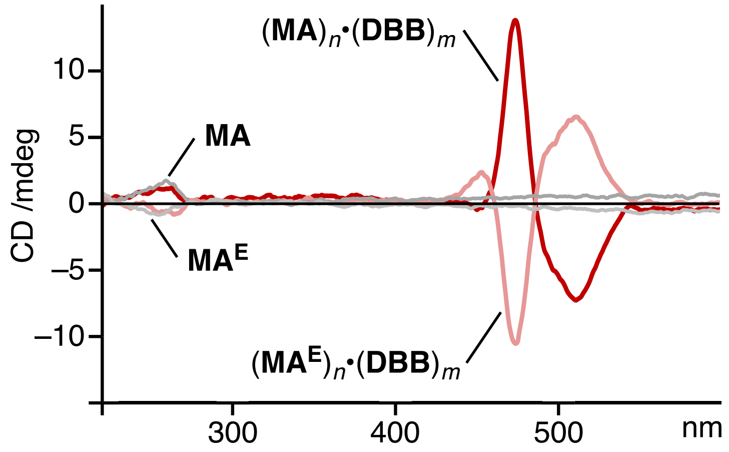

Figure S70. CD spectra (H_2O, r.t., 1.0 mM based on amphiphiles) of **(MA)**$_n$**•(DBB)**$_m$, **(MA**^{E}**)**$_n$**•(DBB)**$_m$, **MA**, and **MA**^{E}.

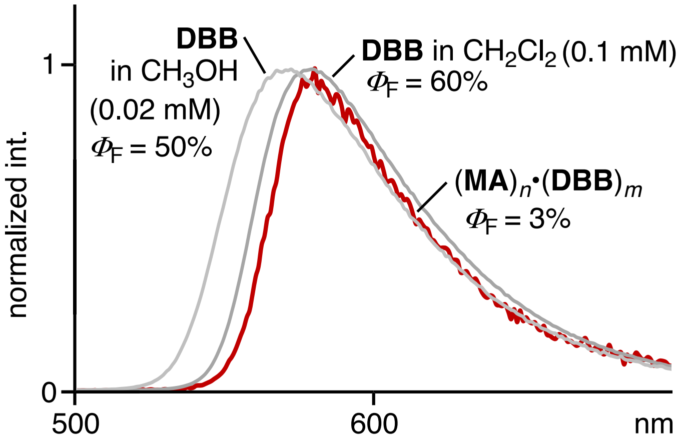

Figure S71. Fluorescence spectra (r.t., λ_{ex} = 486 nm) of **(MA)**$_n$**•(DBB)**$_m$ in water (1.0 mM based on **MA**) and **DBB** in CH_3OH (0.02 mM) and CH_2Cl_2 (0.1 mM).

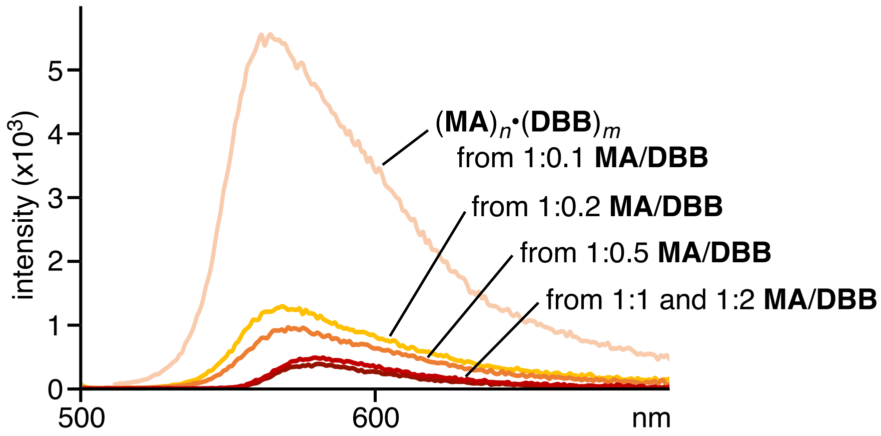

Figure S72. Fluorescence spectra (H_2O, r.t., λ_{ex} = 486 nm, 1.0 mM based on **MA**) of **(MA)**$_n$**•(DBB)**$_m$ prepared from **MA** and **DBB** in different molar ratios.

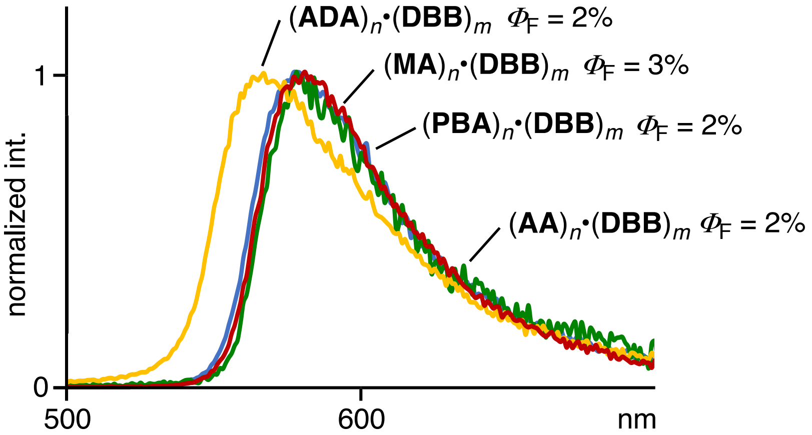

Figure S73. Fluorescence spectra (H_2O, r.t., λ_{ex} = 375 nm, 1.0 mM based on amphiphiles) of (**MA** or **PBA** or **ADA** or **AA**)$_n$•(**DBB**)$_m$.

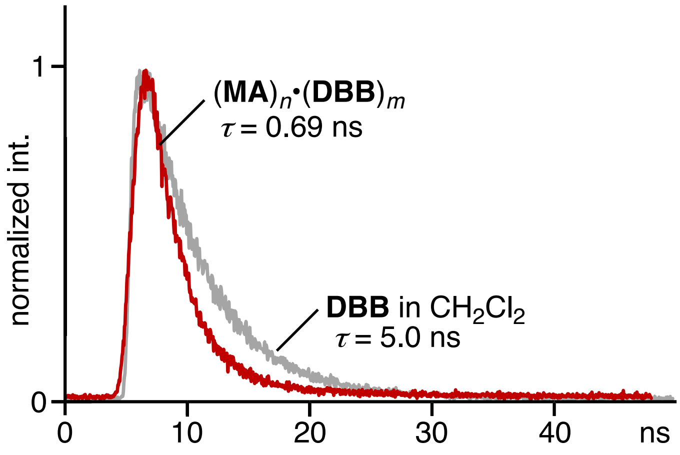

Figure S74. Fluorescence decay profiles (H_2O, r.t., λ_{ex} = 340 nm, λ_{det} = 580 nm, 1.0 mM based on **MA**) of (**MA**)$_n$•(**DBB**)$_m$ and **DBB** in CH_2Cl_2 (0.1 mM).

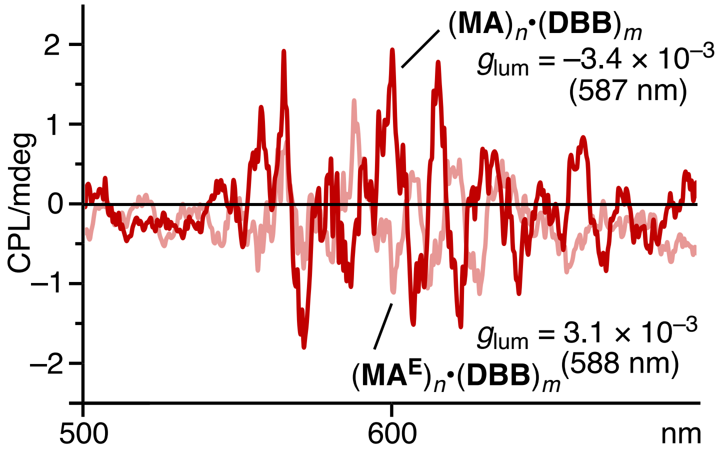

Figure S75. CPL spectra (H_2O, r.t., λ_{ex} = 320 nm, 1.0 mM based on **MA**) of (**MA**)$_n$•(**DBB**)$_m$ and (**MA**^{E})$_n$•(**DBB**)$_m$.

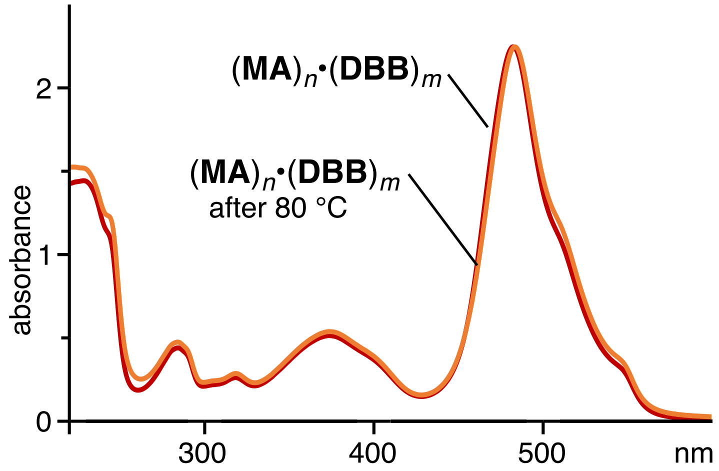

Figure S76a. UV-visible spectra (H_2O, r.t., 1.0 mM based on **MA**) of (**MA**)$_n$•(**DBB**)$_m$ before and after heating at 80 ºC for 5 min.

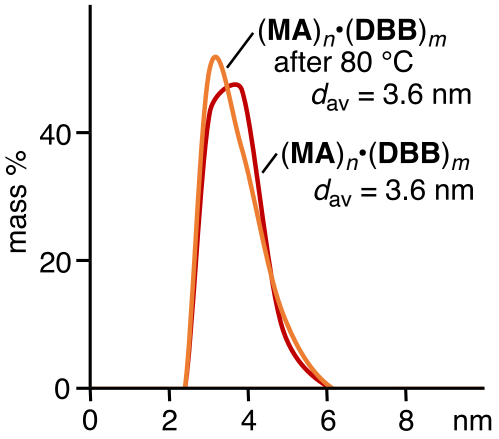

Figure S76b. DLS charts (H_2O, r.t., 1.0 mM based on **MA**) of (**MA**)$_n$•(**DBB**)$_m$, before and after heating at 80 ºC for 5 min.

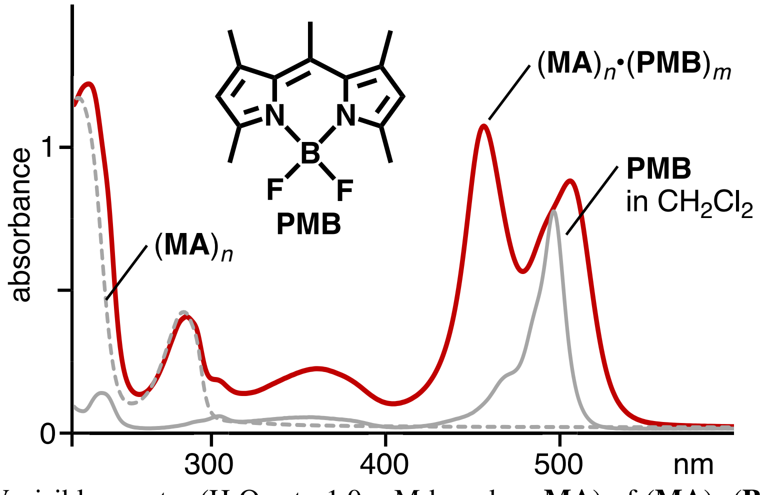

Figure S77. UV-visible spectra (H_2O, r.t., 1.0 mM based on **MA**) of (**MA**)$_n$•(**PMB**)$_m$, **MA** and **PMB** in CH_2 Cl_2.

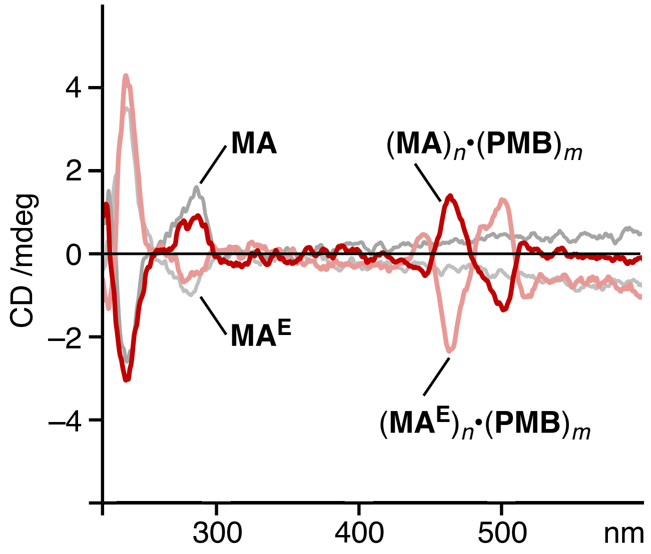

Figure S78. CD spectra (H_2O, r.t., 1.0 mM based on **MA**) of **(MA)**$_n$**•(PMB)**$_m$, **(MA**^{E}**)**$_n$**•(PMB)**$_m$, **MA**, and **MA**^{E}.

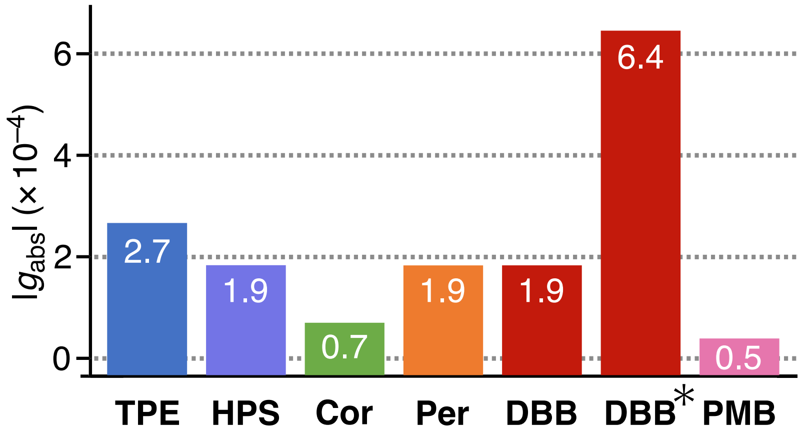

Figure S79a. Absolute absorption dissymmetry factors (H_2O, r.t., 1.0 mM based on **MA**) of host-guest composites **(MA)**$_n$ including dyes **TPE**, **HPS**, **Cor**, **Per**, **DBB**, and **PMB**, respectively (*: after heating at 80 °C).

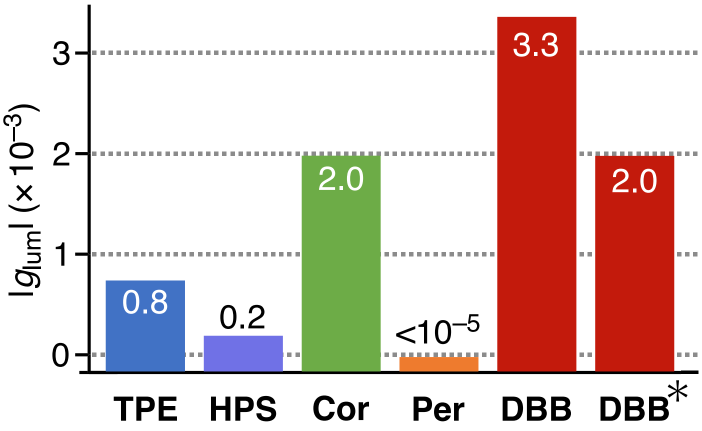

Figure S79b. Absolute luminescence dissymmetry factors (H_2O, r.t., 1.0 mM based on **MA**) of host-guest composites (**MA**)$_n$ including dyes **TPE**, **HPS**, **Cor**, **Per**, and **DBB**, respectively (*: after heating at 80 ºC).

Construction of optimized host-guest structures (general procedure)

The host-guest ratio of a product was determined by the ^{1}H NMR integral analysis of the isolated product after lyophilization and re-solubilization in organic solvent (e.g., $CDCl_3$). The average core diameter of the product was estimated by DLS analysis. On the basis of the obtained ratio and average diameter, a spherical core-shell structure was generated using the guest and amphiphilic molecules in the estimated ratio. The host-guest structure was optimized by molecular mechanics (MM) calculation (geometry optimization (quality: ultra-fine), Forcite module (COMPASS force field), BIOVIA Materials Studio 2020, version 20.1.0.5 (Dassault Systèmes Co.)). The structural optimizations were repeated until convergence of the total energy.
